# Characterization of depressive symptoms in individuals with spinal cord injury through the Beck Depression Inventory, and their role in recovery

**DOI:** 10.1038/s41598-026-66971-7

**Published:** 2026-08-20

**Authors:** Roman Schefzik, Laura Heutehaus, Christian Schuld, Tobias Gradinger, Oksana Berhe, Doris Maier, Marion Saur, Klaus Röhl, Rainer Abel, Chiara Pavese, Michael Baumberger, Armin Curt, Michèle Hubli, Harvinder S. Chhabra, EMSCI Study Group, Doris Maier, Marion Saur, Klaus Röhl, Rainer Abel, Chiara Pavese, Michael Baumberger, Armin Curt, Michèle Hubli, Harvinder S. Chhabra, Norbert Weidner, Udo Dannlowski, Tilo Kircher, Norbert Weidner, Emanuel Schwarz

**Affiliations:** 1 Hector Institute for Artificial Intelligence in Psychiatry, Central Institute of Mental Health, Medical Faculty Mannheim, Heidelberg University, Mannheim, Germany; 2 Department of Psychiatry and Psychotherapy, Central Institute of Mental Health, Medical Faculty Mannheim, Heidelberg University, Mannheim, Germany; 3 Spinal Cord Injury Center, Heidelberg University Hospital, Heidelberg, Germany; 4 Medical Faculty Heidelberg, Heidelberg University, Heidelberg, Germany; 5 Center for Spinal Cord Injuries, Berufsgenossenschaftliche Unfallklinik Murnau, Murnau, Germany; 6 Orthopädische Klinik Hessisch Lichtenau, Hessisch Lichtenau, Germany; 7 Zentrum für Rückenmarkverletzte und Klinik für Orthopädie, BG Klinikum Bergmannstrost Halle, Halle, Germany; 8 Clinic for Paraplegia, Klinikum Bayreuth GmbH, Bayreuth, Germany; 9 Department of Clinical-Surgical, Diagnostic and Pediatric Sciences, University of Pavia, Pavia, Italy; 10 Istituti Clinici Scientifici Maugeri IRCCS, Neurorehabilitation and Spinal Unit of Pavia Institute, Pavia, Italy; 11 Swiss Paraplegic Centre, Nottwil, Switzerland; 12 Spinal Cord Injury Center, Balgrist University Hospital, University of Zurich, Zurich, Switzerland; 13 Department of Spine and Rehabilitation, Sri Balaji Action Medical Institute, New Delhi, India; 14 Institute for Translational Psychiatry, University of Münster, Münster, Germany; 15 Department of Psychiatry, Medical School and University Medical Center OWL, Protestant Hospital of the Bethel Foundation, Bielefeld, Germany; 16 Department of Psychiatry and Psychotherapy, University of Marburg, Marburg, Germany

**Keywords:** Beck Depression Inventory, Depressive symptoms, Major depressive disorder, Neurological and functional recovery, Random forest, Spinal cord injury, Diseases, Neurology, Neuroscience, Psychology, Psychology

## Abstract

Depressive symptoms show an increased prevalence in individuals affected by spinal cord injury (SCI), yet their in-depth characterization and association with clinical outcomes remain underexplored. We investigated depressive symptoms, operationalized through the items in the Beck Depression Inventory (BDI), in a longitudinal, observational study of 165 individuals with SCI, and explored their trajectories and association with neurological and functional recovery. 134 and 43 individuals with major depressive disorder (MDD) diagnosis from two reference cohorts were investigated to evaluate similarities and differences of the clinical manifestation of depression. At baseline (one month after injury), 25% of individuals with SCI showed a clinically relevant degree of overall depressive symptoms, and 21% at follow-up (six or twelve months after injury). Predominant depressive symptoms involved physical-somatic phenomena such as sleep disturbances, fatigue, changes in eating habits, and reduced libido. In contrast, individuals with MDD diagnosis exhibited a broader range of symptoms, particularly those related to self-rejection and self-blame. A development of a clinically relevant degree of overall depressive symptoms from the baseline to the follow-up stage in individuals with SCI was primarily reflected in increases in BDI scores for items related to self-hatred, anhedonia, emotional overload, or cognitive stress. A random forest (RF) model using psychiatric, SCI-specific, and demographic baseline features to predict the presence of a clinically relevant degree of overall depressive symptoms at follow-up demonstrated only mediocre predictive performance. In contrast, similar RF models exhibited good performances in predicting neurological and functional recovery following SCI, relying primarily on SCI-specific (rather than psychiatric) baseline features. Depressive symptoms in individuals with SCI appeared to be largely unrelated to neurological and functional recovery and were primarily related to physical-somatic phenomena. This distinguishes them from the broader range of symptoms observed in individuals with MDD diagnosis, which more commonly encompass self-blame, self-hatred, and other cognitive-emotional dimensions. To help reduce the risk of undetected development of a clinically relevant degree of overall depressive symptoms during SCI treatment, it may be useful to focus particularly on cognitive-emotional symptoms, alongside physical-somatic symptoms, which may be affected by the direct consequences of the injury itself.

## Introduction

Spinal cord injury (SCI)^1^ causes motor, sensory or autonomic dysfunction and severely affects body functions below the level of injury. SCI can either be traumatic, e.g. as the result of a fall or a motor vehicle accident, or non-traumatic, e.g. caused by a tumor, ischemic event or inflammation^2^. Based on data of the Global Burden of Disease Study 2019, Ding et al.^3^ reported that globally, there were 0.9 million incident SCI cases and 20.6 million prevalent SCI cases in 2019, with males typically being more affected than females^3,4^. The distribution of age at injury typically shows two peaks, one around the 30s and another around the 50s, with the proportion of elderly people sustaining a traumatic SCI having increased remarkably over the last two decades^4^.

Major depressive disorder (MDD) is a debilitating mental illness characterized by depressed mood, reduced interest or pleasure in activities, cognitive impairments, and vegetative symptoms^5^. MDD is diagnosed based on the criteria in the most commonly used diagnostic manuals, DSM-5 and ICD-10. Standard tools to aid in the diagnostic process and to help quantify the severity of MDD are semi-structured interviews and questionnaires. A frequently used self-report questionnaire in this context, both in clinical practice and in research, is the multi-item multiple-choice Beck Depression Inventory (BDI)^6,7^.

Depressive symptoms are known to be associated with and affected by physical health^8,9^. This in particular holds for individuals with SCI. While SCI can have a substantial negative impact on mental health in general^10,11^, in particular, the prevalence of depressive symptoms after SCI is greater than in the general population. While 4.4*%* of the world’s population is affected by depression^12^, a meta-study by Williams and Murray^13^ reports a mean prevalence estimate of depression diagnosis after SCI at 22.2*%*, with lower- and upper-bound estimates of 18.7*%* and 26.3*%*, respectively. Depressive symptoms in SCI individuals can increase caregiver burden^14^ and are frequently accompanied by comorbid psychiatric conditions, leading to more healthcare visits and medication use^15^. Effective interventions, such as coping effectiveness training^16^ or antidepressant medication, can significantly reduce depressive symptoms following SCI. In particular, personalized interventions in SCI treatment may benefit from an improved understanding of the structure of depressive symptoms associated with SCI, as recovery may depend on the presence of specific symptom patterns. Early identification of potential risk factors for developing such symptoms after SCI is crucial and may help prevent their onset in vulnerable individuals, e.g. through timely therapeutic interventions^17^.

Until now, investigations regarding the association between SCI and depressive symptoms have relied on aggregate measures^18–21^, such as the total BDI score. However, to the best of our knowledge, an in-depth analysis of individual depressive symptoms, which may help to understand psychopathological domains associated with SCI, has not been performed. To this end, we here present a comprehensive investigation of depressive symptoms for a cohort of individuals with SCI from the longitudinal European Multi-Center Study About Spinal Cord Injury (EMSCI; https://www.emsci.org/)^22^, while operationalizing depressive symptoms through the BDI items.

Our work focuses on the following objectives: (1) We provide a detailed characterization of BDI items for individuals with SCI and explore their association with neurological and functional SCI assessments, in particular regarding temporal development. Specifically, we examine whether certain BDI items contribute to the development of a clinically relevant degree of overall depressive symptoms over time, starting from an initially unremarkable status. Conversely, we also explore which BDI items play a key role in the recovery from a clinically relevant degree of overall depressive symptoms during the course of SCI treatment. (2) To check whether there are depressive symptoms that are specific to individuals with SCI, we compare the BDI item profiles of the SCI cohort to those of healthy controls (HCs) and individuals with MDD diagnosis, respectively, of two German reference cohorts. Thereby, we in particular check the suitability and validity of the BDI in SCI settings. (3) We examine to what extent psychiatric, neurological, functional, and demographic characteristics of individuals with SCI, assessed at baseline one month after injury, can predict (i) the occurrence of a clinically relevant degree of overall depressive symptoms, (ii) neurological recovery, and (iii) functional recovery at a follow-up approximately nine months later.

## Methods

### Beck Depression Inventory (BDI)

**Figure 1:**
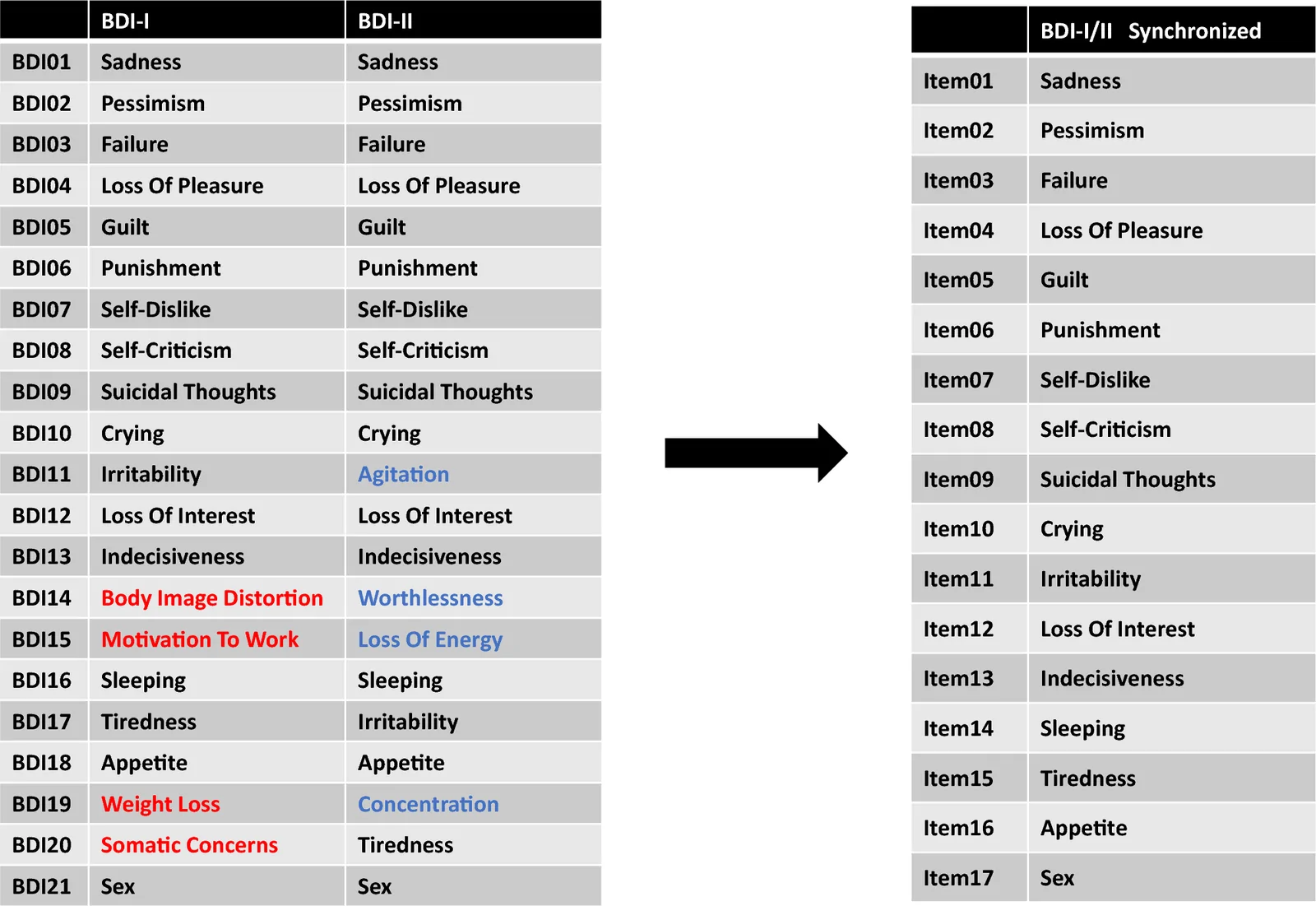
Items of the BDI-I and BDI-II questionnaires, respectively, and their synchronization into BDI-I/II Synchronized: omitting the items marked in color from BDI-I and BDI-II, respectively, leads to BDI-I/II Synchronized.

The Beck Depression Inventory (BDI) is a prominent example of a self-report patient questionnaire to assess the presence and severity of depressive symptoms, which is available in two versions: the original BDI^6^, referred to as BDI-I in what follows, and the revised version BDI-II^7^, respectively. Each version is a 21-item multiple choice inventory, where each item covers a specific aspect related to depression. A complete list of BDI items can be found in Fig. 1, where the items relating to body image distortion, motivation to work, weight loss and somatic concerns, respectively, in the original BDI-I (red) have been replaced by items referring to agitation, worthlessness, loss of energy and concentration, respectively, in the revised BDI-II (blue), in addition to typically slightly modified formulations.

**Figure 2:**
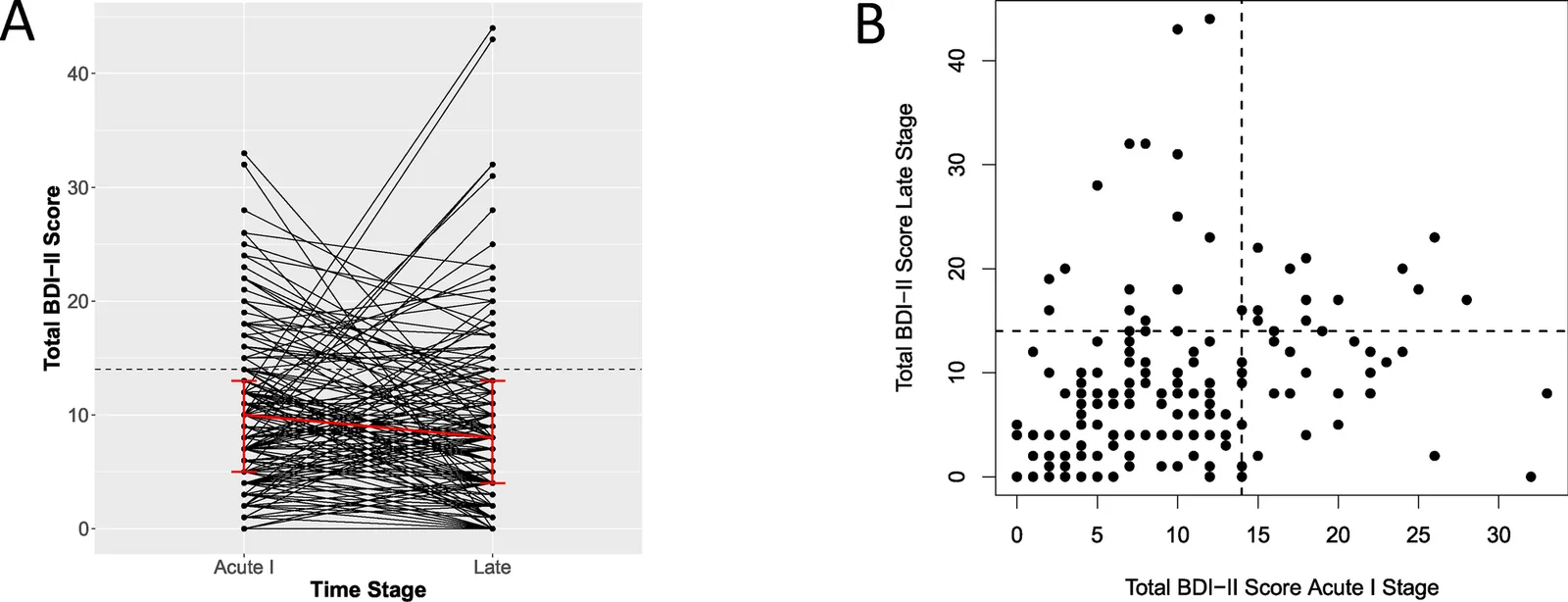
(**A**) Trajectories of the total BDI-II scores (black lines) and trajectory of the mean total BDI-II score (red line; with vertical error bars) and (**B**) scatterplot of the total BDI-II scores, respectively, with respect to the acute I and the late stage for all considered *N=165* individuals with SCI. The horizontal and vertical dashed lines represent the cut-off at 14, relating to a clinically (ir)relevant overall degree of depressive symptoms.

Each BDI item is rated on a 4-point Likert-type scale ranging from 0 to 3. As an overall measure, all the 21 individual BDI item scores are summed up in the total BDI score, ranging from 0 to 63. Specifically for BDI-II, we here used the common cut-off of a total BDI score of 14 to distinguish between a clinically irrelevant (no/minimal) and a clinically relevant (mild/moderate/severe) degree of overall depressive symptoms.

Even though the BDI (item) scores are ordinal in nature, we occasionally treated them as interval-scaled in some of our statistical analyses.

The cohorts considered here used different BDI versions: while the SCI cohort was based on the BDI-II, the FOR2107 and CIMH cohorts used the BDI-I. To enable comparisons across cohorts, we harmonized the two BDI versions by focusing on the 17 common items, referred to as BDI-I/II Synchronized (Fig. 1). For BDI-I/II Synchronized, we derived the total BDI score by summing up the individual BDI scores over the corresponding 17 items, resulting in a theoretical maximum score of 51.

Notably, for the purposes of this study, we operationalized depressive symptoms through the BDI items, even though there is no direct clinical equivalence between depressive symptoms and the individual BDI items.

### SCI cohort

Our analyses were based on data from the European Multi-Center Study About Spinal Cord Injury (EMSCI; https://www.emsci.org/), representing a network that includes around 20 actively participating SCI centers per year. We here specifically considered data from an EMSCI sub-study, comprising contributions from nine of these SCI centers (five in Germany, one in India, one in Italy, and two in Switzerland). This EMSCI subset included 227 individuals with traumatic or ischemic SCI with a date of injury (DOI) between December 2016 and May 2020. The EMSCI is a longitudinal, observational study^4,22^ for which clinical, functional and neurophysiological assessments are aimed to be prospectively collected at five fixed time points over the first year of injury: (1) *łe * 2 weeks after injury (very acute stage), (2) 1 month after injury (acute I stage), (3) 3 months after injury (acute II stage), (4) 6 months after injury (acute III stage), and (5) 12 months after injury (chronic stage). For this study, we combined the acute III and chronic stages into a single so-called late stage, defined as the later of the two stages (acute III or chronic) for which corresponding data were available. Along with the EMSCI assessments, BDI-II questionnaire data^7^ were collected from the individuals with SCI, starting at the acute I stage (the second fixed time point).

We here specifically focused on the subset of those *N=165* individuals with SCI that had complete BDI-II data for both the acute I (baseline) and the late stage, referred to as the SCI cohort in what follows (Table 1).

For the SCI cohort, we considered the following common SCI-specific assessments^23^. Standardized neurological examination was performed according to the International Standards for Neurological Classification of Spinal Cord Injury (ISNCSCI)^24^, which includes the upper extremity motor score (UEMS), lower extremity motor score (LEMS), total light touch (TLT) and total pin prick (TPP) scores^25^ and the resulting neurological level of injury (here split into the three categories cervical, thoracic and lumbar) and injury severity (AIS A through E; American Spinal Injury Association (ASIA) impairment scale (AIS)^26^). Functional assessment was performed using the spinal cord independence measure version III (SCIM-III)^27^), covering three subscales referring to self-care, respiration and sphincter management (RSM), and mobility, respectively. An overview of the SCI-specific assessments can be found in Supplementary Table S1, and respective basic statistics regarding the SCI cohort are given in Table 1.

**Table 1:** Summary of the demographic and SCI-specific information of the *N=165*-member cohort of individuals with SCI at the acute I and late stage, respectively. *p*-values regarding comparisons between acute I and late stage are based on Bowker test (AIS) and paired Wilcoxon tests (UEMS, LEMS, TLT, TPP, SCIM-III-III subscales), respectively.

	Acute I	Late	*p*-value
	*(N=165)*	*(N=165)*	
DEMOGRAPHICS			
Gender			
Male	125 (75.8%)		
Female	40 (24.2%)		
Age at DOI, yr			
Mean (SD)	47.0 (19.1)		
Median (Min-Max)	48 (16-86)		
SCI-BASIC CHARACTERISTICS			
Cause of Injury			
Traumatic	154 (93.3%)		
Ischemic	11 (6.7%)		
Injury Level			
Cervical	90 (54.5%)		
Thoracic	54 (32.7%)		
Lumbar	21 (12.7%)		
SCI-NEUROLOGICAL ASSESSMENTS			
AIS			
A	59 (35.8%)	47 (28.5%)	
B	21 (12.7%)	13 (7.9%)	
C	22 (13.3%)	21 (12.7%)	$1.6 \times 10^{-5}$
D	52 (31.5%)	72 (43.6%)	
E	0 (0%)	1 (0.6%)	
Missing	11 (6.7%)	11 (6.7%)	
UEMS			
Mean (SD)	36.8 (16.4)	41.8 (13.4)	
Median (Min-Max)	44 (0-50)	50 (0-50)	$2.3 \times 10^{-14}$
Missing	11 (6.7%)	10 (6.1%)	
LEMS			
Mean (SD)	16.4 (19.8)	23.4 (21.2)	
Median (Min-Max)	3 (0-50)	22.5 (0-50)	$1.9 \times 10^{-14}$
Missing	11 (6.7%)	11 (6.7%)	
TLT			
Mean (SD)	71.8 (28.0)	76.5 (26.1)	
Median (Min-Max)	78 (1-112)	77.5 (14-112)	$7.0 \times 10^{-4}$
Missing	16 (9.7%)	11 (6.7%)	
TPP			
Mean (SD)	59.9 (31.1)	64.4 (29.2)	
Median (Min-Max)	60 (1-112)	64 (10-112)	0.0103
Missing	13 (7.9%)	11 (6.7%)	
SCI-FUNCTIONAL ASSESSMENTS			
SCIM-III Self-Care			
Mean (SD)	6.9 (6.2)	13.2 (6.8)	
Median (Min-Max)	6 (0-20)	16 (0-20)	$< 2.2 \times 10^{-16}$
Missing	1 (0.6%)	5 (3.0%)	
SCIM-III RSM			
Mean (SD)	14.7 (10.6)	26.8 (11.6)	
Median (Min-Max)	10.5 (0-40)	30.5 (0-40)	$< 2.2 \times 10^{-16}$
Missing	1 (0.6%)	5 (3.0%)	
SCIM-III Mobility			
Mean (SD)	6.5 (9.4)	19.0 (13.8)	
Median (Min-Max)	3 (0-40)	17 (0-40)	$< 2.2 \times 10^{-16}$
Missing	1 (0.6%)	5 (3.0%)	

Here, neurological recovery was defined as an improvement from the acute I to the late stage in terms of AIS, UEMS, and TLT, respectively. Analogously, functional recovery was defined as an improvement with respect to the SCIM-III subscales. An AIS improvement was defined as an improvement of at least one AIS grade. Improvements in UEMS, TLT, or the SCIM-III subscales were defined as a corresponding normalized percentage increase of at least *25%* (Supplementary Material, Section S1).

### Cohorts for comparison to the SCI cohort

To check whether there are depressive symptoms that are specific to individuals with SCI, we compared the BDI item profiles from the *N=165* SCI individuals at the late stage to those of HCs and individuals with an MDD diagnosis, respectively, from two reference cohorts assembled by (1) the FOR2107 consortium^28^ (referred to as the FOR2107 cohort) and (2) the Central Institute of Mental Health^29^ (referred to as the CIMH cohort), respectively. As the cohorts were based on different BDI versions, we here employed the 17-item BDI-I/II Synchronized for comparison.

The data from the FOR2107 cohort were collected in German psychiatric hospitals in Marburg and Münster, respectively, between 2014 and 2019 as part of the longitudinal FOR2107 study^28^, gathering data from a baseline time point and a follow-up time point after two years. To allow for a consistent comparison to the late stage focused on in the SCI cohort, we here considered the follow-up time point in the FOR2107 study. The FOR2107 data consisted of 614 HCs and 134 individuals diagnosed with MDD (based on DSM-5).

The data from the CIMH cohort were collected at the Central Institute of Mental Health in Mannheim, Germany, as part of the IntegraMent study^29^. It comprised 181 HCs and 43 individuals with MDD diagnosis (based on DSM-IV).

### Statistical analyses and prediction models

All statistical analyses, including the prediction modeling, were performed using the R language and environment for statistical computing^30^, version 4.4.1.

#### Basic analyses

For basic analyses or comparisons of cohorts, we employed standard techniques such as correlation analyses, Fisher’s exact tests, or variants of Wilcoxon rank sum tests (with possible Bonferroni correction for multiple testing), as indicated along with the respective results.

To generate an overview of BDI item profiles across individuals, we employed clustered heatmaps with respective dendrograms using the R package pheatmap^31^. Such maps aim at allowing for the identification of similar BDI items (with respect to the individuals) and similar individuals (with respect to the BDI item profiles), using complete linkage clustering based on the Euclidean distance here. The level of statistical significance was set to *5%*.

To assess relationships between BDI-II items and SCI-specific neurological and functional assessments, we also derived factor models using exploratory factor analysis (Supplementary Material, Section S2.3).

#### Principal component analysis

To compare the BDI item profiles of the SCI cohort to those of HCs and individuals with MDD diagnosis, respectively, in the FOR2107 and CIMH reference cohorts, respectively, we used standard principal component analyses (PCA)^32^ as implemented in the R package FactoMineR^33^. Results are visualized in PCA biplots^34^, using the R package factoextra^35^. To enable proper comparison across cohorts, original BDI item data were adjusted for age and gender by performing PCA on the residuals from linear models that regressed out these variables.

#### Prediction models

We considered random forest (RF) classification approaches^36^, as implemented in the R package randomForest^37^, to assess whether features from the acute I stage (baseline) could predict (i) the occurrence of a clinically relevant degree of overall depressive symptoms at late stage, (ii) neurological recovery, and (iii) functional recovery in individuals with SCI. The specific outcomes were binary variables indicating whether an SCI individual (i) had a total BDI-II score *\ge 14* at late stage, (ii) improved in terms of the AIS, UEMS and TLT, respectively, from the acute I to the late stage, and (iii) improved in terms of the SCIM-III subscales from the acute I to the late stage. In total, we included 33 features from the acute I stage comprising neurological (InjuryLevel, UEMS, LEMS, TLT, TPP, AIS) and functional (SCIM-III subscales on self-care, RSM, and mobility) SCI-specific parameters, psychiatric variables (the 21 BDI-II items, and a binary variable, BinTotalBDI, indicating whether an SCI individual had a total BDI-II score *\ge 14* at the acute I stage or not) and demographic information (gender, age). In each setting, we included only SCI individuals with complete data for all involved variables (Supplementary Material, Section S5.1; Supplementary Table S5).

For model assessment, we performed repeated nested cross-validation and computed the respective distributions for the following evaluation measures: area under the receiver-operating characteristic (AUROC), area under the precision-recall curve (AUPRC) and Matthews correlation coefficient (MCC)^38^.

For each outcome, we derived the top *25%* (i.e., 8) of the features with the highest variable importance from the respective RF model. Here, variable importance was quantified by the median value of the mean decrease in accuracy from the respective distributions.

Details on the implementation and evaluation of the prediction models are deferred to the Supplementary Material, Section S5.1.

## Results

### SCI cohort

The *N=165*-member SCI cohort comprised 125 (*75.8%*) males and 40 (*24.2%*) females, where the overall mean age referring to the DOI was 47.0 (SD: 19.1) years. Thus, it was reasonably representative regarding gender ratio and age structure^4,39^. In 154 (93.3*%*) people the cause of injury was traumatic, and in 11 (6.7*%*) it was ischemic. Furthermore, 90 (54.5*%*) individuals had a cervical, 54 (32.7*%*) a thoracic and 21 (12.7*%*) a lumbar injury level (Table 1). The time span between the acute I and late (i.e., acute III or chronic stage) stage BDI assessments was on average 271.5 days (median: 287 days; SD: 118.8 days).

Both at the acute I and the late stage, mean, median and mode of the total BDI-II score were at a clinically irrelevant level (*<14*) for overall depressive symptoms (Table 2; Fig. 2). Overall, the total BDI-II scores were significantly higher at acute I stage than at late stage (paired Wilcoxon rank sum test: *p=0.0034*), while there was a positive longitudinal relationship between these scores (Fig. 2; Spearman’s rho *ρ =0.35*, $p=3.5 \times 10^{-6}$). However, there was no statistically significant difference between the binarized total BDI-II scores (cut-off at 14) at the two time stages (McNemar test: *p=0.451*), and a positive longitudinal correlation ($\operatorname {MCC} = 0.25$, *p=0.0012*). In particular, out of 165 individuals with SCI, 41 (24.8%) showed a clinically relevant degree of overall depressive symptoms according to the BDI-II at the acute I stage, 35 (21.2%) at the late stage, and 16 (9.7%) at both stages (Fig. 2).

**Table 2:** Median together with first quartile (Q1) and third quartile (Q3), mean together with standard deviation (SD), and mode together with minimum (Min) and maximum (Max) regarding the BDI-II item scores and the total BDI-II score for the *N=165*-member SCI cohort at the acute I and the late stage, respectively.

	Acute I			Late		
	Median	Mean	Mode	Median	Mean	Mode
	(Q1-Q3)	(SD)	(Min-Max)	(Q1-Q3)	(SD)	(Min-Max)
BDI01 Sadness	1 (0-1)	0.58 (0.60)	0 (0-2)	0 (0-1)	0.38 (0.51)	0 (0-2)
BDI02 Pessimism	0 (0-1)	0.48 (0.60)	0 (0-3)	0 (0-1)	0.36 (0.60)	0 (0-3)
BDI03 Failure	0 (0-0)	0.18 (0.47)	0 (0-2)	0 (0-0)	0.18 (0.47)	0 (0-2)
BDI04 Loss Of Pleasure	1 (0-1)	0.72 (0.58)	1 (0-2)	1 (0-1)	0.76 (0.77)	1 (0-3)
BDI05 Guilt	0 (0-0)	0.27 (0.60)	0 (0-3)	0 (0-0)	0.21 (0.53)	0 (0-3)
BDI06 Punishment	0 (0-0)	0.20 (0.57)	0 (0-3)	0 (0-0)	0.22 (0.60)	0 (0-3)
BDI07 Self-Dislike	0 (0-0)	0.16 (0.43)	0 (0-2)	0 (0-0)	0.22 (0.46)	0 (0-2)
BDI08 Self-Criticism	0 (0-1)	0.51 (0.60)	0 (0-3)	0 (0-1)	0.45 (0.60)	0 (0-3)
BDI09 Suicide	0 (0-0)	0.10 (0.30)	0 (0-1)	0 (0-0)	0.17 (0.44)	0 (0-3)
BDI10 Crying	0 (0-1)	0.47 (0.66)	0 (0-3)	0 (0-1)	0.36 (0.59)	0 (0-3)
BDI11 Agitation	0 (0-1)	0.42 (0.59)	0 (0-3)	0 (0-1)	0.33 (0.56)	0 (0-3)
BDI12 Loss Of Interest	0 (0-0)	0.23 (0.51)	0 (0-3)	0 (0-0)	0.28 (0.56)	0 (0-3)
BDI13 Indecisiveness	0 (0-1)	0.45 (0.60)	0 (0-3)	0 (0-1)	0.43 (0.59)	0 (0-2)
BDI14 Worthlessness	0 (0-1)	0.47 (0.57)	0 (0-3)	0 (0-1)	0.42 (0.61)	0 (0-3)
BDI15 Loss Of Energy	1 (0-1)	0.83 (0.65)	1 (0-3)	1 (0-1)	0.77 (0.59)	1 (0-3)
BDI16 Sleeping	1 (0-1)	0.92 (0.76)	1 (0-3)	1 (0-1)	0.73 (0.77)	1 (0-3)
BDI17 Irritability	0 (0-0)	0.24 (0.48)	0 (0-3)	0 (0-1)	0.35 (0.57)	0 (0-3)
BDI18 Appetite	1 (0-1)	0.85 (0.89)	1 (0-3)	0 (0-1)	0.55 (0.72)	0 (0-3)
BDI19 Concentration	0 (0-1)	0.35 (0.57)	0 (0-3)	0 (0-1)	0.47 (0.66)	0 (0-3)
BDI20 Tiredness	1 (1-1)	0.92 (0.63)	1 (0-3)	1 (0-1)	0.79 (0.60)	1 (0-3)
BDI21 Sex	0 (0-1)	0.80 (1.01)	0 (0-3)	0 (0-1)	0.68 (0.97)	0 (0-3)
Total BDI-II Score	10 (5-13)	10.13 (6.59)	7 (0-33)	8 (4-13)	9.10 (7.75)	8 (0-44)

**Figure 3:**
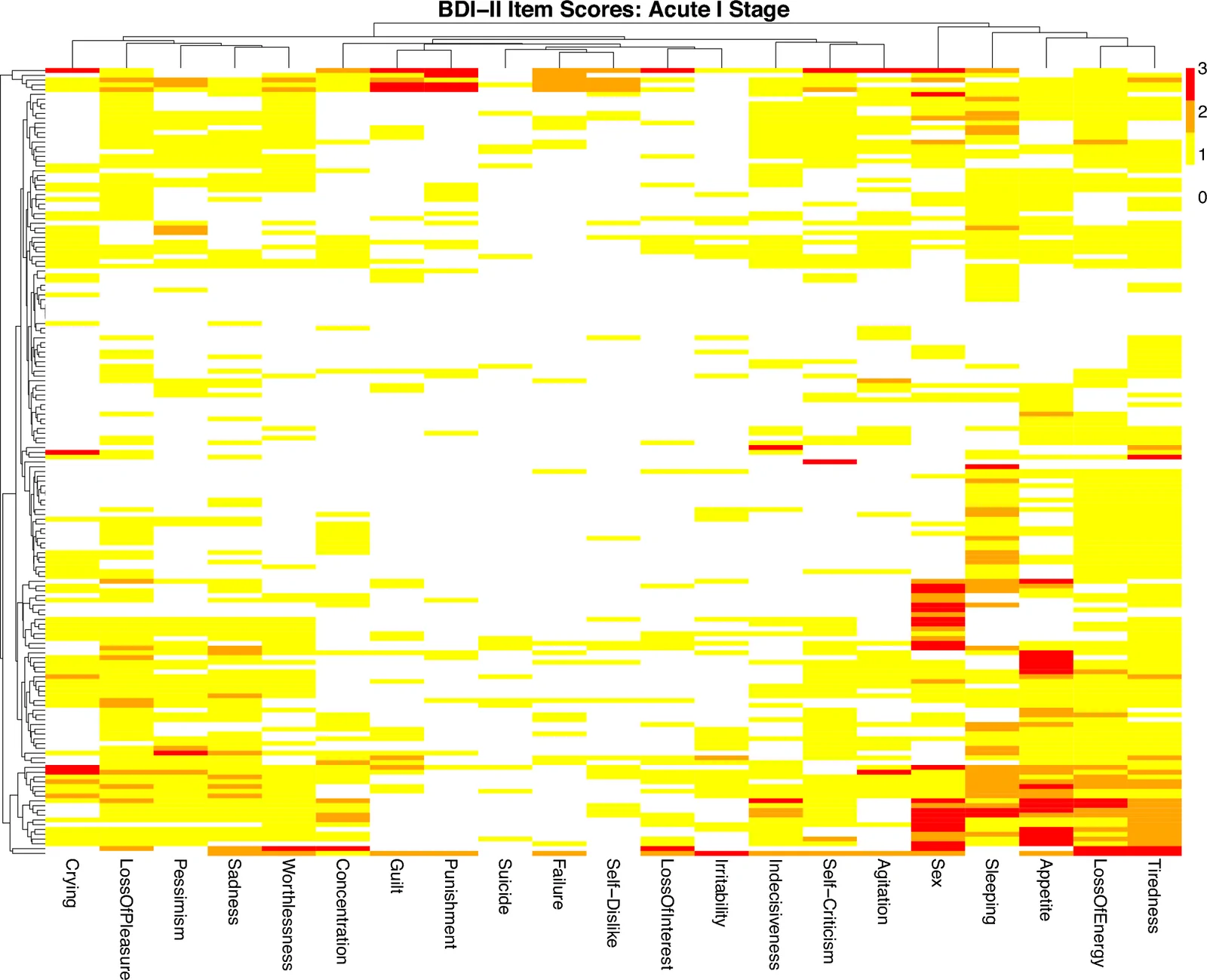
Heatmap representation for the BDI-II item scores (attaining values of either 0 (white), 1 (yellow), 2 (orange) or 3 (red)) of the *N=165*-member cohort of individuals with SCI at the acute I stage. Rows correspond to individuals with SCI, and columns to BDI-II items. Both individuals with SCI and BDI-II items are arranged based on a hierarchical clustering as indicated by the respective dendrograms.

**Figure 4:**
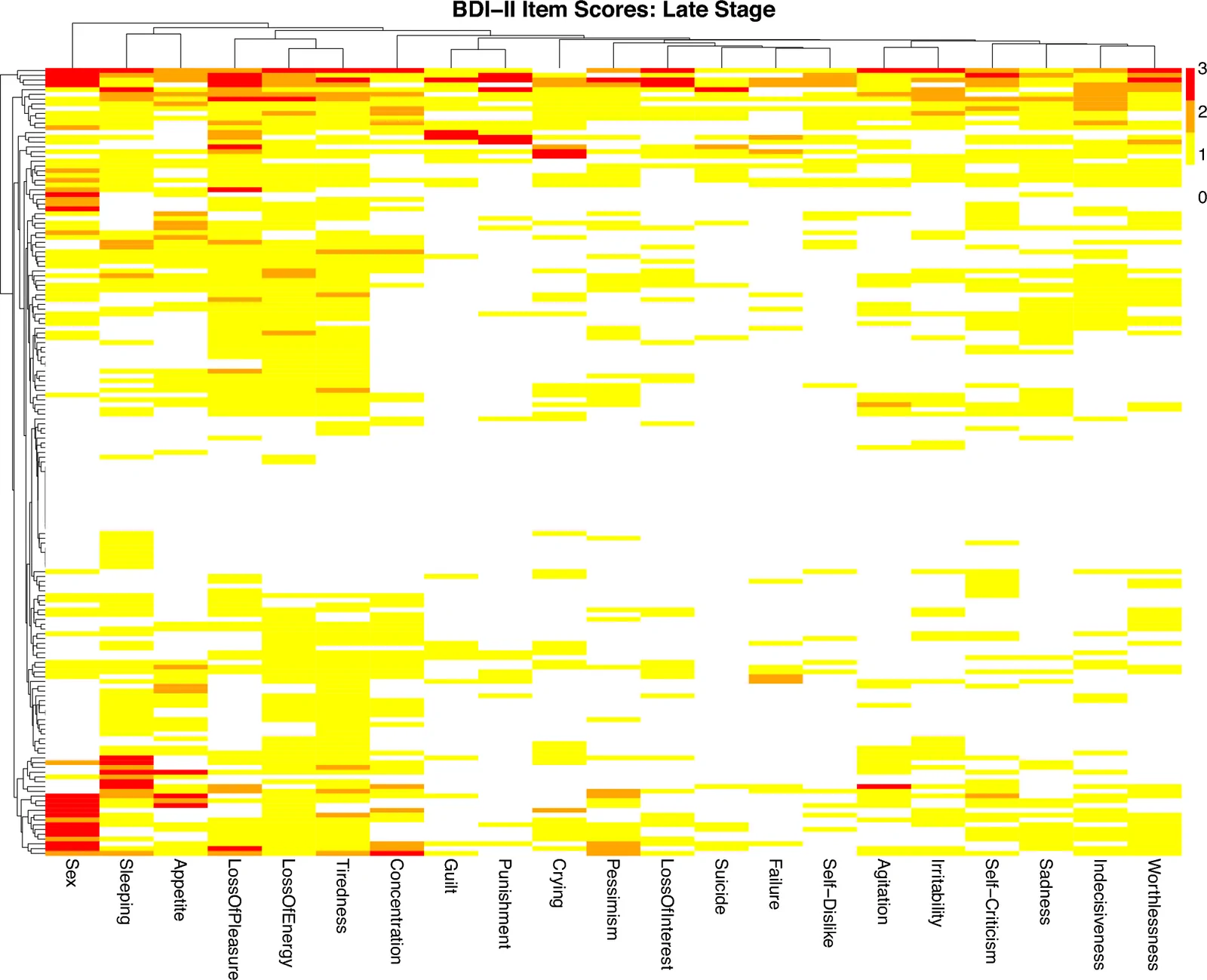
Heatmap representation for the BDI-II item scores (attaining values of either 0 (white), 1 (yellow), 2 (orange) or 3 (red)) of the *N=165*-member cohort of individuals with SCI at the late stage. Rows correspond to individuals with SCI, and columns to BDI-II items. Both individuals with SCI and BDI-II items are arranged based on a hierarchical clustering as indicated by the respective dendrograms.

At both time stages, non-zero scores could be observed mostly for BDI-II items related to somatic phenomena: tiredness, sleeping, appetite, loss of energy, sex and loss of pleasure (Table 2; Figures 3 and 4). That is, these items mainly drove high total BDI-II scores. In contrast, items related to a negative self-attitude, such as suicidal thoughts, self-dislike, failure and punishment, showed BDI-II scores of zero for the vast majority of SCI individuals (Table 2; Figs. 3 and 4). While there were some changes in the scores for the BDI-II items from the acute I to the late stage, significant decreases after Bonferroni correction could eventually be confirmed for the items appetite and sadness only (Table 2; Supplementary Material, Section S2.1; Supplementary Figure S1).

Prominent Spearman correlations among BDI-II items existed e.g. between tiredness and loss of energy, indecisiveness and worthlessness (both time stages), sadness and pessimism, sadness and loss of pleasure (acute I stage), guilt and punishment, and pessimism and suicide (late stage) (Supplementary Material, Section 2.2; Supplementary Figures S2 to S5).

At both time stages, neurological and functional SCI-specific parameters were typically pronouncedly positively correlated (Supplementary Figures S2 to S5). BDI-II items showed negative correlations with these parameters, with significant associations occurring more often for functional than for neurological variables (Supplementary Figures S2 and S4). However, after Bonferroni adjustment, only a few correlations remained significant: at the acute I stage, the correlations between SCIM-III mobility and sadness, SCIM-III RSM and sadness, SCIM-III RSM and loss of energy, and SCIM-III RSM and tiredness; and at the late stage, the correlations between SCIM-III RSM and pessimism, and SCIM-III self-care and pessimism (Supplementary Figures S3 and S5).

For the acute I stage, BDI-II items and SCI-specific neurological and functional parameters in SCI individuals were adequately captured by an eight-factor model (Supplementary Material, Section S2.3; Supplementary Table S2). There was a clear separation between BDI-II items (five factors) and SCI parameters (three factors). Notably, somatic depressive symptoms formed a separate factor, self-blame emerged as a distinct dimension, and anhedonia (a key symptom for MDD diagnosis) stood on its own. Among the SCI-specific parameters, neurological measures and the functional measures from the SCIM-III subscales clearly loaded onto separate factors.

Analogously, for the late stage, an eight-factor model was appropriate, again revealing a separation between BDI-II items (four factors) and SCI-specific variables (three factors) (Supplementary Material, Section S2.3; Supplementary Table S3). For the BDI-II items, the factors corresponded to mood/core depressive symptoms, guilt/moral distress, neurobehavioral symptoms, and pessimism. Regarding the SCI-specific parameters, neurological measures and the functional measures from the SCIM-III subscales basically loaded onto separate factors.

#### BDI-II-specific subgroups of the SCI cohort

Based on the temporal development of the total BDI-II scores from the acute I to the late stage (Fig. 2), we specifically considered the following clinically relevant subgroups of the *N=165*-member SCI cohort for detailed analyses (Supplementary Material, Section S3):

- *Subgroup A (“Worsened”)*: 19 individuals with SCI with a total BDI-II score *< 14* at acute I stage, but a total BDI-II score *\ge 14* at late stage (i.e., individuals with SCI that developed a clinically relevant degree of overall depressive symptoms over time), and
- *Subgroup B (“Recovered”)*: 25 individuals with SCI with a total BDI-II score *\ge 14* at acute I stage, but a total BDI-II score *< 14* at late stage (i.e., individuals with SCI that recovered from a clinically relevant degree of overall depressive symptoms over time).

In Subgroup A, for the driving, physical-somatic BDI-II items, already high scores at the acute I stage stayed high (tiredness, sex) or even significantly increased (loss of pleasure, loss of energy, sleeping) at the late stage (paired Wilcoxon rank sum tests with Bonferroni adjustment). Additionally, the scores of other items (that are no driving items at the acute I stage) significantly increased: this held for loss of interest (relating to anhedonia as a key symptom for MDD diagnosis), indecisiveness, agitation, suicide, irritability, self-dislike and guilt, i.e., items relating to non-somatic phenomena (Supplementary Material, Section S3.1; Supplementary Figure S6).

In Subgroup B, in particular the scores of the driving BDI-II items sex, appetite, tiredness, sleeping, loss of energy and loss of pleasure significantly decreased from the acute I to the late stage, where the scores of the other items stayed at low levels (paired Wilcoxon rank sum tests with Bonferroni adjustment). Hence, for the SCI individuals in Subgroup B, predominantly physical-somatic items were pronounced at the acute I stage, but improved by the late stage, leading to an improvement of the degree of overall depressive symptoms to a clinically irrelevant level (Supplementary Material, Section S3.2; Supplementary Figure S7).

Overall, in the total *N=165*-member SCI cohort, Fisher’s exact tests indicated that the development of, or recovery from, a clinically relevant degree of overall depressive symptoms from the acute I to the late stage (i.e., being in Subgroup A or B) appeared to be not associated with neurological or functional recovery (Supplementary Material, Sections S3.1 and S3.2).

### Comparison of SCI cohort to FOR2107 and CIMH reference cohorts with respect to BDI-I/II Synchronized

HCs in the FOR2107 cohort had an average age of 37.5 years (SD: 13.3), whereas HCs in the CIMH cohort had an average age of 31.2 years (SD: 11.4). While the proportion of female HCs was higher in the FOR2107 cohort (*64.8%*), it was lower in the CIMH cohort (*46.4%*) (Supplementary Table S4).

Among individuals with MDD diagnosis, age structure was comparable in the FOR2107 (mean (SD): 39.6 (14.0) years) and CIMH cohorts (mean (SD): 36.8 (11.7) years). The same held for gender ratio (FOR2107: *70.9%* females; CIMH: *74.4%* females). Compared to the SCI cohort, individuals with MDD diagnosis in the reference cohorts were generally younger and had a higher proportion of females (Supplementary Table S4).

The SCI cohort had a median total BDI-I/II Synchronized score of 6, where the highest item scores were obtained for tiredness, loss of pleasure, sleeping, sex, and appetite, i.e., items relating to physical-somatic phemomena (Supplementary Material, Section S4.1; Supplementary Table S4; Supplementary Figure S8).

The FOR2107 and CIMH reference cohorts were very similar regarding total BDI-I/II Synchronized scores (Supplementary Table S4), both for HCs (FOR2107 and CIMH: median of 1; Wilcoxon rank sum test: *p=0.49*) and for individuals with MDD diagnosis (FOR2107: median of 16; CIMH: median of 17; Wilcoxon rank sum test: *p=0.44*).

**Figure 5:**
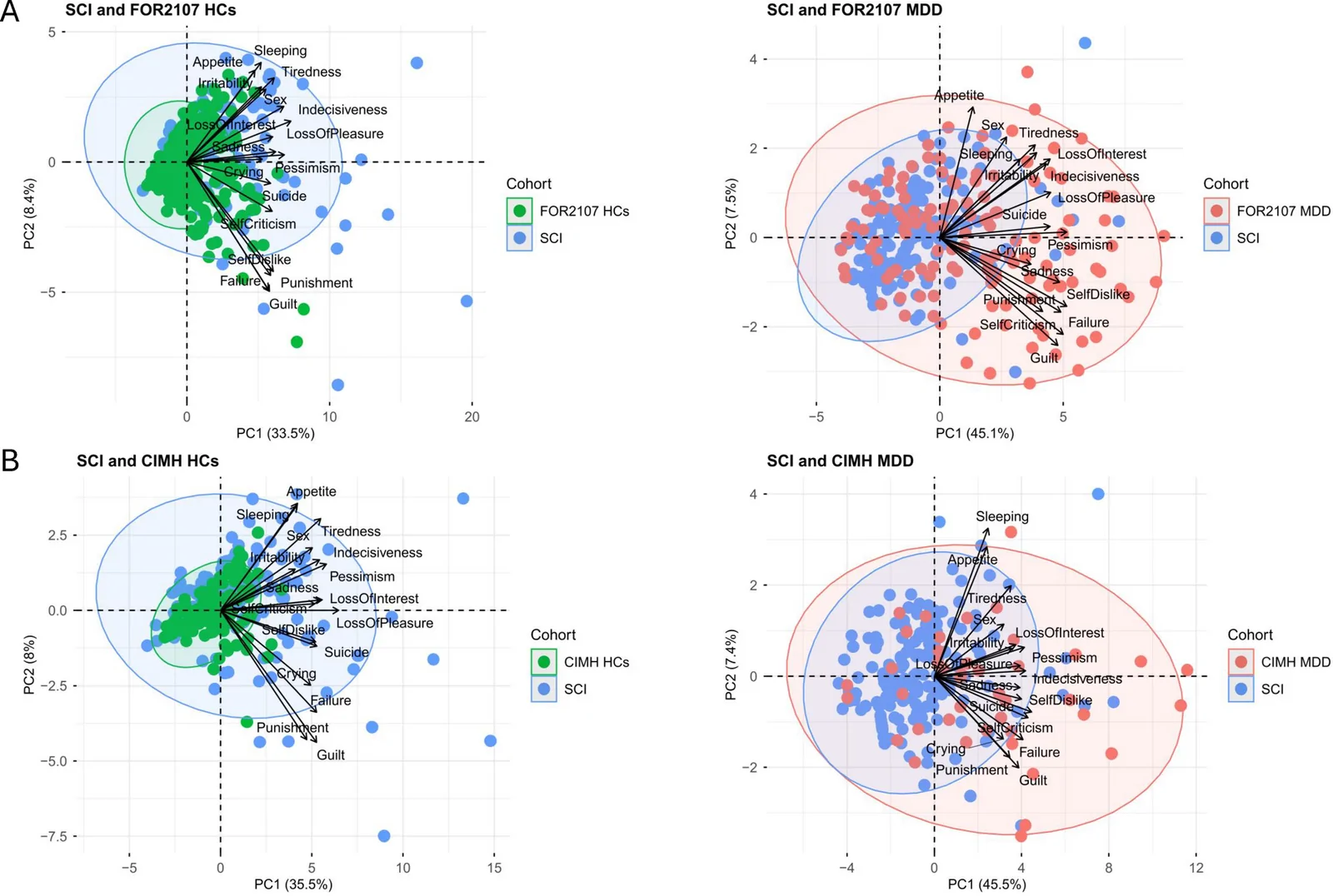
PCA biplots to compare BDI item profiles of the individuals with SCI to those of HCs (left) and individuals with MDD diagnosis (right) from the (**A**) FOR2107 cohort and (**B**) CIMH cohort, respectively. Here, BDI refers to BDI-I/II Synchronized, and the underlying data was adjusted for age and gender.

In both the FOR2107 and the CIMH cohorts, HCs typically showed low total BDI-I/II Synchronized scores, with only very few non-zero item scores (Supplementary Table S4). Consequently, HCs clustered rather tightly together around the origin in the corresponding PCA biplots (left plots in Fig. 5).

In contrast, individuals with MDD diagnosis showed far more non-zero scores for a broad range of BDI-I/II Synchronized items (Supplementary Material, Section S4.1; Supplementary Figures S9 and S10), demonstrating the heterogeneity of depression and illustrated in greater spread in the PCA biplots (right plots in Fig. 5).

Total BDI-I/II Synchronized scores (reflecting the degree of overall depressive symptoms) in individuals with SCI were signifcantly higher than those in HCs (Wilcoxon rank sum tests: SCI vs. FOR2107 HCs and SCI vs. CIMH HCs: $p<2.2 \times 10^{-16}$), but lower than those in individuals with MDD diagnosis (Wilcoxon rank sum tests: SCI vs. FOR2107 MDD: $p<2.2 \times 10^{-16}$; SCI vs. CIMH MDD: $p=1.5 \times 10^{-11}$).

For both the FOR2107 and the CIMH individuals with MDD diagnosis, the individual BDI-I/II Synchronized item scores were significantly greater than those for the individuals with SCI for the vast majority of items. Most obvious differences were observed for items relating to self-rejection, such as self-dislike, failure, or guilt (Bonferroni-adjusted Wilcoxon rank sum tests; Supplementary Material, Sections S4.2 and S4.3)

When considering individuals with high total BDI-I/II Synchronized scores (corresponding to the points far away from the origin in the plots in Fig. 5), individuals with SCI exhibited distinct BDI item profiles than individuals with MDD diagnosis. High total BDI-I/II Synchronized scores in individuals with SCI were primarily driven by items related to somatic-physical phenomena, such as sex, tiredness, sleeping, and appetite (upper right quadrants in the right plots in Fig. 5). In contrast, those somatic items only partly drove high total BDI-I/II Synchronized scores in individuals diagnosed with MDD. These were in fact driven by a broader range of items, particularly those related to self-rejection, such as self-dislike, self-criticism, or failure (lower right quadrants in the right plots in Fig. 5).

While key observations regarding the comparison of the cohorts are summarized above, detailed results are deferred to the Supplementary Material, Section S4.

### Prediction models

#### Predicting the occurrence of a clinically relevant degree of overall depressive symptoms at late stage

For the prediction of the occurrence of a clinically relevant degree of overall depressive symptoms at late stage based on feature data from the acute I stage, the RF model showed a mediocre predictive performance (e.g., median AUROC of 0.60, *p=0.01*; Table 3). The most important features comprised neurological (UEMS, TPP, and TLT), functional (SCIM-III self-care), and psychiatric (BDI-II items worthlessness, tiredness, and indecisiveness; and BinTotalBDI), but no demographic variables (Supplementary Material, Section S5.2; Supplementary Figures S11 and S12).

**Table 3:** RF prediction models based on features from acute I stage for the outcomes of the occurrence of a clinically relevant degree of overall depressive symptoms at late stage, neurological recovery (in terms of AIS, UEMS and TLT), and functional recovery (in terms of the SCIM-III subscales): top 25*%* of features with the highest variable importances, and median AUROC, AUPRC, and MCC values with corresponding *p*-values from permutation tests. For evaluation, 10 times repeated 5-fold stratified cross validation was used, and the reported distributions of the evaluation measures are based on the 10 repetitions for the median values over the 5 folds.

Outcome	Top *25%* of Features with the Highest Variable Importances	Median	Median	Median
		AUROC	AUPRC	MCC
Occurrence of a Clinically Relevant	UEMS, TPP, TLT, SCIM-III Self-Care,	0.60	0.22	0.27
Degree of Overall Depressive	Worthlessness, Tiredness,	*(p=0.01)*	*(p=0.29)*	*(p=0.17)*
Symptoms at Late Stage	BinTotalBDI, Indecisiveness			
Neurological Recovery	AIS, LEMS, SCIM-III Mobility,	0.82	0.52	0.58
wrt AIS	TLT, UEMS, SCIM-III RSM	*(p<0.01)*	*(p<0.01)*	*(p<0.01)*
	SCIM-III Self-Care, Sex			
Neurological Recovery	UEMS, InjuryLevel, AIS,	0.93	0.74	0.85
wrt UEMS	LEMS, TPP, SCIM-III Self-Care,	*(p<0.01)*	*(p<0.01)*	*(p<0.01)*
	TLT, Agitation			
Neurological Recovery	LEMS, TPP, InjuryLevel,	0.67	0.36	0.36
wrt TLT	SCIM-III RSM, AIS, TLT,	*(p<0.01)*	*(p<0.01)*	*(p<0.01)*
	UEMS, Sex			
Functional Recovery	UEMS, SCIM-III Self-Care, TPP,	0.87	0.88	0.61
wrt SCIM-III Self-Care	SCIM-III RSM, SCIM-III Mobility,	*(p<0.01)*	*(p<0.01)*	*(p<0.01)*
	TLT, Age, BinTotalBDI			
Functional Recovery	Age, SCIM-III RSM, UEMS,	0.85	0.85	0.65
wrt SCIM-III RSM	SCIM-III Self-Care, TLT, SCIM-III Mobility,	*(p<0.01)*	*(p<0.01)*	*(p<0.01)*
	TPP, Irritability			
Functional Recovery	Age, UEMS, LEMS, TPP,	0.86	0.84	0.66
wrt SCIM-III Mobility	AIS, SCIM-III Mobility,	*(p<0.01)*	*(p<0.01)*	*(p<0.01)*
	TLT, SCIM-III Self-Care			

#### Predicting neurological recovery

Regarding the prediction of neurological recovery, the performances of the RF models were typically good (Table 3; Supplementary Material, Section S5.3; Supplementary Figures S13, S15, and S17). Exemplarily, the RF model for neurological recovery with respect to AIS had a median AUROC of 0.82 (*p<0.01*), while that with respect to UEMS had a median AUROC of 0.93 (*p<0.01*) (Table 3). Model performances were driven primarily by the extent of neurological and functional impairment at the acute I stage, with demographic and psychiatric features contributing far less (Supplementary Material, Section S5.3; Supplementary Figures S14, S16, and S18).

#### Predicting functional recovery

In terms of the prediction of functional recovery with respect to the SCIM-III subscales, the RF models performed well (Table 3; Supplementary Material, Section S5.4; Supplementary Figures S19, S21, and S23). For instance, the RF model for functional recovery with respect to the SCIM-III subscale referring to mobility had a median AUROC of 0.86 (*p<0.01*) (Table 3). Model performance was largely determined by the degree of neurological and functional impairment at the acute I stage, as well as age, and virtually not by psychiatric parameters (Supplementary Material, Section S5.4; Supplementary Figures S20, S22, and S24).

## Discussion

Here, we conducted a pioneering study on the detailed characterization of depressive symptoms in individuals with SCI. Our SCI cohort, representative with respect to average age at injury and gender (more males)^39^, consisted of a sample from the EMSCI, a prospective observational study with longitudinal data obtained 4 weeks (acute I stage) and 6–12 months (late stage) after the injury, respectively. As our focus was on evaluating the development of depressive symptoms in SCI, we included only participants with complete BDI-II data at both the acute I and late stages. This reduced the SCI cohort from 227 to *N=165* individuals. To assess the potential impact of attrition on our findings, we compared baseline (i.e., acute I) psychiatric, demographic, neurological, and functional characteristics between SCI individuals who were excluded from the final analyses ($N_{\operatorname {excl}} = 62$) and those who were included (*N = 165*). These comparisons did not reveal statistically significant differences in any of the examined variables (Supplementary Material, Section S6; Supplementary Table S6). Although the absence of detectable baseline differences cannot rule out all forms of selection bias, it suggests that participant exclusion was unlikely to have introduced substantial systematic selection effects or biases, thereby supporting the representativeness of the analyzed SCI cohort.

Depressive symptoms in SCI individuals were operationalized through BDI items and compared to those of HCs and individuals with MDD diagnosis from two German reference cohorts (FOR 2107 and CIMH). In particular, for individuals with MDD diagnosis^5^, both reference cohorts were typical in terms of age structure (on average younger than individuals with SCI) and gender ratio (more females; reversed compared to the SCI cohort), and were also comparable to the BDI-I calibration sample^6^ with respect to these parameters.

For cohort comparisons, we adjusted for age and gender and applied a standard PCA based on Pearson correlations. This approach does not fully account for the ordinal nature of the BDI data and instead assumes interval-level measurement, an assumption that may not be strictly met. However, alternative PCA analyses based on polychoric correlations yielded highly similar results (Supplementary Material, Section S7; Supplementary Figure S25), suggesting that our findings are robust to the choice of correlation measure and PCA implementation.

Overall, 25% of SCI individuals had a total BDI-II score *\ge 14* (indicating a clinically relevant degree of overall depressive symptoms) at the acute I stage, and 21% at the late stage. Although no MDD diagnoses were available for the SCI cohort, these values align with a meta-analysis by Williams and Murray^13^ on the prevalence of an MDD diagnosis after SCI (according to DSM-III/RDC or DSM-IV), which reported a mean prevalence estimate of 22.2%, with a lower-bound estimate of 18.7% and an upper bound estimate of 26.3%.

However, and importantly, at an individual item level, high BDI-II scores for individuals with SCI were primarily related to physical-somatic phenomena, such as problems with respect to sleep, tiredness, appetite, and libido.

Total BDI scores in individuals with SCI were significantly higher than those in HCs, but significantly smaller than those in individuals with MDD diagnosis. Individuals with MDD diagnosis showed a broad range of items contributing to high total BDI scores, reflecting the high heterogeneity of depression. These items also included physical-somatic phenomena, but were primarily related to self-blame and self-hatred, such as pronounced self-dislike or self-criticism, or feelings of guilt or failure. Overall, individuals with SCI thus showed distinct BDI item profiles compared to individuals with MDD diagnosis.

As reflected by the total BDI-II scores, more individuals with SCI showed improvement rather than deterioration from the acute I to the late stage in terms of clinically relevant degrees of overall depressive symptoms. Improvements were predominantly driven by decreases in BDI-II scores for items related to physical-somatic issues. These symptoms may be pronounced shortly after the injury as a direct consequence of the SCI itself, but tend to diminish over time.

In contrast, the development of a clinically relevant degree of overall depressive symptoms from the acute I to the late stage in individuals with SCI was primarily reflected in increased BDI-II scores for items related to self-hatred, anhedonia, emotional overload, or cognitive stress. In the early stages of treatment, individuals with SCI may still retain hope for improvement and successful adaptation to their circumstances. Furthermore, a sense of community in the acute I clinical in-hospital setting (where the BDI-II assessment for the acute I stage was conducted) may serve as a protective factor against hallmark symptoms of clinical depression. These symptoms, however, may emerge at the follow-up stage, when individuals with SCI are back at home (note that the BDI-II assessment for the late stage was conducted in the outpatient department). This interpretation is consistent with the recent findings of Gopalan et al.^40^, who found that individuals with SCI assessed after discharge in a home setting had higher non-somatic depressive symptom scores, including greater suicidal ideation, than those assessed during inpatient rehabilitation within the first year after injury. While these findings do not establish the mechanisms underlying this association, they are in line with the notion that the early phase of community reintegration may represent a period of increased psychological vulnerability, underscoring the importance of systematic mental health screening during this transition.

Overall, when individuals with SCI exhibited clinically relevant degrees of overall depressive symptoms, these were primarily associated with physical-somatic issues. We hypothesize that these issues are more likely to reflect direct consequences of the injury rather than clinical depression per se, even though the possibility that they represent somatic manifestations of a depressive state cannot be entirely excluded. For instance, changes in sexuality and reduced libido in individuals with SCI are influenced by the neurogenic sexual dysfunction. To ensure that a clinically relevant degree of overall depressive symptoms is not overlooked in individuals with SCI over the long term, it is important to closely monitor the development of symptoms more characteristic of clinical depression. These may not primarily involve physical-somatic aspects, but may instead reflect self-blame, self-hatred, anhedonia, and cognitive stress. Such symptoms may not be particularly pronounced early after injury, but could emerge over the course of treatment and could become more apparent at the late stage.

All in all, our findings based on the BDI-II suggest a distinction between physical-somatic and non-somatic (cognitive/affective) depressive symptoms in individuals with SCI. This is consistent with existing factor-analytic findings for the BDI-II in non-SCI populations^41^, which have frequently supported either a two-factor structure consisting of cognitive and somatic-affective dimensions^42,43^ or a three-factor structure distinguishing somatic, cognitive, and affective symptoms^44^. Specifically in SCI cohorts, a two-factor structure distinguishing somatic and non-somatic depressive symptoms based on the Patient Health Questionnaire-9 (PHQ-9^21,45^) has also been proposed^46,47^, which is consistent with our initial observations based on the BDI-II.

In the context of SCI individuals, it may overall be preferable to use the BDI-FS (FastScreen)^48^ for medically predisposed people as a screening tool for depressive symptoms instead of the BDI-I or BDI-II. BDI-FS is an effective inventory to quantify depression that reflects the cognitive and affective symptoms of depression, but excludes symptoms that are potentially related to medical problems and thus may confound diagnosis. While the utility of the BDI-FS has been demonstrated, e.g., for medical patients in the general German population^49^, it has not yet been tested or validated in the SCI population examined in the present study.

Regarding our RF model for predicting the occurrence of a clinically relevant degree of overall depressive symptoms at late stage in individuals with SCI based on features from the acute I stage, the most important features included both psychiatric (BDI-II items worthlessness, tiredness, and indecisiveness; the overall variable BinTotalBDI) and SCI-specific neurological (UEMS, TPP, TLT) and functional (SCIM-III self-care) parameters. The RF model showed a mediocre predictive performance (e.g., median AUROC of 0.60), which is consistent with recent observations^50,51^ indicating that machine learning or (even) deep learning approaches have some potential in distinguishing depressive individuals from HCs, but are nevertheless limited, possibly due to the well-known heterogeneity of depression pathology. Given its limited discriminative utility in clinical settings and the absence of validation in an independent cohort, the translational value of our model remains limited at present. We found that the demographic variables age and in particular gender were poor predictors (Supplementary Material, Section S5.2), which aligns with the mixed results from previous studies examining their role as risk factors: some reported them as relevant^17,52^, whereas others did not^53,54^. The performance of depression-related prediction models may benefit from including psychosocial variables as features, as these have been reported as potential risk factors for developing depression after SCI^54^. However, such variables were not available in our dataset.

Multiple findings from our study point to a limited (if any) relationship between psychiatric and SCI-specific variables, as well as between the development of a clinically relevant degree of overall depressive symptoms and neurological or functional recovery after SCI in our cohort. First, there were notably only few significant (negative) correlations between psychiatric BDI-II items and neurological or functional SCI parameters, and exploratory factor analyses showed that SCI-specific neurological and functional parameters, on the one hand, and psychiatric BDI-II items, on the other, loaded onto clearly distinct factors. Second, subgroup analyses within the SCI cohort did not reveal a significant relationship between the development or resolution of a clinically relevant degree of overall depressive symptoms and neurological or functional recovery during the course of treatment. Third, the strong performance of the RF models in predicting neurological and functional recovery, respectively, relied almost exclusively on SCI-specific features from the acute I stage. In contrast, psychiatric features contributed little to model performance, suggesting that they had only a limited influence on neurological and functional recovery following SCI. As neurological recovery frequently occurs spontaneously^55^, its lack of association with a clinically relevant degree of overall depressive symptoms may clinically be expected. While previous studies have reported that depressive symptoms may negatively impact functional recovery following SCI^56,57^, we did not observe such an association in our study. Notably, this aligns with recent findings for a specific cohort of older patients undergoing geriatric rehabilitation, in whom the presence of depressive symptoms did not significantly influence functional outcomes, in particular regarding mobility^58^. However, the findings regarding the lack of a relationship between the development of a clinically relevant degree of overall depressive symptoms and neurological or functional recovery should be interpreted cautiously given the observational design, limited subgroup sample sizes, and the absence of treatment and diagnostic information. Future studies with more comprehensive psychiatric characterization are needed to clarify these relationships.

## Limitations

While our cohort from the EMSCI was reasonably representative of traumatic and ischemic SCI participants, key limitations of our study arise from the limited availability of psychiatry-related data apart from the BDI. First, there were no formal MDD diagnostic labels within the SCI cohort. Consequently, we relied on BDI items to operationalize depressive symptoms in this group. However, this approach does not equate to a formal MDD diagnosis based on established criteria, such as those in the DSM-5. Hence, our analysis assumes that BDI items adequately capture depressive symptoms, which remains an approximation. Exclusively focusing on the BDI, our study also did not consider other depression scales commonly used in SCI research, such as the PHQ-9^21,45^ or the Hospital Anxiety and Depression Scale (HADS^59^). Second, although we assume that individuals with SCI received psychological support as part of standard care during hospitalization in SCI units adhering to EMSCI, no data were available on pharmacological treatments which may have been initiated between the acute I and the late stage. Third, there was no information on pre-injury psychiatric diagnoses, including MDD.

Moreover, cross-cohort comparisons were here based on the 17-item BDI-I/II Synchronized. This instrument was introduced as an ad-hoc measure derived from the intersection of the BDI-I and BDI-II versions and served as a framework for harmonizing symptom items that were directly comparable across BDI versions. Such an approach does not establish measurement equivalence across BDI versions, cohorts, clinical contexts, languages, or settings. Specifically, the excluded items are not clinically equivalent and may contain clinically meaningful information. Hence, their omission may affect interpretation. Accordingly, the BDI-I/II Synchronized should not be viewed as a psychometrically validated replacement for either full BDI version. In particular, the total BDI-I/II Synchronized score does not have a validated cut-off for classifying individuals as having a clinically relevant degree of overall depressive symptoms in our cross-cohort analyses. However, the primary focus of these analyses was on item-level symptom comparisons rather than on the interpretation of the total BDI-I/II Synchronized score itself. Furthermore, we observed high correlations between the total BDI-I/II Synchronized score (based on 17 items) and the total BDI-I and total BDI-II scores (based on 21 items each) within each considered cohort (SCI: Spearman’s rho: *ρ = 0.99*; FOR2107 MDD: *ρ = 0.99*; FOR2107 HC: *ρ = 0.96*; CIMH MDD: *ρ = 0.99*; CIMH HC: *ρ = 0.95*), suggesting that the synchronized instrument captured a substantial proportion of the variance in the original instruments. Nevertheless, the cross-cohort comparisons presented in this study should be considered exploratory overall, given that they are based on a non-validated measure. More sophisticated formal harmonization techniques should thus be developed and explored in future work.

An additional limitation of the present study is the aggregation of follow-up data across two distinct time points (acute III and chronic stages; approximately six and twelve months after injury, respectively) into a single “late stage” outcome. This approach was chosen to maximize statistical power and ensure a sufficient sample size at the latest available follow-up, given attrition over time. However, this strategy introduces temporal heterogeneity, as symptom and functional outcomes may continue to evolve between the acute III and chronic stages. Indeed, exploratory analyses indicated that specifically variables related to functional recovery differed between the two follow-up windows, suggesting that corresponding trajectories may not be fully stable across this period. Consequently, the late-stage results should be interpreted as an average estimate across a variable recovery window rather than a temporally uniform endpoint. Future studies should distinguish between intermediate and long-term follow-up trajectories by explicitly incorporating time since baseline (acute I) as a covariate, thereby enabling a clearer assessment of temporal changes across follow-up periods.

Another concern is the potential confounding role of pain (including intensity, type, and interference) in the relationship between SCI and depressive symptoms. Pain is highly prevalent after SCI^60,61^ and independently associated with depressive symptoms^62^. Clinically, addressing pain may e.g. reduce depressive symptoms without the need for direct psychological intervention.

Beyond this, individuals with SCI experiencing a severe degree of overall depressive symptoms may show reduced compliance with rehabilitation and have been less likely to attend the late-stage examination, potentially leading to missing data for this subgroup.

Lastly, the comparably low sample sizes of the examined SCI subgroups limit statistical power and the generalizability of the corresponding findings.

In conclusion, we view our findings as an initial step that underscores the need for further investigation of the relationship between depressive symptoms and SCI, e.g. in future prospective studies.

## Conclusions

This work offers the first detailed insights into depressive symptoms in individuals with SCI, operationalized using BDI items. When present, these symptoms were primarily related to physical-somatic phenomena, distinguishing them from the broader range of depressive symptoms typically observed in individuals with an MDD diagnosis, which more commonly encompass self-blame, self-hatred, and other cognitive-emotional dimensions. In this study, the presence and development of depressive symptoms appeared to be largely unrelated to neurological and functional recovery from SCI.

The development of a clinically relevant degree of overall depressive symptoms from shortly after the injury (acute I stage) to the follow-up (late) stage in individuals with SCI appeared to be primarily reflected in increases in BDI scores for items related to self-hatred, anhedonia, emotional overload, or cognitive stress. To help reduce the risk of undetected development of a clinically relevant degree of overall depressive symptoms during the course of SCI treatment, it may be useful to pay particular attention to cognitive-emotional symptoms, alongside physical-somatic symptoms, which may be influenced by the direct consequences of the injury itself.

## Supplementary Information

Supplementary Information.

## Data Availability

The datasets analyzed during the current study are not publicly available, as they contain sensitive personal data that cannot be shared by the authors.
