# Supplementary material for: Characterization of depressive symptoms in individuals with spinal cord injury through the Beck Depression Inventory, and their role in recovery
Source: Sci Rep. 2026 Aug 20;16:26205. doi: 10.1038/s41598-026-66971-7 (PMC13493943; doi:10.1038/s41598-026-66971-7)
Supplement: Supplementary file 1 [file 41598_2026_66971_MOESM1_ESM.pdf]

Supplementary Material

Characterization of depressive symptoms in individuals with spinal cord injury through the Beck Depression Inventory, and their role in recovery

Roman Schefzik, Laura Heutehaus, Christian Schuld, Tobias Gradinger, Oksana Berhe, Doris Maier, Marion Saur, Klaus Röhl, Rainer Abel, Chiara Pavese, Michael Baumberger, Armin Curt, Michèle Hubli, Harvinder S. Chhabra, EMSCI Study Group, Udo Dannlowski, Tilo Kircher, Norbert Weidner and Emanuel Schwarz

Contents

S1 Supplementary methods: Spinal cord injury (SCI)-specific neurological and functional parameters 2
S2 Supplementary methods and results: SCI cohort 4
S2.1 Comparison between acute I and late stage at BDI-II item level 4
S2.2 Spearman correlations 5
S2.3 Exploratory factor analysis for BDI-II items and SCI-specific parameters 9
S3 Supplementary results: BDI-II-specific subgroups of the SCI cohort 13
S3.1 Subgroup A ("Worsened"): Individuals with SCI with a total BDI-II score < 14 at acute I stage, but a total BDI-II score ≥ 14 at late stage 14
S3.2 Subgroup B ("Recovered"): Individuals with SCI with a total BDI-II score ≥ 14 at acute I stage, but a total BDI-II score < 14 at late stage 16
S4 Supplementary data and results: Comparison of SCI cohort to FOR2107 and CIMH reference cohorts with respect to BDI-I/II Synchronized 18
S4.1 Basic characteristics and BDI-I/II Synchronized item profiles of the SCI cohort and the FOR2107 and CIMH reference cohorts 18
S4.2 Comparison of SCI cohort and FOR2107 cohort 23
S4.3 Comparison of SCI cohort and CIMH cohort 24
S5 Supplementary methods and results: Prediction models 25
S5.1 Details on the implementation of the prediction models 25
S5.2 Predicting the occurrence of a clinically relevant degree of overall depressive symptoms at late stage 28
S5.3 Predicting neurological recovery 31
S5.4 Predicting functional recovery 38
S6 Supplementary results: SCI cohort definition and selection 45
S7 Supplementary methods and results: Cross-cohort comparisons based on PCA using polychoric correlations 49

S1 Supplementary methods: Spinal cord injury (SCI)-specific neurological and functional parameters

For the SCI cohort, we considered the following common SCI-specific assessments (Rupp et al., 2021). Standardized neurological examination was performed according to the International Standards for Neurological Classification of Spinal Cord Injury (ISNCSCI) (Kirshblum et al., 2011), which includes the upper extremity motor score (UEMS), lower extremity motor score (LEMS), total light touch (TLT) and total pin prick (TPP) scores (Vasquez et al., 2013) and the resulting neurological level of injury (here split into the three categories cervical, thoracic and lumbar) and injury severity (AIS A through E; American Spinal Injury Association (ASIA) impairment scale (AIS); Roberts et al. (2017)). Functional assessment was performed using the spinal cord independence measure version III (SCIM-III; Itzkovich et al. (2007)), covering three subscales referring to self-care, respiration and sphincter management (RSM), and mobility, respectively. An overview of the SCI-specific assessments can be found in Supplementary Table S1.

In the context of our work, neurological recovery was defined as an improvement from the acute I to the late stage in terms of AIS, UEMS, and TLT, respectively. Analogously, functional recovery was defined as an improvement with respect to the SCIM-III subscales referring to self-care, RSM, and mobility, respectively. An AIS improvement was defined as an improvement of at least one AIS grade. Improvements in UEMS, TLT, or the SCIM-III subscales were defined as a corresponding normalized percentage increase of at least 25%[1]. Here, for a fixed SCI individual, a normalized percentage change NPC_X of a score of a variable $X \in \{\mathrm{UEMS}, \mathrm{TLT}, \text{SCIM-III Self-Care}, \text{SCIM-III RSM}, \text{SCIM-III Mobility}\}$ from the acute I to the late stage expressed the proportion of potential increase (i.e., improvement) or decrease (i.e., worsening) achieved and was derived as follows:

$$\mathrm{NPC}_X := \begin{cases} \frac{\Delta_X}{\mathrm{Max}_X - x_{\mathrm{acute\,I}}} \times 100\%, & \text{if } \Delta_X > 0, \\ \frac{\Delta_X}{x_{\mathrm{acute\,I}}} \times 100\%, & \text{if } \Delta_X < 0, \\ 0\%, & \text{if } \Delta_X = 0, \end{cases}$$

where $\Delta_X := x_{\mathrm{late}} - x_{\mathrm{acute\,I}}$ denotes the difference of the scores x_{late} and $x_{\mathrm{acute\,I}}$ for X at the late and acute I stages, respectively, and Max_X the maximum possible value of X (e.g., for $X = \mathrm{UEMS}$, $\mathrm{Max}_X = 50$; see Supplementary Table S1). Note that $\mathrm{NPC}_X \in [-100\%, 100\%]$, with -100% and 100% representing the maximum potential decrease (i.e., worsening) and increase (i.e., improvement), respectively.

[1] A 25% normalized percentage increase was used here as a harmonized responder threshold to define relevant neurological and functional recovery. This threshold was selected to ensure comparability across outcome measures with differing ranges. It exceeds published minimal clinically important differences for UEMS (Scivoletto et al., 2013) and SCIM-III subscores (Corallo et al., 2017) and corresponds to substantial (and not only subtle) changes relative to the full scale range. However, as no validated minimal clinically important differences exist for normalized percentage changes, our definition should be viewed as a pragmatic, effect-size-based criterion rather than a formally established clinical threshold.

Table S1: Overview of spinal cord injury (SCI)-specific (A) neurological and (B) functional parameters (Itzkovich et al., 2007; Kirshblum et al., 2011; Roberts et al., 2017; Vasquez et al., 2013). For details regarding AIS classification, see Table 1 in Roberts et al. (2017). NLI: neurological level of injury

A) Neurological parameters

Name	Description	Range of values	Orientation
UEMS	*Upper extremity motor score*: evaluates motor function for 5 upper extremity muscle groups	0-50	The higher the value the more normal the function
LEMS	*Lower extremity motor score*: evaluates motor function for 5 lower extremity muscle groups	0-50	The higher the value the more normal the function
TLT	*Total light touch score*: assesses cutaneous sensibility through light touch	0-112	The higher the value the more normal the function
TPP	*Total pin prick score*: assesses the ability to feel a pinprick and to discriminate between sharp and dull	0-112	The higher the value the more normal the function
AIS	*American Spinal Cord Injury Association (ASIA) impairment scale*: classifies the SCI degree based on a standardized examination consisting of a myotomal-based motor examination, dermatomal-based sensory examination and an anorectal examination	A complete SCI: no sensory or motor function in the sacral segments S4–S5<br>B incomplete SCI: sensory incomplete, motor complete<br>C incomplete SCI: sensory and motor incomplete<br>D incomplete SCI: sensory and motor incomplete (at least half of the key muscles below the NLI have a grade of at least 3)<br>E normal sensory and motor function<br>[for details regarding AIS B-D, see Table 3 in Roberts et al. (2017)]	A-E: from maximum SCI severity to no severity
Injury Level	Level where the SCI occurred	Cervical, thoracic, lumbar	----

B) Functional parameters

Name	Description	Range of values	Orientation
SCIM-III Self-Care	*Spinal cord independence measure, version III, subscale referring to self-care*: disability scale specifically developed for SCI individuals to evaluate daily activities related to self-care (feeding, bathing, dressing, grooming)	0-20	The higher the value the more normal the function
SCIM-III RSM	*Spinal cord independence measure, version III, subscale referring to respiration and sphincter management*: disability scale specifically developed for SCI individuals to evaluate daily activities related to respiration and sphincter management (breathing, bladder management, bowel management, toilet use)	0-40	The higher the value the more normal the function
SCIM-III Mobility	*Spinal cord independence measure, version III, subscale referring to mobility*: disability scale specifically developed for SCI individuals to evaluate daily activities related to mobility (transfers, indoor-outdoor mobility, walking/wheelchair, stair climbing)	0-40	The higher the value the more normal the function

S2 Supplementary methods and results: SCI cohort

S2.1 Comparison between acute I and late stage at BDI-II item level

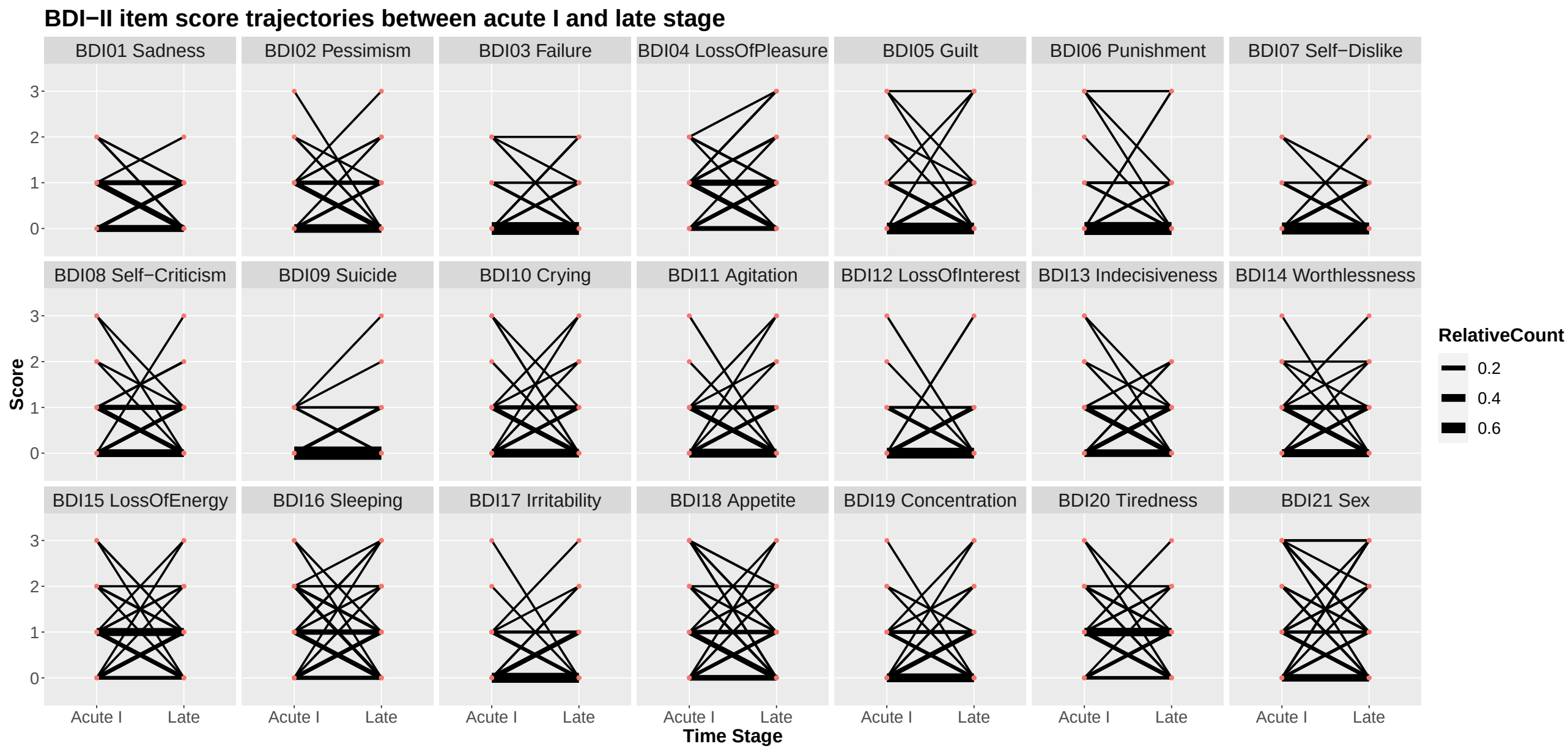

Figure S1: Trajectories from the acute I to the late stage for all BDI-II item scores for the $N = 165$-member SCI cohort. The thicker the line the more frequently the respective trajectory occurred among the considered individuals with SCI.

Comparing individual BDI-II item scores at acute I and late stage (see the trajectories of single items in Figure S1), these were significantly greater for acute I stage than late stage for appetite ($p = 0.00014$), sadness ($p = 0.00017$), sleeping ($p = 0.011$), pessimism ($p = 0.022$) and tiredness ($p = 0.030$), considering paired one-sided Wilcoxon tests.
In contrast, individual BDI-II item scores were significantly smaller for acute I stage than late stage for irritability ($p = 0.015$), suicide ($p = 0.017$) and concentration ($p = 0.018$).
Considering paired one-sided Wilcoxon tests after a Bonferroni correction for multiple testing, BDI-II item values remained significantly greater for acute I stage than late stage for appetite ($p = 0.00289$) and sadness ($p = 0.0035$) only.
In particular, all items that drove high total BDI-II scores at acute I stage were also drivers for the late stage (except for sadness and to a lesser extent appetite), namely tiredness, loss of energy, sleeping, sex and loss of pleasure, i.e., items referring to somatic issues. These driving BDI-II items were also reflected as a cluster in the heatmap representations (main text).

S2.2 Spearman correlations

S2.2.1 Acute I stage

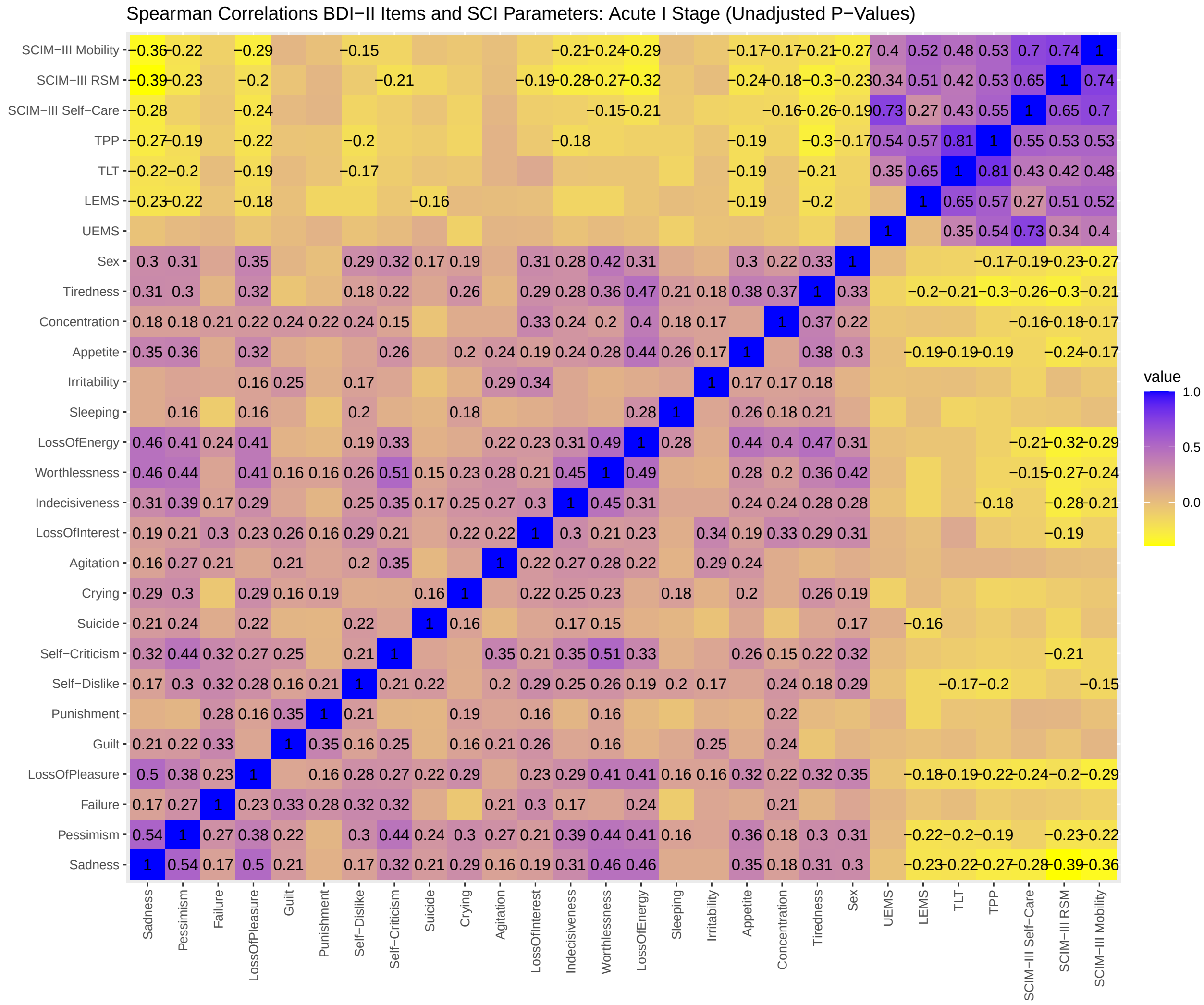

Figure S2: Map representing the Spearman correlations (based on pairwise complete observations) regarding the BDI-II items and SCI-specific parameters for the considered $N = 165$-member cohort of individuals with SCI at the acute I stage. Only correlation values that are statistically significant at the 5% level, *without adjustment for multiple testing*, are displayed.

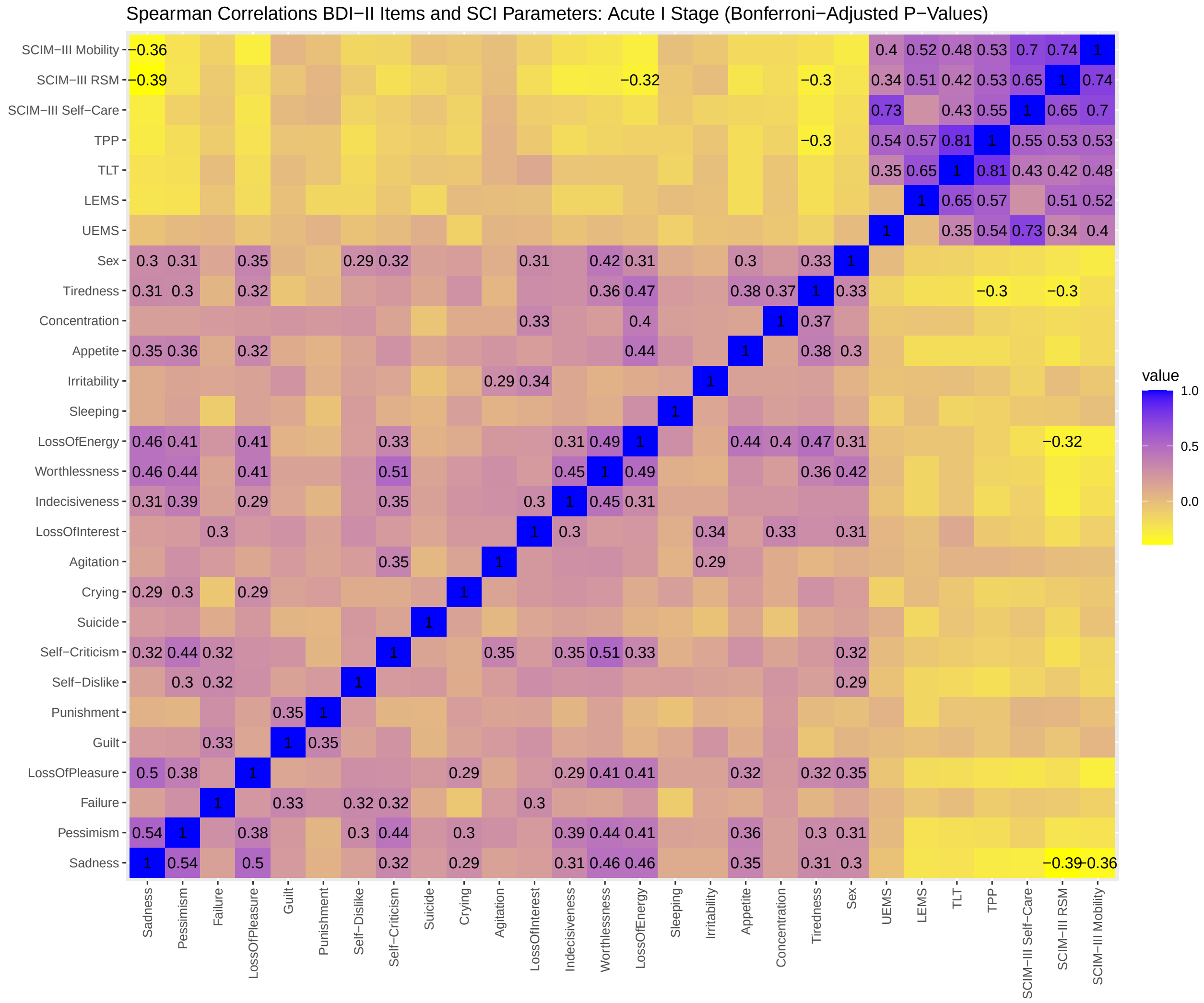

Figure S3: Map representing the Spearman correlations (based on pairwise complete observations) regarding the BDI-II items and SCI-specific parameters for the considered $N = 165$-member cohort of individuals with SCI at the acute I stage. Only correlation values that remain statistically significant at the 5% level *after Bonferroni correction for multiple testing* are displayed.

S2.2.2 Late stage

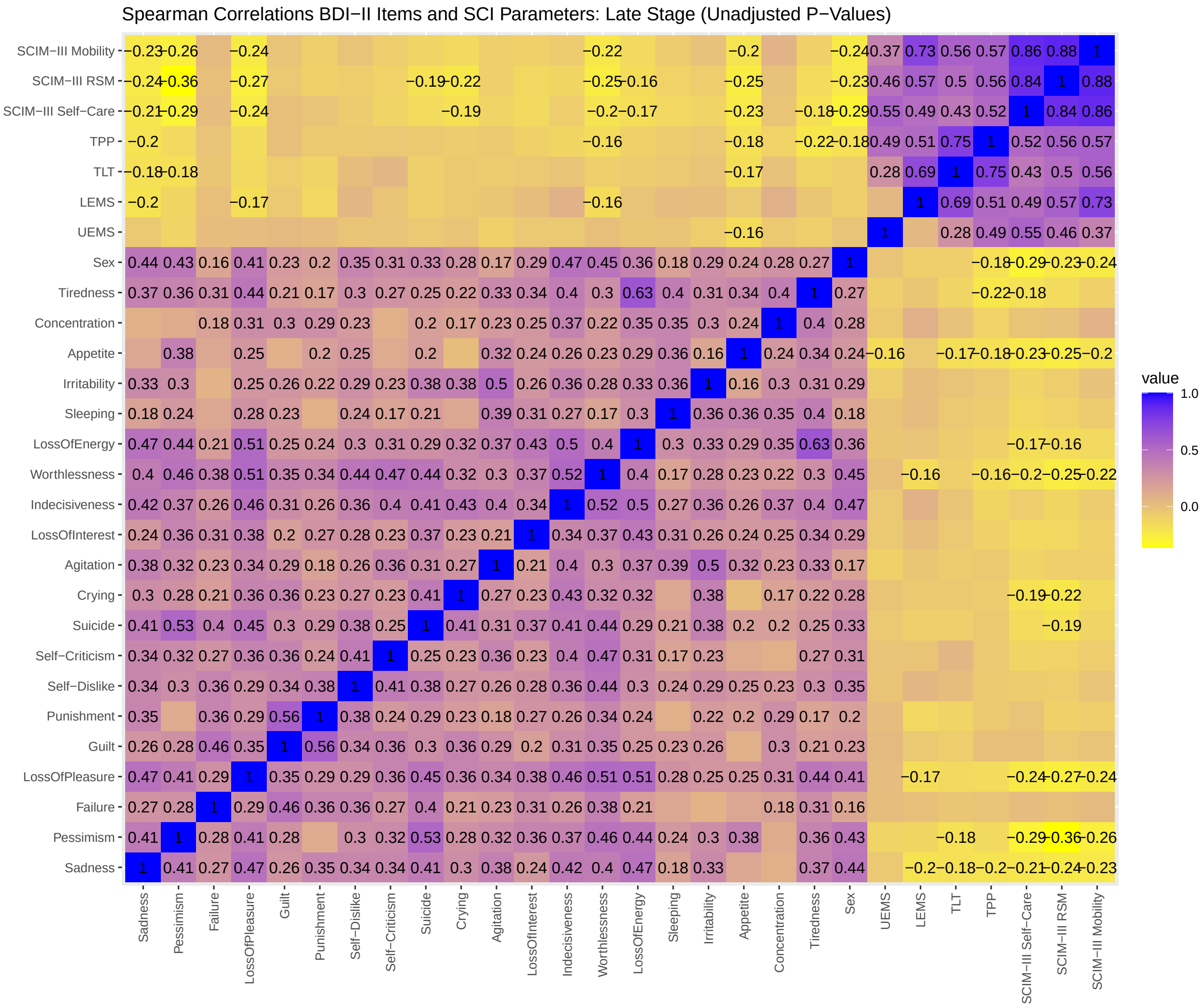

Figure S4: Map representing the Spearman correlations (based on pairwise complete observations) regarding the BDI-II items and SCI-specific parameters for the considered $N = 165$-member cohort of individuals with SCI at the late stage. Only correlation values that are statistically significant at the 5% level, *without adjustment for multiple testing*, are displayed.

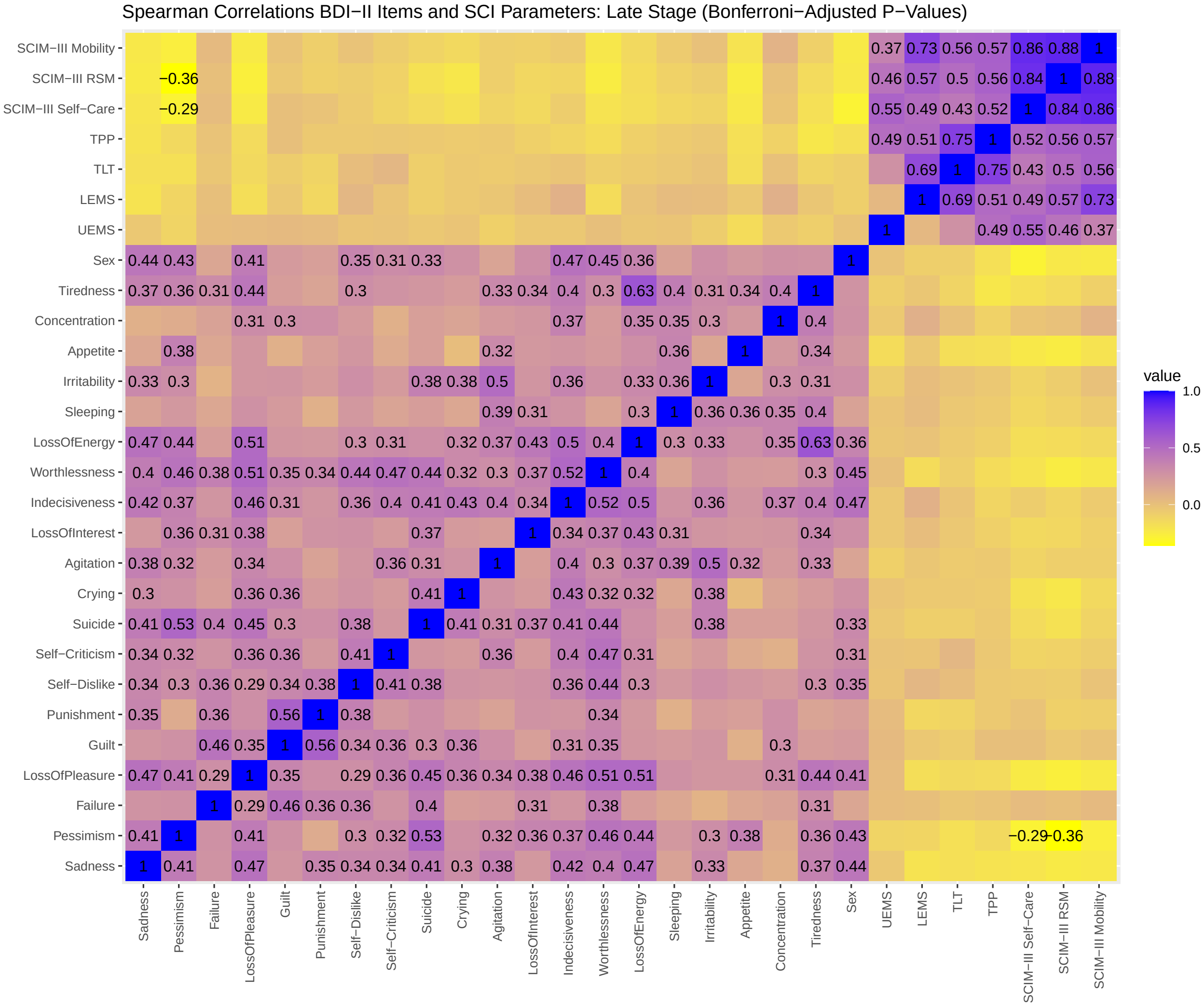

Figure S5: Map representing the Spearman correlations (based on pairwise complete observations) regarding the BDI-II items and SCI-specific parameters for the considered $N = 165$-member cohort of individuals with SCI at the late stage. Only correlation values that remain statistically significant at the 5% level *after Bonferroni correction for multiple testing* are displayed.

S2.3 Exploratory factor analysis for BDI-II items and SCI-specific parameters

We used exploratory factor analysis (EFA) at the acute I and the late stage, respectively, to explore relationships between BDI-II items and neurological and functional SCI-specific variables in SCI individuals. For each setting, we checked the suitability of the data for factor analysis via the Kaiser-Meyer-Olkin (KMO) index and determined the number of factors as the number of eigenvalues >1 of the corresponding Spearman correlation matrix (Kaiser's criterion). We then performed maximum-likelihood factor analysis using varimax rotation, including the derivation of loadings and uniqueness values.

For the acute I stage, complete data for 148 SCI individuals was used for the EFA. The KMO index was 0.79, indicating that the data was probably suitable for factor analysis. We derived an eight-factor model, with loadings and uniqueness values shown in Table S2, where we only considered loadings greater than 0.4. There was a clear separation between BDI-II items (five factors) and SCI parameters (three factors). Notably, somatic depressive symptoms formed a separate factor, self-blame emerged as a distinct dimension, and anhedonia (a key symptom for MDD diagnosis) stood on its own, reflecting its uniqueness in depression measurement. Furthermore, there was also a separation between neurological (two factors) and functional (one distinct factor) SCI-specific parameters. In a nutshell, factors could be summarized as follows:

- Factor 1: neurological SCI parameters (focus on AIS and LEMS),
- Factor 2: neurological and functional SCI parameters (focus on injury level and UEMS),
- Factor 3: self-blame (driven by failure, guilt, punishment and self-dislike),
- Factor 4: cognitive and emotional depressive symptoms, specifically involving self-hatred (driven by sadness, pessimism, worthlessness, self-criticism and loss of pleasure),
- Factor 5: somatic depressive symptoms (driven by loss of energy, tiredness, and concentration),
- Factor 6: functional SCI parameters (SCIM-III subscales),
- Factor 7: anhedonia (reflected by loss of interest),
- Factor 8: emotional reactivity (reflected by crying).

The uniqueness values reflect that some parameters (like loss of interest, AIS and LEMS) were very well explained by the extracted factors. In contrast, particularly the parameters suicide and irritability were rather poorly captured.

For the late stage, complete data for 152 SCI individuals was used for the EFA. The KMO index was 0.83, indicating that the data was probably suitable for factor analysis. We derived an eight-factor model, with loadings and uniqueness values shown in Table S3, where we only considered loadings greater than 0.4. Again, the factor model revealed a separation between BDI-II items (four factors) and SCI-specific variables (three factors). The SCI-specific parameters basically separated into neurological (two) and functional (one) factors. In a nutshell, factors could be summarized as follows:

- Factor 1: mood/depression-related (core depressive symptoms),

- Factor 2: functional SCI parameters/functional independence in SCI (driven by SCIM-III subscales),
- Factor 3: neurological SCI parameters (with a focus on AIS and LEMS),
- Factor 4: guilt or moral distress (driven by failure, guilt, and punishment),
- Factor 5: neurobehavioral symptoms (driven by sleeping, agitation, irritabikity, and concentration),
- Factor 6: neurological SCI parameters (with a focus on injury level and TPP),
- Factor 7: pessimism,
- Factor 8: empty factor, no importance (all loadings are <0.4).

The uniqueness values reflect that the SCI-specific parameters typically appeared to be better explained by the extracted factors than the BDI-II items.

Table S2: EFA results for the acute I stage: uniqueness values and loadings, where for the loadings, only values greater than 0.4 are displayed

Uniquenesses:

Sadness	Pessimism	Failure	LossOfPleasure
0.499	0.466	0.416	0.580
Guilt	Punishment	Self-Dislike	Self-Criticism
0.460	0.236	0.565	0.570
Suicide	Crying	Agitation	LossOfInterest
0.880	0.492	0.683	0.038
Indecisiveness	Worthlessness	LossOfEnergy	Sleeping
0.710	0.447	0.362	0.776
Irritability	Appetite	Concentration	Tiredness
0.853	0.597	0.519	0.487
Sex	InjuryLevel	AIS	UEMS
0.671	0.298	0.114	0.309
LEMS	TLT	TPP	SCIM-III Self-Care
0.118	0.146	0.165	0.124
SCIM-III RSM	SCIM-III Mobility		
0.181	0.173		

Loadings:

	Factor1	Factor2	Factor3	Factor4	Factor5	Factor6	Factor7	Factor8
Sadness				0.599				
Pessimism				0.652				
Failure			0.695					
LossOfPleasure				0.437				
Guilt			0.681					
Punishment			0.841					
Self-Dislike			0.525					
Self-Criticism				0.492				
Suicide								
Crying								0.616
Agitation								
LossOfInterest							0.815	
Indecisiveness								
Worthlessness				0.619				
LossOfEnergy					0.675			
Sleeping								
Irritability								
Appetite					0.502			
Concentration					0.573			
Tiredness					0.639			
Sex					0.412			
InjuryLevel		0.780						
AIS	0.921							
UEMS		0.797						
LEMS	0.867							
TLT	0.669	0.623						
TPP	0.545	0.685						
SCIM-III Self-Care		0.613				0.671		
SCIM-III RSM						0.706		
SCIM-III Mobility						0.739		

Table S3: EFA results for the late stage: uniqueness values and loadings, where for the loadings, only values greater than 0.4 are displayed. Note that Factor 8 is omitted here, as no loading was greater than 0.4.

Uniquenesses:

Sadness	Pessimism	Failure	LossOfPleasure
0.567	0.347	0.591	0.492
Guilt	Punishment	Self-Dislike	Self-Criticism
0.327	0.291	0.520	0.505
Suicide	Crying	Agitation	LossOfInterest
0.571	0.718	0.480	0.484
Indecisiveness	Worthlessness	LossOfEnergy	Sleeping
0.494	0.376	0.463	0.481
Irritability	Appetite	Concentration	Tiredness
0.472	0.702	0.664	0.490
Sex	InjuryLevel	AIS	UEMS
0.624	0.466	0.102	0.218
LEMS	TLT	TPP	SCIM-III Self-Care
0.118	0.131	0.181	0.087
SCIM-III RSM	SCIM-III Mobility		
0.177	0.005		

Loadings:

	Factor1	Factor2	Factor3	Factor4	Factor5	Factor6	Factor7
Sadness	0.597						
Pessimism	0.556						0.479
Failure				0.479			
LossOfPleasure	0.584						
Guilt				0.759			
Punishment				0.761			
Self-Dislike	0.573						
Self-Criticism	0.604						
Suicide	0.434						
Crying	0.411						
Agitation					0.573		
LossOfInterest	0.568						
Indecisiveness	0.643						
Worthlessness	0.690						
LossOfEnergy	0.658						
Sleeping					0.703		
Irritability	0.468				0.478		
Appetite							
Concentration					0.449		
Tiredness	0.534						
Sex	0.584						
InjuryLevel						0.656	
AIS			0.922				
UEMS		0.690				0.489	
LEMS			0.865				
TLT			0.734			0.488	
TPP			0.484			0.639	
SCIM-III Self-Care		0.906					
SCIM-III RSM		0.837					
SCIM-III Mobility		0.818	0.504				

S3 Supplementary results: BDI-II-specific subgroups of the SCI cohort

Among the $N = 165$ individuals with SCI with complete BDI-II item profiles for both acute I and late stage, 35 had a total BDI-II score ≥ 14 (indicating a clinically relevant degree of overall depressive symptoms) at the late stage, where 16 thereof already had a total BDI-II score ≥ 14 at the acute I stage, while 19 had not. In contrast, 130 individuals had a total BDI-II score < 14 (indicating a clinically irrelevant degree of overall depressive symptoms) at the late stage, where 105 thereof already had a total BDI-II score < 14 at the acute I stage, while 25 had not.
Against this background, we specifically focused on the following clinically important, BDI-II-specific subgroups of SCI individuals here:

a. *Subgroup A ("Worsened")*: 19 individuals with SCI with a total BDI-II score < 14 at acute I stage, but a total BDI-II score ≥ 14 at late stage (i.e., individuals with SCI that developed a clinically relevant degree of overall depressive symptoms over time), and

b. *Subgroup B ("Recovered")*: 25 individuals with SCI with a total BDI-II score ≥ 14 at acute I stage, but a total BDI-II score < 14 at late stage (i.e., individuals with SCI that recovered from a clinically relevant degree of overall depressive symptoms over time).

S3.1 Subgroup A ("Worsened"): Individuals with SCI with a total BDI-II score < 14 at acute I stage, but a total BDI-II score ≥ 14 at late stage

For the 19 individuals with SCI in Subgroup A, total BDI-II scores significantly increased from a clinically irrelevant level at the acute I (median: 8) stage to a clinically relevant level at the late (median: 19) stage (paired Wilcoxon rank sum test; $p = 7.1 \times 10^{-5}$).

At an individual BDI-II item level, scores were significantly greater (paired Wilcoxon rank sum tests with Bonferroni adjustment) at the late stage compared to the acute I stage for loss of interest ($p = 0.0057$), indecisiveness ($p = 0.0067$), loss of pleasure ($p = 0.0075$), agitation ($p = 0.0076$), suicide ($p = 0.0097$), irritability ($p = 0.012$), loss of energy ($p = 0.023$), self-dislike ($p = 0.026$), guilt ($p = 0.028$) and sleeping ($p = 0.036$). For driving BDI-II items, already high scores at the acute I stage stayed high (tiredness, sex) or even significantly increased (loss of pleasure, loss of energy, sleeping) at the late stage. Additionally, also the scores of other items (that are no driving items at the acute I stage) significantly increased: this held for loss of interest (relating to anhedonia as a key symptom for MDD diagnosis; notably, there were only scores of 0 at the acute I stage), indecisiveness, agitation, suicide, irritability, self-dislike and guilt, i.e., items relating to non-somatic phenomena (Figure S6). In summary, to ensure that the development of a clinically relevant degree of overall depressive symptoms does not go undetected, it is recommended to specifically monitor items related to anhedonia, emotional stress, or self-blame (rather than physical-somatic issues). These symptoms may not be present during the acute I stage, but can become more pronounced at the late stage.

Overall, the development of a clinically relevant degree of overall depressive symptoms from the acute I to the late stage was not associated with neurological or functional recovery. In the total $N = 165$-member SCI cohort, Fisher's exact tests confirmed that there was indeed no statistically significant association between being in Subgroup A and neurological recovery with respect to AIS ($p = 0.394$), UEMS ($p = 0.170$), or TLT ($p = 0.235$). Likewise, no statistically significant association was observed between being in Subgroup A and functional recovery with respect to the SCIM-III subscales referring to self-care ($p = 0.789$), RSM ($p = 0.586$), or mobility ($p = 0.445$).

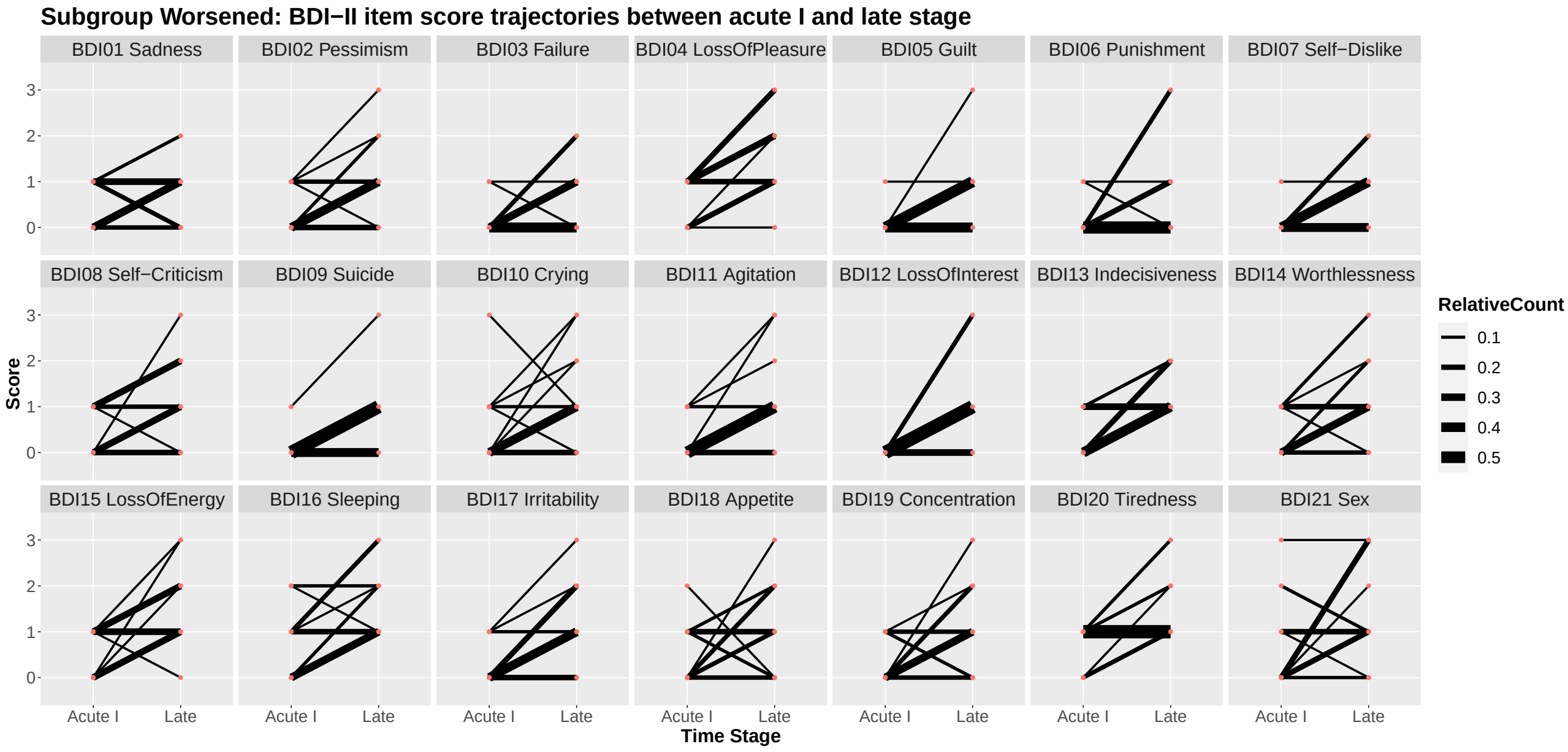

Figure S6: Trajectories from the acute I to the late stage for all BDI-II item scores for the 19-member Subgroup A of individuals with SCI that developed a clinically relevant degree of overall depressive symptoms over time (total BDI-II score ≥ 14 at late stage, but not at acute I stage). The thicker the line the more frequently the respective trajectory occurred among the considered individuals with SCI.

S3.2 Subgroup B ("Recovered"): Individuals with SCI with a total BDI-II score ≥ 14 at acute I stage, but a total BDI-II score < 14 at late stage

For the 25 individuals with SCI in Subgroup B, total BDI-II scores significantly decreased from the a clinically relevant level at the acute I (median: 18) stage to a clinically irrelevant level at the late (median: 8) stage (paired Wilcoxon rank sum test; $p = 6.4 \times 10^{-6}$).
At an individual BDI-II item level, scores were significantly smaller (paired Wilcoxon rank sum tests with Bonferroni adjustment) at the late stage compared to the acute I stage for sex ($p = 0.0009$), appetite ($p = 0.0012$), loss of pleasure ($p = 0.0056$), sadness ($p = 0.0096$), loss of energy ($p = 0.020$), sleeping ($p = 0.024$), tiredness ($p = 0.032$) and pessimism ($p = 0.037$). That said, in particular the scores of the driving BDI-II items sex, appetite, tiredness, sleeping, loss of energy and loss of pleasure significantly decreased from the acute I to the late stage (where the scores of the other items stayed at low levels). Hence, for the SCI individuals in Subgroup B, predominantly physical-somatic items were pronounced at the acute I stage, but improved by the late stage, leading to an improvement of the degree of overall depressive symptoms to a clinically irrelevant level (Figure S7). Although these individuals were above the cut-off for a clinically relevant degree of overall depressive symptoms at the acute I stage, this was solely due to somatic issues, likely as a direct consequence of the injury. Therefore, in such cases, where elevated scores are limited to physical-somatic items shortly after injury, unnecessary treatment with antidepressants may ideally be avoided.
Overall, recovery from a clinically relevant degree of overall depressive symptoms from the acute I to the late stage was not associated with functional or neurological recovery. In the total $N = 165$-member SCI cohort, Fisher's exact tests confirmed that there was indeed no statistically significant association between being in Subgroup B and functional recovery with respect to the SCIM-III subscales referring to self-care ($p = 1$), RSM ($p = 0.818$), or mobility ($p = 0.180$). Likewise, no statistically significant association was observed between being in Subgroup B and neurological recovery with respect to UEMS ($p = 1$) or TLT ($p = 0.438$). A weakly significant association was found only between being in Subgroup B and neurological recovery with respect to AIS ($p = 0.038$).

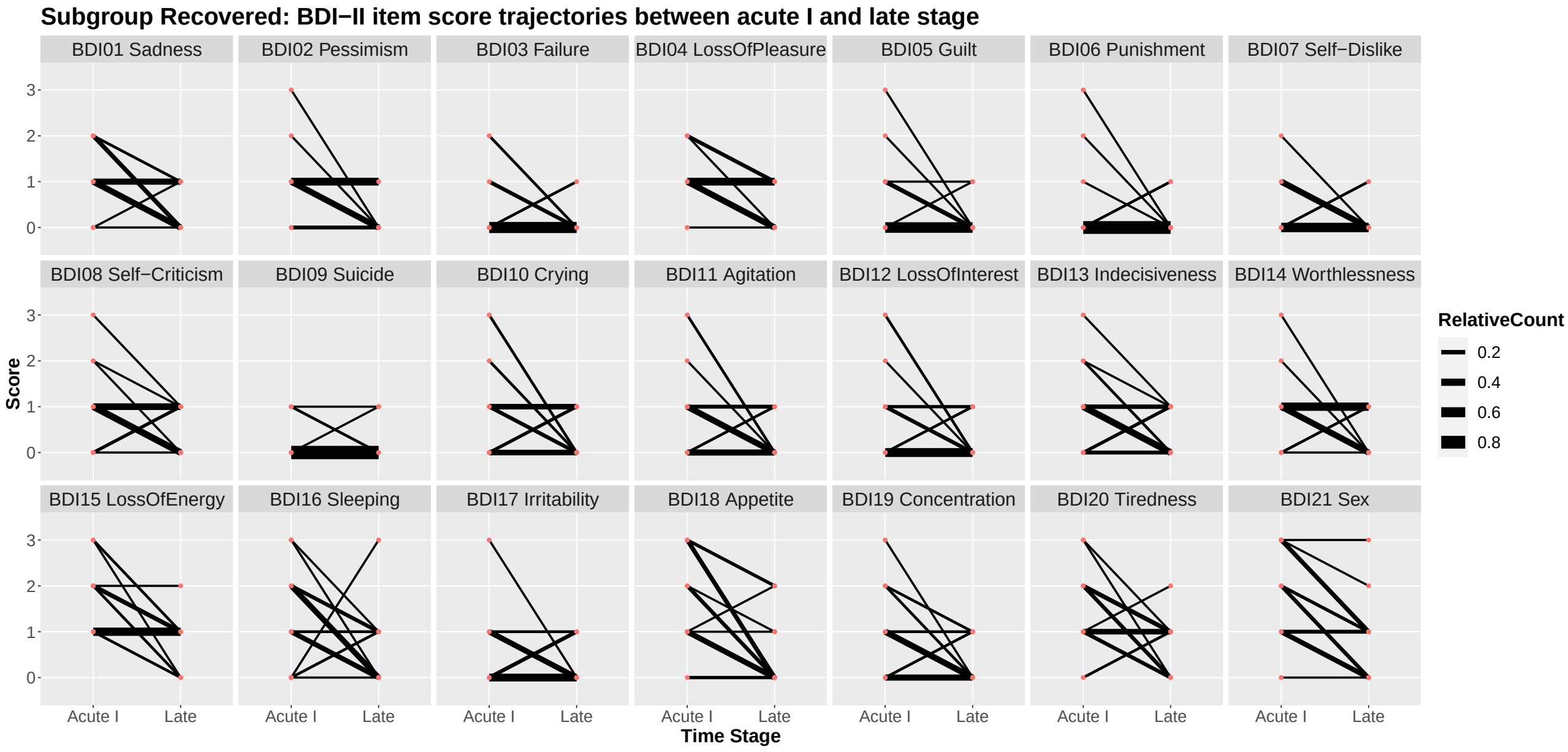

Figure S7: Trajectories from the acute I to the late stage for all BDI-II item scores for the 25-member Subgroup B of individuals with SCI that recovered from a clinically relevant degree of overall depressive symptoms over time (total BDI-II score < 14 at late stage, but not at acute I stage). The thicker the line the more frequently the respective trajectory occurred among the considered individuals with SCI.

S4 Supplementary data and results: Comparison of SCI cohort to FOR2107 and CIMH reference cohorts with respect to BDI-I/II Synchronized

S4.1 Basic characteristics and BDI-I/II Synchronized item profiles of the SCI cohort and the FOR2107 and CIMH reference cohorts

We compared the BDI-I/II Synchronized item profiles from individuals with SCI at the *late stage* to those of HCs and individuals with an MDD diagnosis, respectively, from the FOR2107 and the CIMH reference cohorts, respectively.

The data from the FOR2107 cohort had been collected in German psychiatric hospitals in Marburg and Münster, respectively, as part of the longitudinal FOR2107 study (Kircher et al., 2019), gathering data from a baseline time point and a follow-up time point after two years. To allow for a consistent comparison to the *late stage* focused on in the SCI cohort, we here only considered the *follow-up time point* in the FOR2107 study. MDD in the FOR2107 cohort was diagnosed using DSM-5.

The data from the CIMH cohort had been collected at the Central Institute of Mental Health in Mannheim, Germany, as part of the IntegraMent study (Schwarz et al., 2020). MDD in the CIMH cohort was diagnosed based on DSM-IV.

Overall, age structure and gender ratio in the FOR2107 and CIMH cohorts were quite similar among individuals with MDD diagnosis. Compared to the SCI cohort, individuals with MDD diagnosis in the reference cohorts were generally younger and had a higher proportion of females (Table S4).

The SCI cohort consisted of $N = 165$ individuals. For this cohort, the highest BDI-I/II Synchronized scores were obtained for the items tiredness (mean: 0.79), loss of pleasure (mean: 0.76), sleeping (mean: 0.73), sex (mean: 0.68), and appetite (mean: 0.55), i.e., items relating to physical-somatic phemomena (Figure S8).

The FOR2107 data comprised 614 HCs and 134 individuals with MDD diagnosis. For the individuals diagnosed with MDD (specific focus here), the highest BDI-I/II Synchronized scores were obtained for the items tiredness (mean: 1.31), failure (mean: 1.2), loss of pleasure (mean: 1.16), self-dislike (mean: 1.14), pessimism (mean: 1.13), and sleeping (mean: 1.13), covering a broad range of depressive symptoms (Figure S9).

The CIMH data comprised 181 HCs and 43 individuals diagnosed with MDD. For the individuals with MDD diagnosis (specific focus here), the highest BDI-I/II Synchronized scores were obtained for the items self-criticism (mean: 1.47), indecisiveness (mean: 1.37), tiredness (mean: 1.3), self-dislike (mean: 1.26), sex (mean: 1.26), and pessimism (mean: 1.23), covering a broad range of depressive symptoms (Figure S10).

Table S4: Summary statistics for gender, age and total BDI-I/II Synchronized score of the HCs and the individuals with MDD diagnosis (MDD), respectively, in the FOR2107 and CIMH reference cohorts, respectively, in comparison to the SCI cohort (late stage, with age referring to the date of injury). For comparison, the original summary statistics of the total BDI-I score (for the FOR2107 and CIMH cohorts) and total BDI-II score (for the SCI cohort) are also provided.

		FOR2107		CIMH		SCI
		HCs ($N = 614$)	MDD ($N = 134$)	HCs ($N = 181$)	MDD ($N = 43$)	($N = 165$)
Gender	male	216 (35.2%)	39 (29.1%)	97 (53.6%)	11 (25.6%)	125 (75.8%)
	female	398 (64.8%)	95 (70.9%)	85 (46.4%)	32 (74.4%)	40 (24.2%)
Age, yr	mean (SD)	37.5 (13.3)	39.6 (14.0)	31.2 (11.4)	36.8 (11.7)	47.0 (19.1)
	median (min-max)	32 (20-68)	34.5 (20-67)	27 (18-65)	33 (18-61)	48 (16-86)
Total BDI-I/II	mean(SD)	2.0 (2.7)	16.3 (9.4)	1.9 (2.5)	18.1 (10.6)	7.1 (6.2)
Synchronized	median (min-max)	1 (0-17)	16 (0-42)	1 (0-12)	17 (1-47)	6 (0-37)
Total BDI-I	mean(SD)	2.6 (3.3)	19.6 (11.0)	2.5 (3.2)	22.1 (12.3)	
	median (min-max)	1 (0-23)	19 (0-48)	1 (0-16)	20 (1-56)	
Total BDI-II	mean(SD)					9.1 (7.8)
	median (min-max)					8 (0-44)

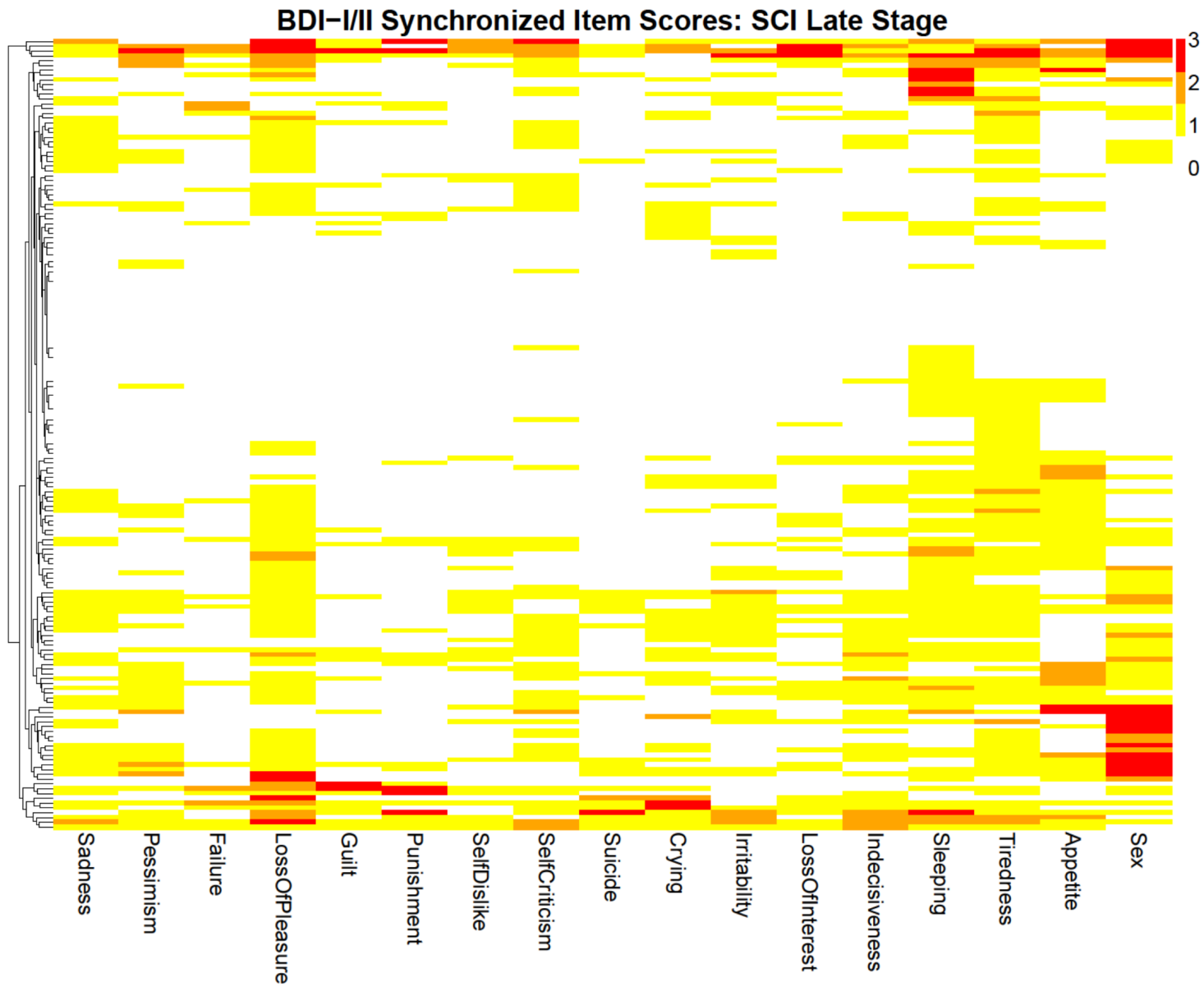

	Median	1Q	3Q	Mean	SD
Sadness	0	0	1	0.38	0.51
Pessimism	0	0	1	0.36	0.60
Failure	0	0	0	0.18	0.47
LossOfPleasure	1	0	1	0.76	0.77
Guilt	0	0	0	0.21	0.53
Punishment	0	0	0	0.22	0.60
SelfDislike	0	0	0	0.22	0.46
SelfCriticism	0	0	1	0.45	0.60
Suicide	0	0	0	0.17	0.44
Crying	0	0	1	0.36	0.59
Irritability	0	0	1	0.35	0.57
LossOfInterest	0	0	0	0.28	0.56
Indecisiveness	0	0	1	0.43	0.59
Sleeping	1	0	1	0.73	0.77
Tiredness	1	0	1	0.79	0.60
Appetite	0	0	1	0.55	0.72
Sex	0	0	1	0.68	0.97
Total BDI-I/II Synch	6	3	10	7.11	6.24

Figure S8: Overview of the 165 individuals with SCI at the late stage;
Left: Heatmap representation for the BDI-I/II Synchronized item scores (attaining values of either 0 (white), 1 (yellow), 2 (orange) or 3 (red)). Rows correspond to individuals, and columns to BDI-I/II Synchronized items. Individuals are arranged based on a hierarchical clustering as indicated by the respective dendrograms.
Right: Median together with first quantile (1Q) and third quantile (3Q), and mean together with standard deviation (SD) regarding the BDI-I/II Synchronized scores (items and total)

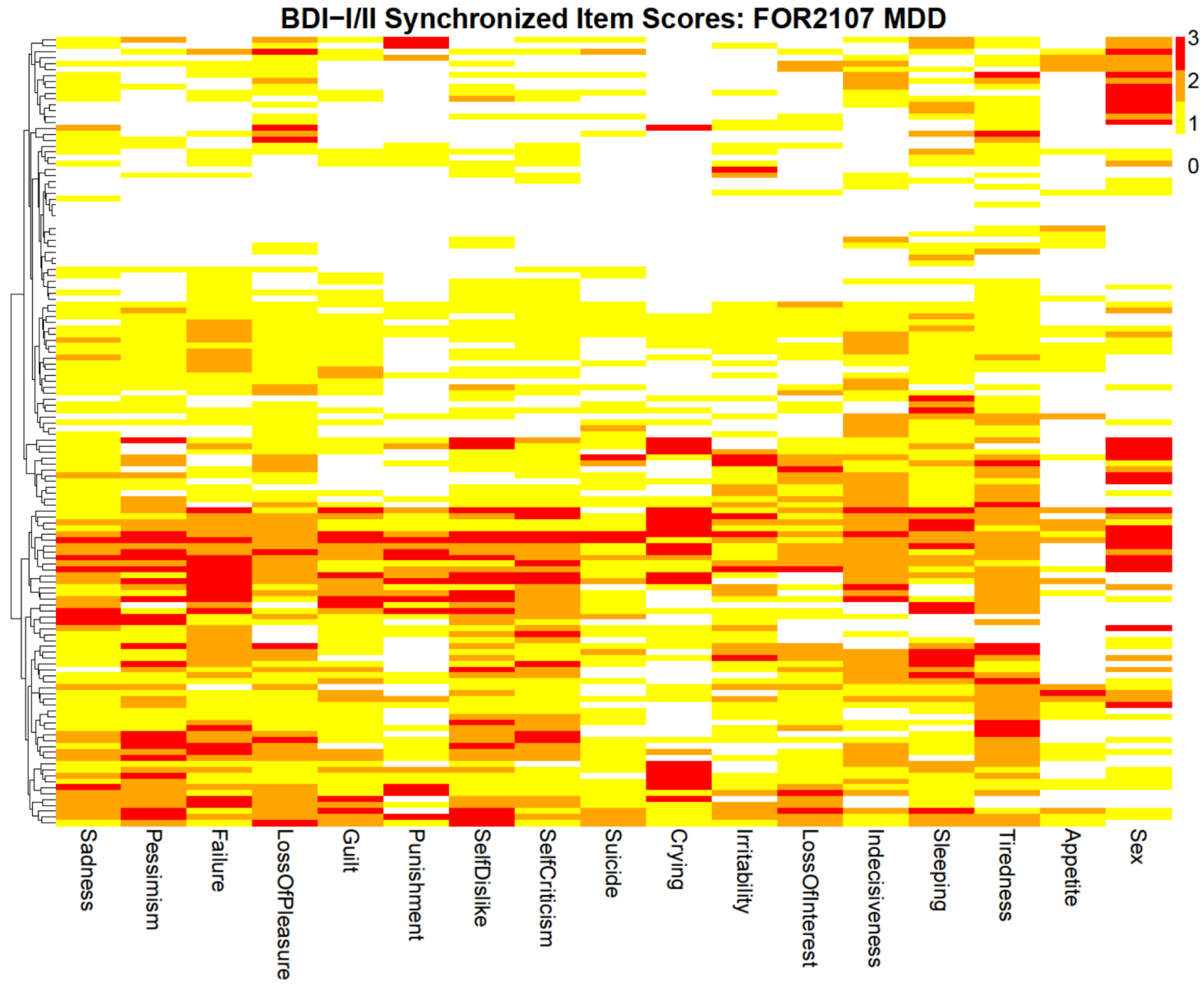

	Median	1Q	3Q	Mean	SD
**Sadness**	1	1	1.00	1.05	0.80
**Pessimism**	1	0	2.00	1.13	1.04
**Failure**	1	0	2.00	1.20	0.98
**LossOfPleasure**	1	1	2.00	1.16	0.82
**Guilt**	1	0	1.00	0.90	0.87
**Punishment**	0	0	1.00	0.66	0.93
**SelfDislike**	1	0	2.00	1.14	0.97
**SelfCriticism**	1	0	2.00	1.06	0.89
**Suicide**	1	0	1.00	0.66	0.72
**Crying**	0	0	1.00	0.70	1.05
**Irritability**	1	0	1.00	0.83	0.86
**LossOfInterest**	1	0	1.00	0.84	0.85
**Indecisiveness**	1	0	2.00	1.10	0.89
**Sleeping**	1	0	2.00	1.13	0.92
**Tiredness**	1	1	2.00	1.31	0.86
**Appetite**	0	0	1.00	0.43	0.70
**Sex**	1	0	2.00	1.03	1.15
**Total BDI-I/II Synch**	16	9	22.75	16.32	9.43

Figure S9: Overview of the 134 individuals diagnosed with MDD in the FOR2107 cohort;
Left: Heatmap representation for the BDI-I/II Synchronized item scores (attaining values of either 0 (white), 1 (yellow), 2 (orange) or 3 (red)). Rows correspond to individuals, and columns to BDI-I/II Synchronized items. Individuals are arranged based on a hierarchical clustering as indicated by the respective dendrograms.
Right: Median together with first quantile (1Q) and third quantile (3Q), and mean together with standard deviation (SD) regarding the BDI-I/II Synchronized scores (items and total)

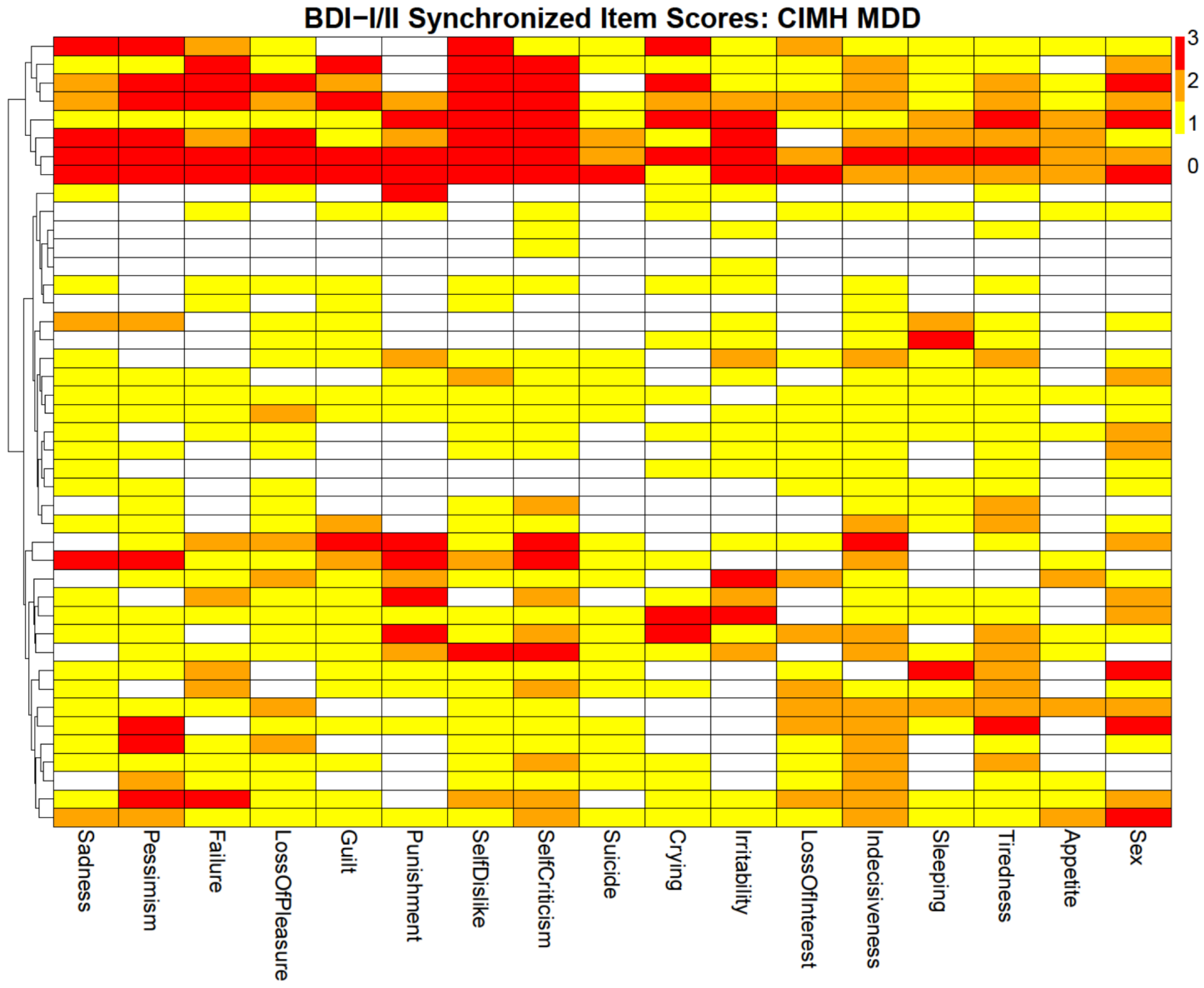

	Median	1Q	3Q	Mean	SD
Sadness	1	0.5	1.0	1.07	0.91
Pessimism	1	0.0	2.0	1.23	1.13
Failure	1	0.0	2.0	1.09	1.02
LossOfPleasure	1	1.0	1.0	1.12	0.85
Guilt	1	0.0	1.0	0.98	0.94
Punishment	1	0.0	2.0	1.00	1.18
SelfDislike	1	1.0	2.0	1.26	1.05
SelfCriticism	1	1.0	2.0	1.47	1.03
Suicide	1	0.0	1.0	0.65	0.69
Crying	1	0.0	1.0	0.88	1.00
Irritability	1	0.0	1.0	1.02	1.01
LossOfInterest	1	0.0	1.0	0.88	0.82
Indecisiveness	1	1.0	2.0	1.37	0.76
Sleeping	1	0.0	1.0	0.91	0.87
Tiredness	1	1.0	2.0	1.30	0.80
Appetite	0	0.0	1.0	0.58	0.76
Sex	1	0.0	2.0	1.26	1.03
Total BDI-I/II Synch	17	10.5	23.5	18.07	10.61

Figure S10: Overview of the 43 individuals diagnosed with MDD in the CIMH cohort;
Left: Heatmap representation for the BDI-I/II Synchronized item scores (attaining values of either 0 (white), 1 (yellow), 2 (orange) or 3 (red)). Rows correspond to individuals, and columns to BDI-I/II Synchronized items. Individuals are arranged based on a hierarchical clustering as indicated by the respective dendrograms.
Right: Median together with first quantile (1Q) and third quantile (3Q), and mean together with standard deviation (SD) regarding the BDI-I/II Synchronized scores (items and total)

S4.2 Comparison of SCI cohort and FOR2107 cohort

The total BDI-I/II Synchronized score for the $N = 165$ individuals with SCI was overall significantly greater than that of the 614 FOR2107 HCs (median of 6 for SCI vs. median of 1 for FOR2107 HCs; Wilcoxon rank sum test: $p < 2.2 \times 10^{-16}$). Also, all individual BDI-I/II Synchronized item scores were significantly greater for individuals with SCI than for FOR2107 HCs (Bonferroni-adjusted Wilcoxon rank sum tests). The FOR2107 HCs showed less spread across individuals (i.e., individuals were more similar with typically very low total BDI-I/II Synchronized scores) and clustered together far more tightly (around the origin) in the PCA biplots (Figure 5A in the main text) than the 134 FOR2107 individuals with MDD diagnosis, which revealed far more spread.

The total BDI-I/II Synchronized score for the individuals with SCI was overall significantly smaller than that of the FOR2107 individuals diagnosed with MDD (median of 6 for SCI vs. median of 16 for FOR2107 individuals with MDD diagnosis; Wilcoxon rank sum test: $p < 2.2 \times 10^{-16}$). Accordingly, the scores of all individual BDI-I/II Synchronized items were significantly smaller for the individuals with SCI than for the FOR2107 individuals with MDD diagnosis, except for the items appetite ($p = 1$), crying ($p = 0.18$) and sex ($p = 0.09$). Most obvious differences were observed for the items failure ($p = 7.8 \times 10^{-24}$), self-dislike ($p = 2.9 \times 10^{-20}$), guilt ($p = 8.4 \times 10^{-16}$), sadness ($p = 5.7 \times 10^{-14}$), suicide ($p = 7.6 \times 10^{-12}$), pessimism ($p = 3.6 \times 10^{-11}$) and indecisiveness ($p = 2.0 \times 10^{-10}$) (Bonferroni-adjusted one-sided Wilcoxon rank sum tests).

The FOR2107 individuals with MDD diagnosis showed a broad range of depressive symptoms, operationalized through the BDI-I/II Synchronized items (visual inspection in right plot in Figure 5A in the main text; Figure S9). For a (slight) majority of FOR2107 individuals with MDD diagnosis, a high total BDI-I/II Synchronized score was mainly driven by high scores for BDI-I/II Synchronized items primarily relating to self-hatred (e.g., self-dislike, self-criticism, failure and guilt; lower right quadrant in the right plot in Figure 5A in the main text) rather than by high scores for BDI-I/II Synchronized items primarily relating to physical-somatic phenomena (e.g., sex, appetite; upper right quadrant in the right plot in Figure 5A in the main text).

Regarding the individuals with SCI, a corresponding high total BDI-I/II Synchronized score for the individuals with SCI was mainly driven by high BDI-I/II Synchronized item scores for primarily sex, tiredness, sleeping, loss of pleasure and appetite (Figure S8), i.e., basically those items relating to physical-somatic effects that drove only part of the FOR2107 individuals diagnosed with MDD (upper right quadrant in the right plot in Figure 5A in the main text).

In a nutshell, individuals with SCI showed more overall depressive symptoms than FOR2107 HCs, but fewer than FOR2107 individuals with MDD diagnosis. High total BDI-I/II Synchronized scores in individuals with SCI were primarily driven by items relating to somatic-physical phenomena, whereas high total BDI-I/II Synchronized scores for FOR2107 individuals with MDD diagnosis were driven by a broader range of items, particularly those related to self-rejection.

S4.3 Comparison of SCI cohort and CIMH cohort

The total BDI-I/II Synchronized score for the $N = 165$ individuals with SCI was overall significantly greater than that of the 181 CIMH HCs (median of 6 for SCI vs. median of 1 for FOR2107 HCs; Wilcoxon rank sum test: $p < 2.2 \times 10^{-16}$). Also, all individual BDI-I/II Synchronized item scores were significantly greater for individuals with SCI than for CIMH HCs (Bonferroni-adjusted Wilcoxon rank sum tests). The CIMH HCs showed less spread across individuals (i.e., individuals were more similar with typically very low total BDI-I/II Synchronized scores) and clustered together far more tightly (around the origin) in the PCA biplots (Figure 5B in the main text) than the 43 CIMH individuals with MDD diagnosis, which revealed far more spread.

The total BDI-I/II Synchronized score for the individuals with SCI was overall significantly smaller than that of the CIMH individuals diagnosed with MDD (median of 6 for SCI vs. median of 17 for CIMH individuals with MDD diagnosis; Wilcoxon rank sum test: $p = 1.5 \times 10^{-11}$). Accordingly, the scores of all individual BDI-I/II Synchronized items were significantly smaller for the individuals with SCI than for the CIMH individuals with MDD diagnosis, except for the items appetite ($p = 1$), sleeping ($p = 1$) and loss of pleasure ($p = 0.07$). Most obvious differences were observed for the items self-dislike ($p = 3.1 \times 10^{-13}$), failure ($p = 2.8 \times 10^{-12}$), indecisiveness ($p = 1.7 \times 10^{-11}$), guilt ($p = 2.2 \times 10^{-10}$), self-criticism ($p = 1.9 \times 10^{-9}$), suicide ($p = 1.7 \times 10^{-7}$) and pessimism ($p = 8.0 \times 10^{-7}$) (Bonferroni-adjusted one-sided Wilcoxon rank sum tests).

The CIMH individuals with MDD diagnosis showed a broad range of depressive symptoms, operationalized through the BDI-I/II Synchronized items (visual inspection in right plot in Figure 5B in the main text; Figure S10). For a (slight) majority of CIMH individuals with MDD diagnosis, a high total BDI-I/II Synchronized score was mainly driven by high scores for BDI-I/II Synchronized items primarily relating to self-hatred (e.g., self-dislike, self-criticism, failure; lower right quadrant in the right plot in Figure 5A in the main text) rather than by high scores for BDI-I/II Synchronized items primarily relating to physical-somatic phenomena (e.g., appetite, sleeping; upper right quadrant in the right plot in Figure 5B in the main text).

Regarding the individuals with SCI, a corresponding high total BDI-I/II Synchronized score for the individuals with SCI was mainly driven by high BDI-I/II Synchronized item scores for primarily sex, tiredness, sleeping, loss of pleasure and appetite (Figure S8), i.e., basically those items relating to physical-somatic effects that drove only part of the CIMH individuals diagnosed with MDD (upper right quadrant in the right plot in Figure 5B in the main text).

In summary, individuals with SCI showed more overall depressive symptoms than CIMH HCs, but fewer than CIMH individuals with MDD diagnosis. High total BDI-I/II Synchronized scores in individuals with SCI were primarily driven by items relating to somatic-physical phenomena, whereas high total BDI-I/II Synchronized scores for CIMH individuals with MDD diagnosis were driven by a broader range of items, particularly those related to self-rejection.

S5 Supplementary methods and results: Prediction models

S5.1 Details on the implementation of the prediction models

We assessed whether for individuals with SCI, the outcomes of (i) the occurrence of a clinically relevant degree of overall depressive symptoms at late stage based on the total BDI-II score, (ii) neurological recovery, and (iii) functional recovery, respectively, could be predicted using various SCI-specific, psychiatric and demographic features that were measured *at the acute I stage*.
Specifically, we here considered binary classification settings, where the outcomes (outcome variables) were defined as follows:

(i) occurrence of a clinically relevant degree of overall depressive symptoms at late stage: binary variable indicating whether an SCI individual had a total BDI-II score ≥ 14 at the late stage ("1") or not ("0");

(ii) neurological recovery: binary variable indicating whether there was an improvement in terms of AIS, UEMS or TLT, respectively, from the acute I to the late stage ("1") or not ("0") (see Supplementary Material, Section S1);

(iii) functional recovery: binary variable indicating whether there was an improvement in terms of the SCIM-III Self-Care, RSM or Mobility scores, respectively, from the acute I to the late stage ("1") or not ("0") (see Supplementary Material, Section S1).

We considered the following in total 33 features:

- 9 SCI-specific features: InjuryLevel, AIS, LEMS, UEMS, TLT, TPP (neurological features), SCIM-III subscales referring to self-care, respiration and sphincter management, and mobility (functional features);

- 22 psychiatric features: the 21 BDI-II items, and a binary variable BinTotalBDI indicating whether an SCI individual had a total BDI-II score ≥ 14 at the acute I stage ("1") or not ("0") (i.e., the presence of a clinically relevant degree of overall depressive symptoms at the acute I stage);

- 2 demographic features: gender, age (at date of injury).

Only SCI individuals with complete data for all respective involved variables were used for the analyses here, see Table S5 for specifications.
As classification models, we employed random forest (RF) (Breiman, 2001) approaches, using the implementation from the R package `randomForest` (Liaw and Wiener, 2002), where the involved hyperparameters were tuned as follows:

- The maximum number of terminal nodes trees in the forest can have, `maxnodes`, and the number of trees to grow, `ntree`, were determined by an optimal search approach with respect to accuracy over a grid of candidate values $\{50, 60, 70, 80, 90, 100\}$ for `maxnodes` $\times$ $\{500, 800, 1000, 1300, 1500\}$ for `ntree`.

- The number of variables randomly sampled as candidates at each split, `mtry`, was optimally tuned using the `tuneRF` function in the `randomForest` package, where at each iteration, `mtry` was inflated (or deflated) by a value of `stepFactor = 1.2`, and the (relative) improvement in out-of-bag error had to be by `improve = 0.01` much for the search to continue.

For the evaluation of whichever model, we overall performed repeated nested cross-validation. Specifically, repeated cross-validation with 5 folds and 5 repeats was used in the inner loop for hyperparameter tuning, and repeated stratified cross-validation with 5 folds and 10 repeats was used in the outer loop for model evaluation. Eventually, we reported the distributions of the following evaluation measures over the 10 repetitions for the median values over the 5 folds: area under the receiver-operating characteristic (AUROC), area under the precision-recall curve (AUPRC) and Matthews correlation coefficient (MCC) (Chicco and Jurman, 2020), where the latter was based on a cut-off corresponding to the maximum sum of sensitivity and specificity.

For each outcome, we derived the top 25% (i.e., 8) of the features with the highest variable importance from the respective RF model. Here, variable importance was quantified by the median value of the mean decrease in accuracy from the respective distributions over the 10 repetitions for the median values over the 5 folds.

For each RF model, we additionally assessed statistical significance of the model performance and the suitability to detect a signal in the data using an empirical p-value based on a permutation test procedure. Specifically, we $M := 100$ times (i.e., repeatedly) randomly assigned the outcome values to the sets of feature values in the training data and computed M times the respective values of the evaluation measures for the shuffled data. For each evaluation measure, we then derived a corresponding empirical p-value by comparing the respective M medians over the 10 repetitions for the median values over the 5 folds based on the shuffled data to the respective median based on the original, non-shuffled composition of outcome and feature values.

Table S5: Specifications regarding the number of SCI individuals with complete data for all respective involved variables that were used for the RF analyses

Outcome	Number of SCI Individuals with Complete Data	Outcome Class Imbalance ("1" : "0")
Occurrence of a Clinically Relevant Degree of Overall Depressive Symptoms at Late Stage	148	$31 : 117 \approx 1 : 3.8$
Neurological Recovery wrt AIS	141	$38 : 103 \approx 1 : 2.7$
Neurological Recovery wrt UEMS	142	$52 : 90 \approx 1 : 1.7$
Neurological Recovery wrt TLT	142	$39 : 103 \approx 1 : 2.6$
Functional Recovery wrt SCIM-III Self-Care	144	$98 : 46 \approx 2.1 : 1$
Functional Recovery wrt SCIM-III RSM	144	$97 : 47 \approx 2.1 : 1$
Functional Recovery wrt SCIM-III Mobility	144	$88 : 56 \approx 1.6 : 1$

S5.2 Predicting the occurrence of a clinically relevant degree of overall depressive symptoms at late stage

Regarding the prediction of the occurrence of clinically relevant overall depressive symptoms in individuals with SCI at the late stage based on in total 33 features from the acute I stage, the RF model showed a mediocre predictive performance (median AUROC of 0.60, $p = 0.01$; median AUPRC of 0.22, $p = 0.29$; median MCC of 0.27, $p = 0.17$; Figure S11). The top 25% (i.e., 8) features with the highest median values regarding the mean decrease in accuracy were (Figure S12):
UEMS, TPP, TLT, SCIM-III Self-Care, Worthlessness, Tiredness, BinTotalBDI, and Indecisiveness.
That is, both SCI-specific (neurological and functional) and psychiatric variables were among the most important features. Notably, the demographic variables age and in particular gender were substantially less important (Figure S12). In contrast, BinTotalBDI was among the most important features, which is supported by the absence of a significant difference between the binarized total BDI-II scores at acute I and late stage (McNemar test: $p = 0.429$). Regarding the individual BDI-II items, SCI individuals with clinically relevant overall depressive symptoms at the late stage had significantly higher values than those without particularly for BDI-II items worthlessness (Wilcoxon rank sum test: $p = 0.0096$) and tiredness (Wilcoxon rank sum test: $p = 0.0114$), which were among the most important features. Our results align with recent observations (Gallo et al., 2023; Winter et al., 2024) indicating that machine learning or (even) deep learning approaches have some potential in distinguishing depressive individuals from HCs, but are also limited, possibly due to the well-known heterogeneity of depression pathology. Even though a generally known longitudinal relationship for psychiatric rating scales such as the BDI (Stahl et al., 2022) was confirmed (but not overly pronounced) here, also (at least some of the) SCI-specific features were important for the prediction of overall depressive symptoms at the late stage. In particular, we found that age and gender were no major predictors (and thus not included into the final RF model), aligning with mixed results from previous studies on their role as risk factors: some reported them as relevant (Arango-Lasprilla et al., 2011; Saunders et al., 2012), others not (Craig et al., 2014; Hoffman et al., 2011).

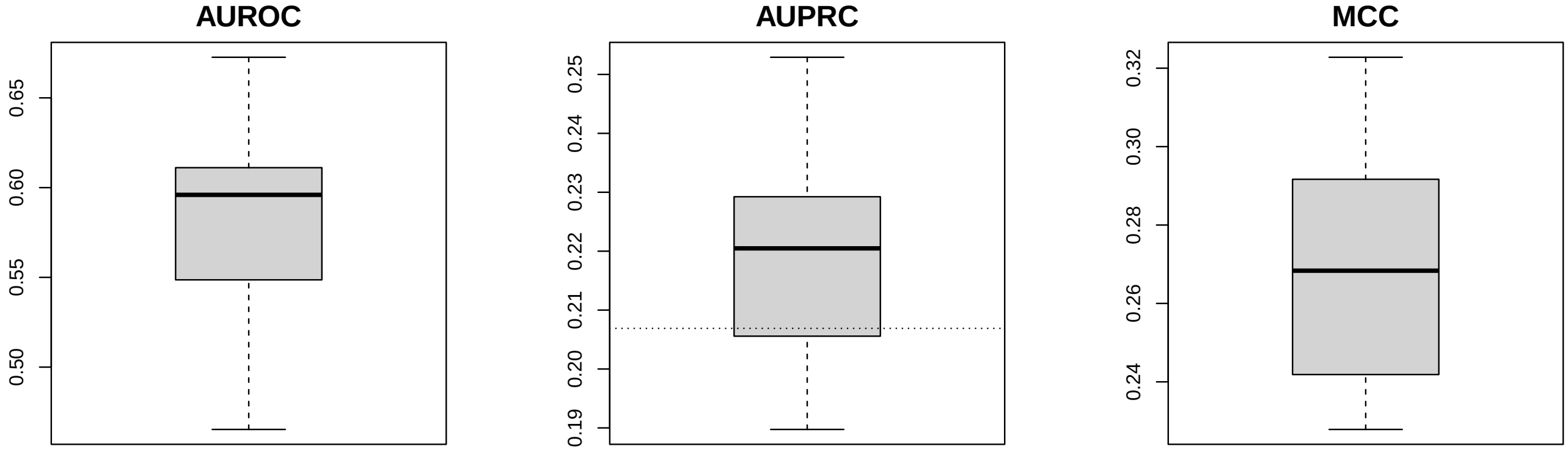

Figure S11: Boxplots illustrating the distributions of AUROC, AUPRC and MCC values for the RF model for the outcome of **the occurrence of a clinically relevant degree of overall depressive symptoms at late stage**, using features from acute I stage. The most important features (top 25% by variable importance) were UEMS, TPP, TLT, SCIM-III Self-Care, Worthlessness, Tiredness, BinTotalBDI, and Indecisiveness. The dotted horizontal line in the AUPRC plot indicates the median AUPRC corresponding to a baseline classifier determined by the ratio of positives and negatives.

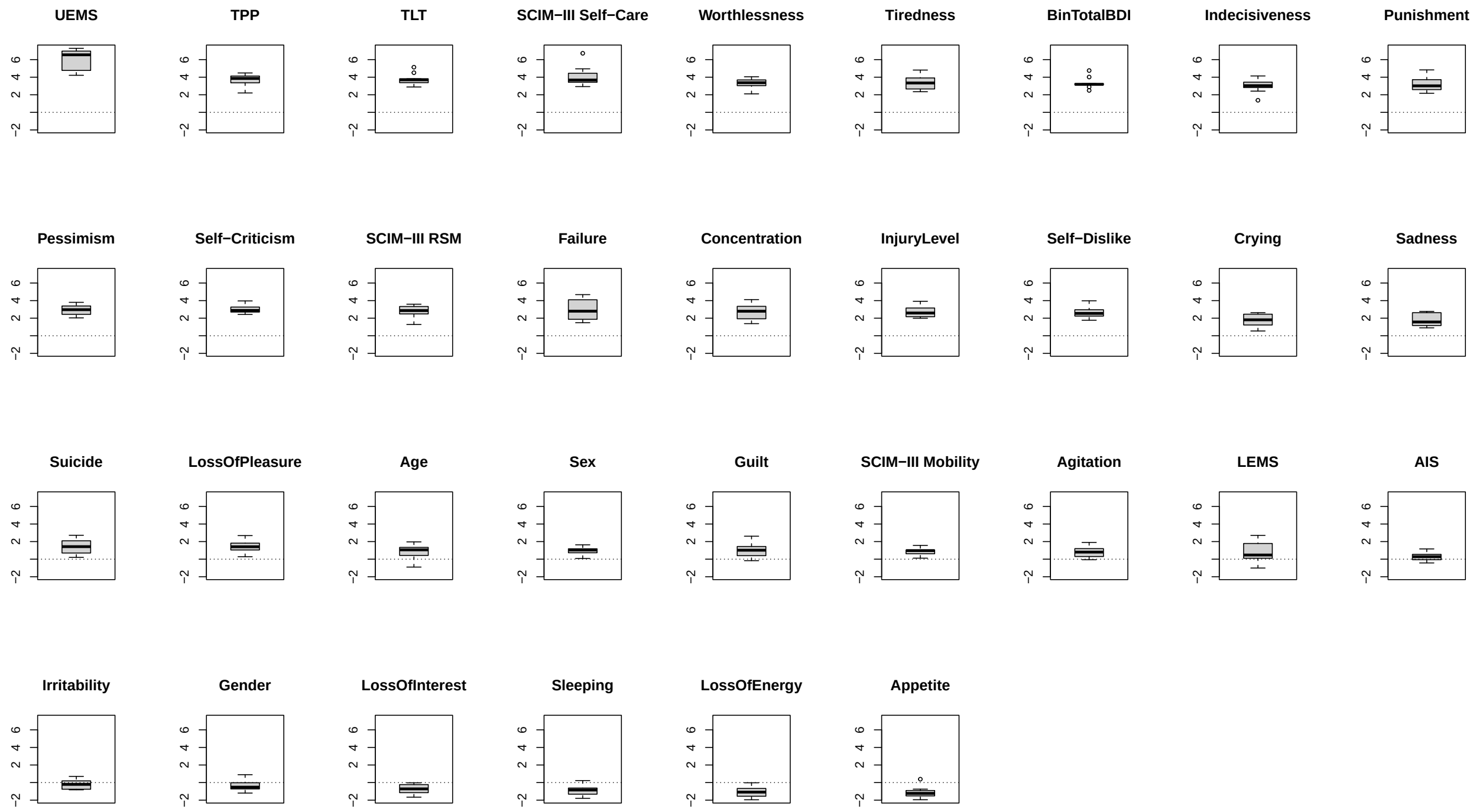

Figure S12: Boxplots illustrating the distributions of variable importance measured in terms of mean decrease accuracy for the RF model for the outcome of **the occurrence of a clinically relevant degree of overall depressive symptoms at late stage**, using features from acute I stage.

S5.3 Predicting neurological recovery

Regarding the prediction of neurological recovery with respect to different outcomes based on feature data from the acute I stage, the RF models overall performed well (e.g., for neurological recovery with respect to AIS: median AUROC of 0.82, $p < 0.01$; median AUPRC of 0.52, $p < 0.01$; median MCC of 0.58, $p < 0.01$) (Figures S13, S15 and S17). The respective top 25% (i.e., 8) features with the highest median values regarding the mean decrease in accuracy were:

- neurological recovery with respect to AIS (Figures S13 and S14): AIS, LEMS, SCIM-III Mobility, TLT, UEMS, SCIM-III RSM, SCIM-III Self-Care, and Sex,
- neurological recovery with respect to UEMS (Figures S15 and S16): UEMS, InjuryLevel, AIS, LEMS, TPP, SCIM-III Self-Care, TLT, and Agitation,
- neurological recovery with respect to TLT (Figures S17 and S18): LEMS, TPP, InjuryLevel, SCIM-III RSM, AIS, TLT, UEMS, and Sex.

That is, almost exclusively SCI-specific (neurological/functional) variables were among the most important features. Thus, neurological recovery appeared to be mostly determined by SCI-specific parameters and not related to the psychiatric or demographic parameters.

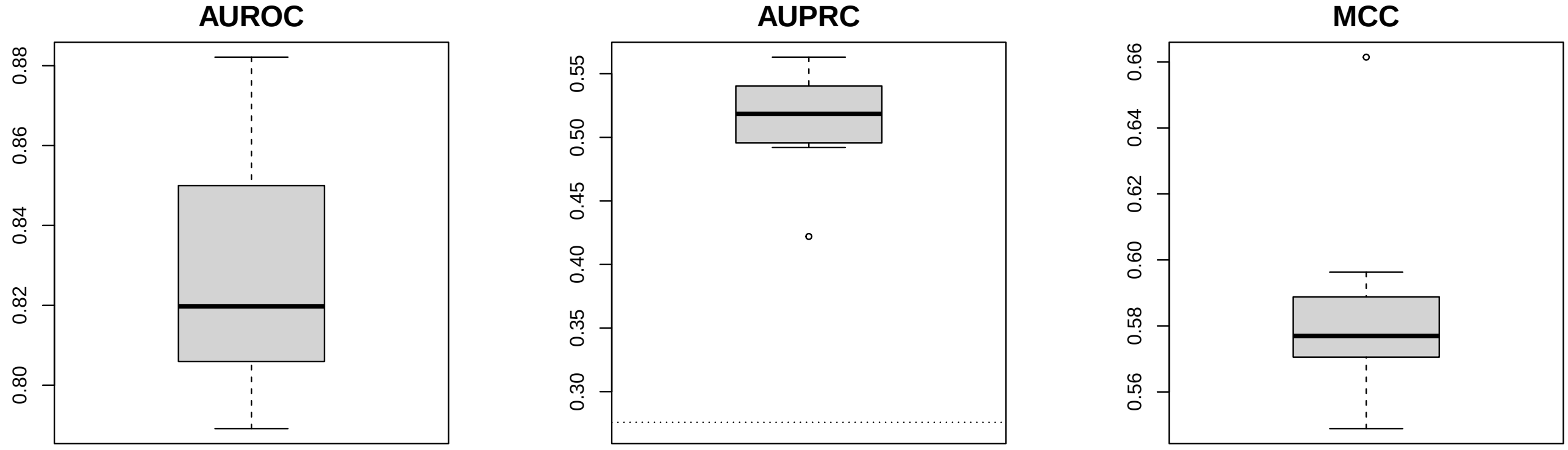

Figure S13: Boxplots illustrating the distributions of AUROC, AUPRC and MCC values for the RF model for the outcome of **neurological recovery with respect to AIS**, using features from acute I stage. The most important features (top 25% by variable importance) were AIS, LEMS, SCIM-III Mobility, TLT, UEMS, SCIM-III RSM, SCIM-III Self-Care, and Sex. The dotted horizontal line in the AUPRC plot indicates the median AUPRC corresponding to a baseline classifier determined by the ratio of positives and negatives.

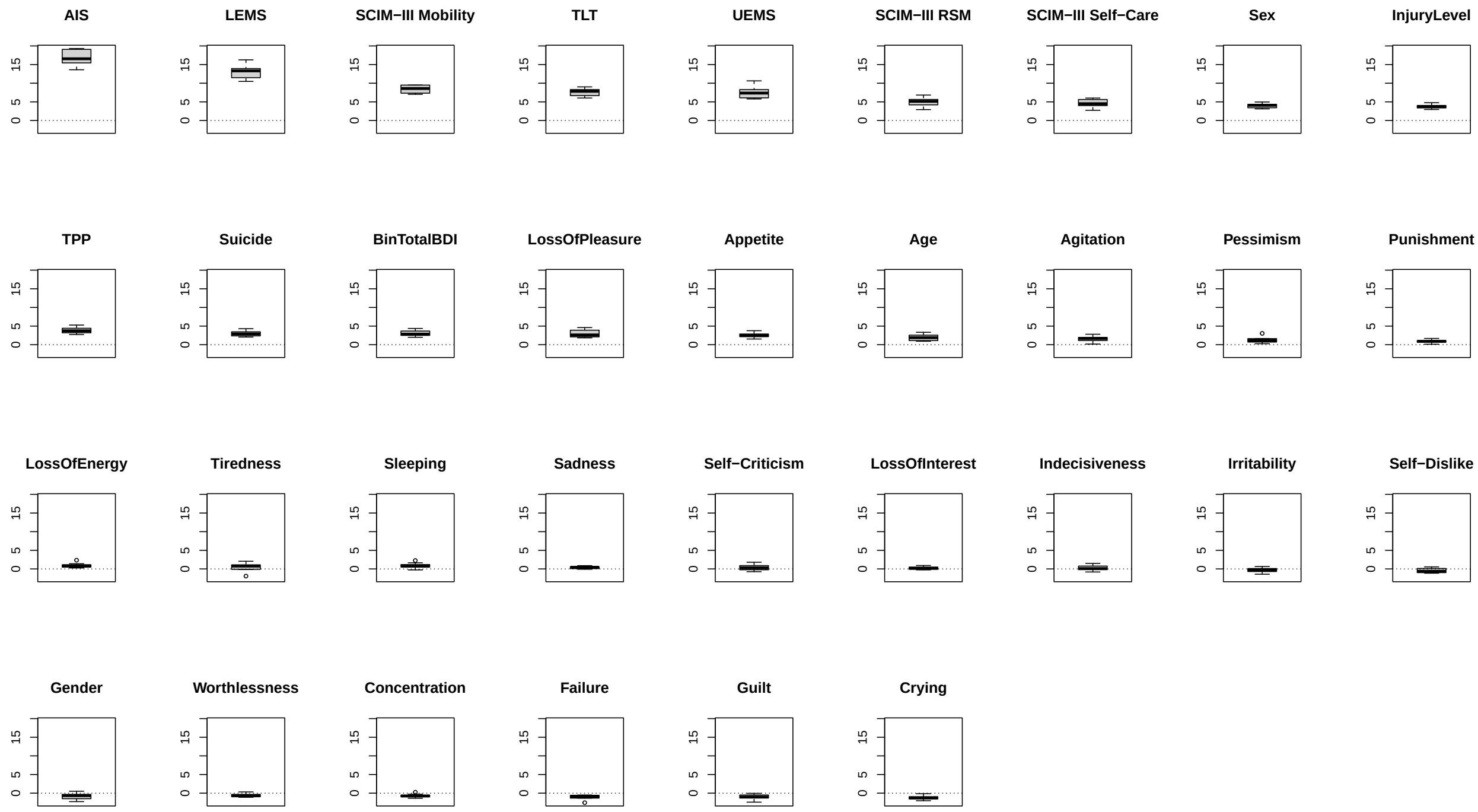

Figure S14: Boxplots illustrating the distributions of variable importance measured in terms of mean decrease accuracy for the RF model for the outcome of **neurological recovery with respect to AIS**, using features from acute I stage.

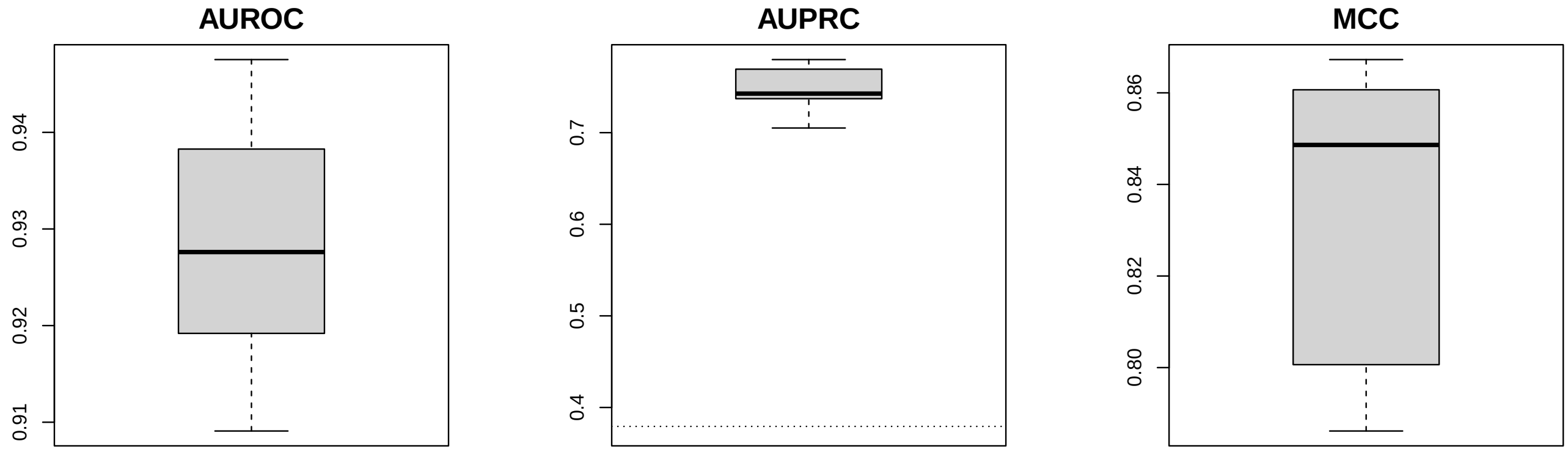

Figure S15: Boxplots illustrating the distributions of AUROC, AUPRC and MCC values for the RF model for the outcome of **neurological recovery with respect to UEMS**, using features from acute I stage. The most important features (top 25% by variable importance) were UEMS, InjuryLevel, AIS, LEMS, TPP, SCIM-III Self-Care, TLT, and Agitation. The dotted horizontal line in the AUPRC plot indicates the median AUPRC corresponding to a baseline classifier determined by the ratio of positives and negatives.

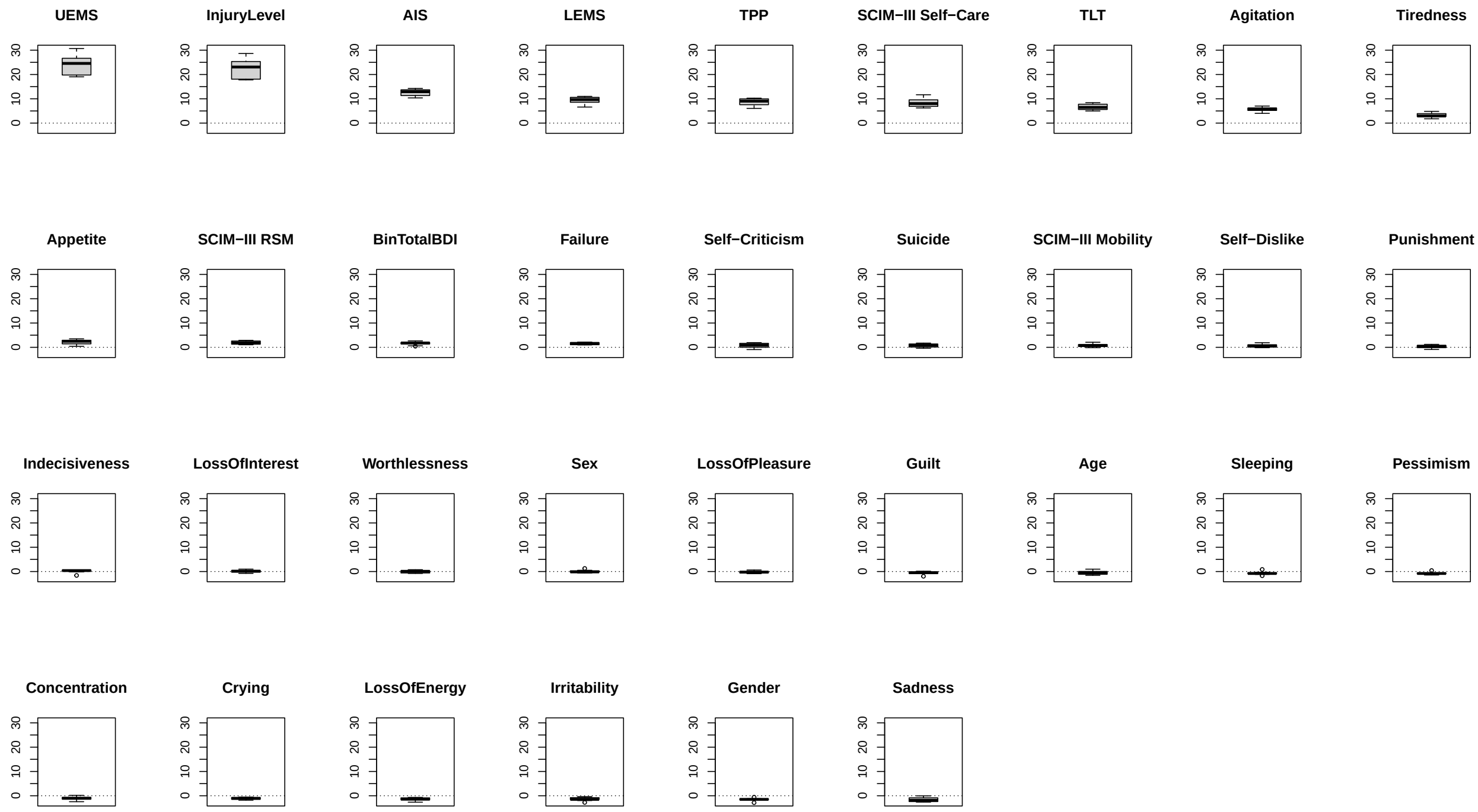

Figure S16: Boxplots illustrating the distributions of variable importance measured in terms of mean decrease accuracy for the RF model for the outcome of **neurological recovery with respect to UEMS**, using features from acute I stage.

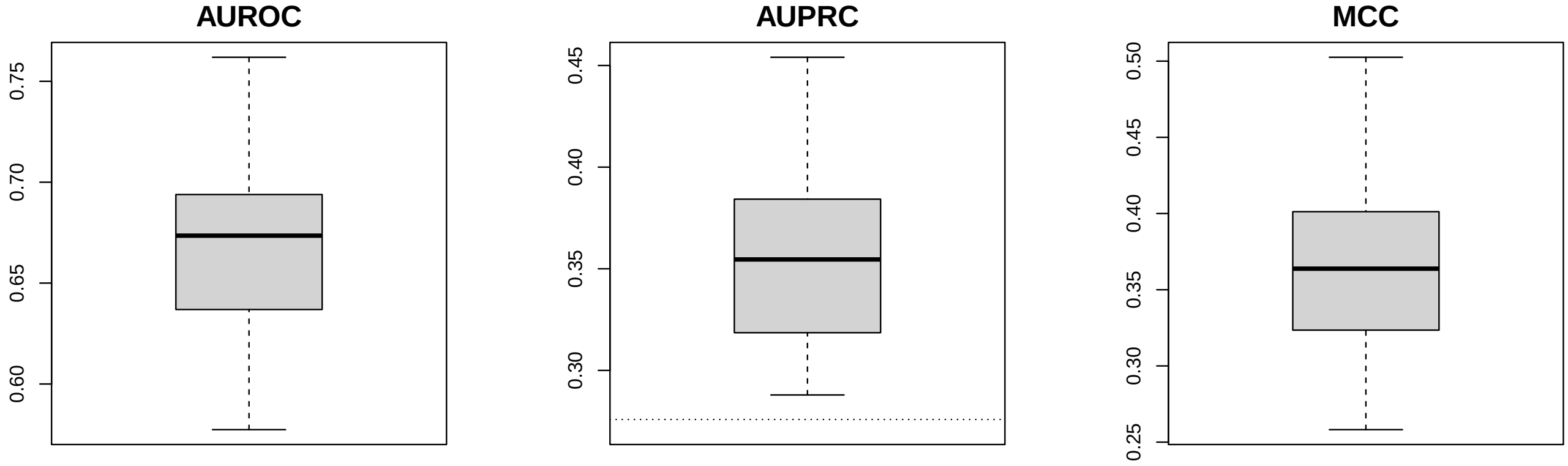

Figure S17: Boxplots illustrating the distributions of AUROC, AUPRC and MCC values for the RF model for the outcome of **neurological recovery with respect to TLT**, using features from acute I stage. The most important features (top 25% by variable importance) were LEMS, TPP, InjuryLevel, SCIM-III RSM, AIS, TLT, UEMS, and Sex. The dotted horizontal line in the AUPRC plot indicates the median AUPRC corresponding to a baseline classifier determined by the ratio of positives and negatives.

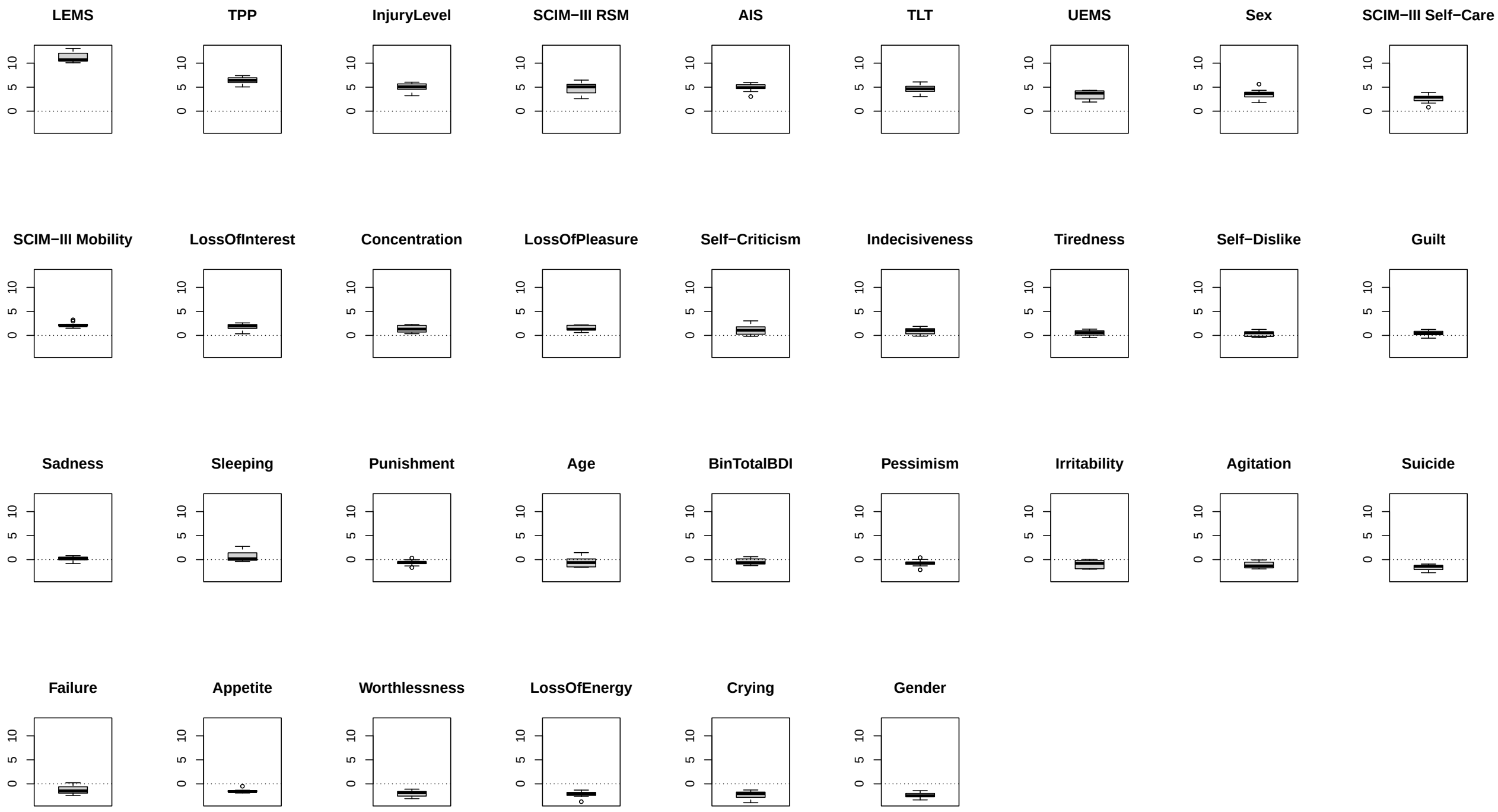

Figure S18: Boxplots illustrating the distributions of variable importance measured in terms of mean decrease accuracy for the RF model for the outcome of **neurological recovery with respect to TLT**, using features from acute I stage.

S5.4 Predicting functional recovery

Regarding the prediction of functional recovery with respect to different outcomes based on feature data from the acute I stage, the RF models overall performed well (e.g., for functional recovery with respect to SCIM-III Self-Care: median AUROC of 0.86, $p < 0.01$; median AUPRC of 0.84, $p < 0.01$; median MCC of 0.66, $p < 0.01$) (Figures S19, S21 and S23). The respective top 25% (i.e., 8) features with the highest median values regarding the mean decrease in accuracy were:

- functional recovery with respect to SCIM-III Self-Care (Figures S19 and S20): UEMS, SCIM-III Self-Care, TPP, SCIM-III RSM, SCIM-III Mobility, TLT, Age, and BinTotalBDI,
- functional recovery with respect to SCIM-III RSM (Figures S21 and S22): Age, SCIM-III RSM, UEMS, SCIM-III Self-Care, TLT, SCIM-III Mobility, TPP, and Irritability,
- functional recovery with respect to SCIM-III Mobility (Figures S23 and S24): Age, UEMS, LEMS, TPP, AIS, SCIM-III Mobility, TLT, and SCIM-III Self-Care.

That is, virtually exclusively SCI-specific (predominantly functional) features as well as age were among the most important features. Overall, functional recovery appeared to be mostly determined by SCI-specific parameters, as well as age, and not related to the psychiatric parameters.

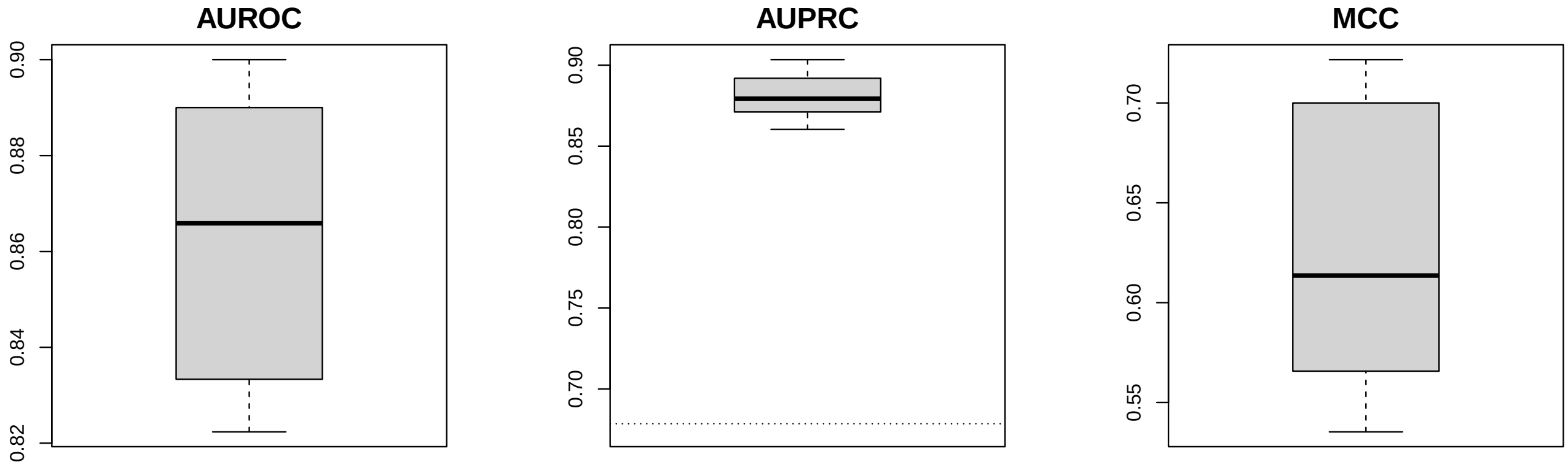

Figure S19: Boxplots illustrating the distributions of AUROC, AUPRC and MCC values for the RF model for the outcome of **functional recovery with respect to SCIM-III Self-Care**, using features from acute I stage. The most important features (top 25% by variable importance) were UEMS, SCIM-III Self-Care, TPP, SCIM-III RSM, SCIM-III Mobility, TLT, Age, and BinTotalBDI. The dotted horizontal line in the AUPRC plot indicates the median AUPRC corresponding to a baseline classifier determined by the ratio of positives and negatives.

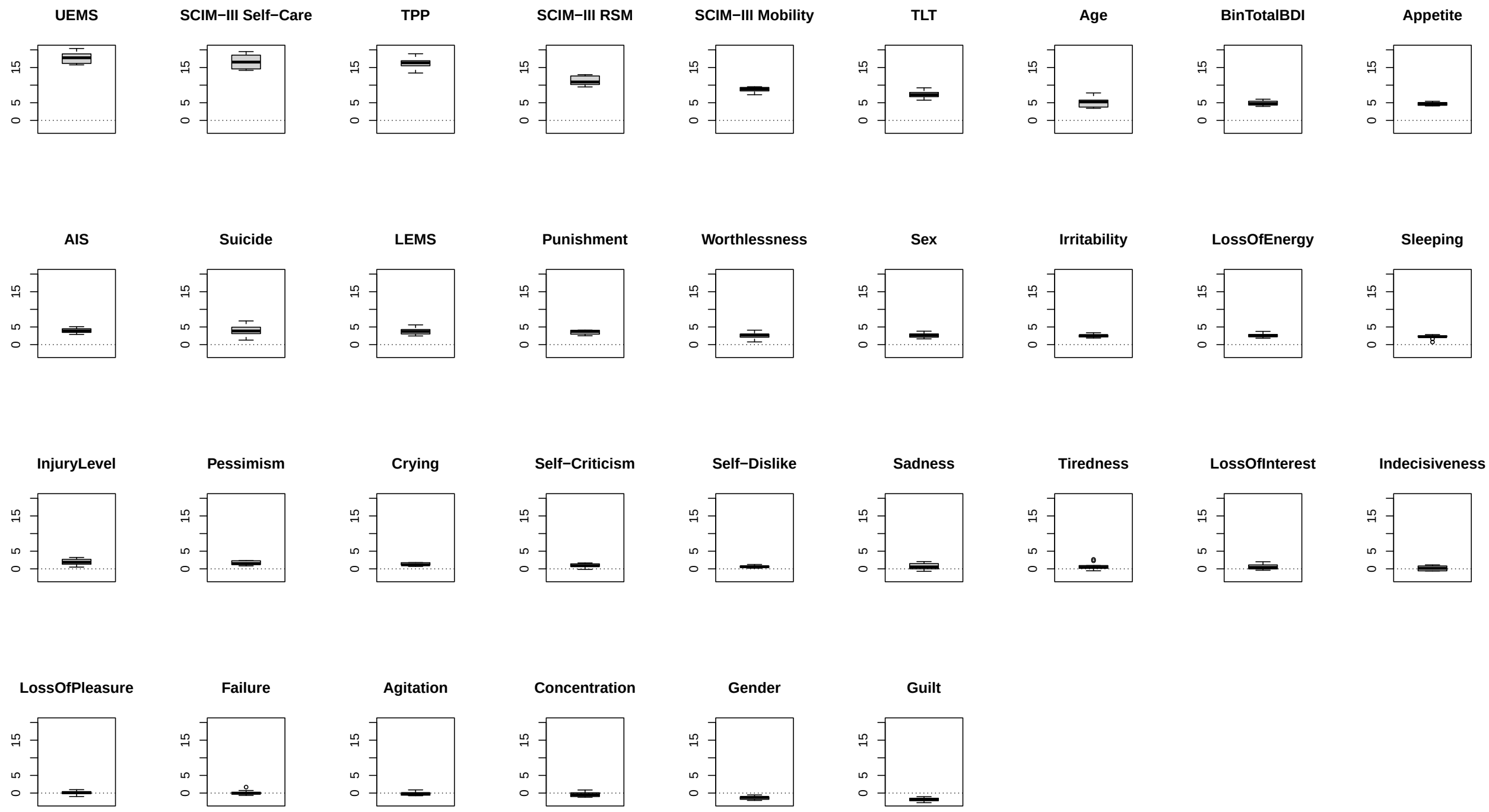

Figure S20: Boxplots illustrating the distributions of variable importance measured in terms of mean decrease accuracy for the RF model for the outcome of **functional recovery with respect to SCIM-III Self-Care**, using features from acute I stage.

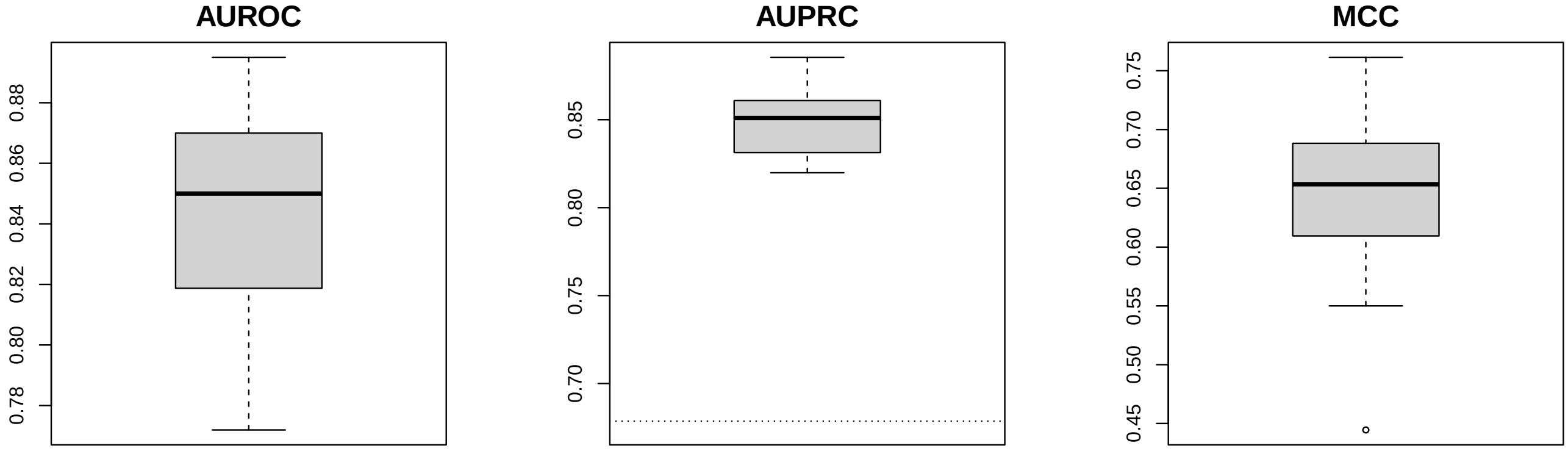

Figure S21: Boxplots illustrating the distributions of AUROC, AUPRC and MCC values for the RF model for the outcome of **functional recovery with respect to SCIM-III RSM**, using features from acute I stage. The most important features (top 25% by variable importance) were Age, SCIM-III RSM, UEMS, SCIM-III Self-Care, TLT, SCIM-III Mobility, TPP, and Irritability. The dotted horizontal line in the AUPRC plot indicates the median AUPRC corresponding to a baseline classifier determined by the ratio of positives and negatives.

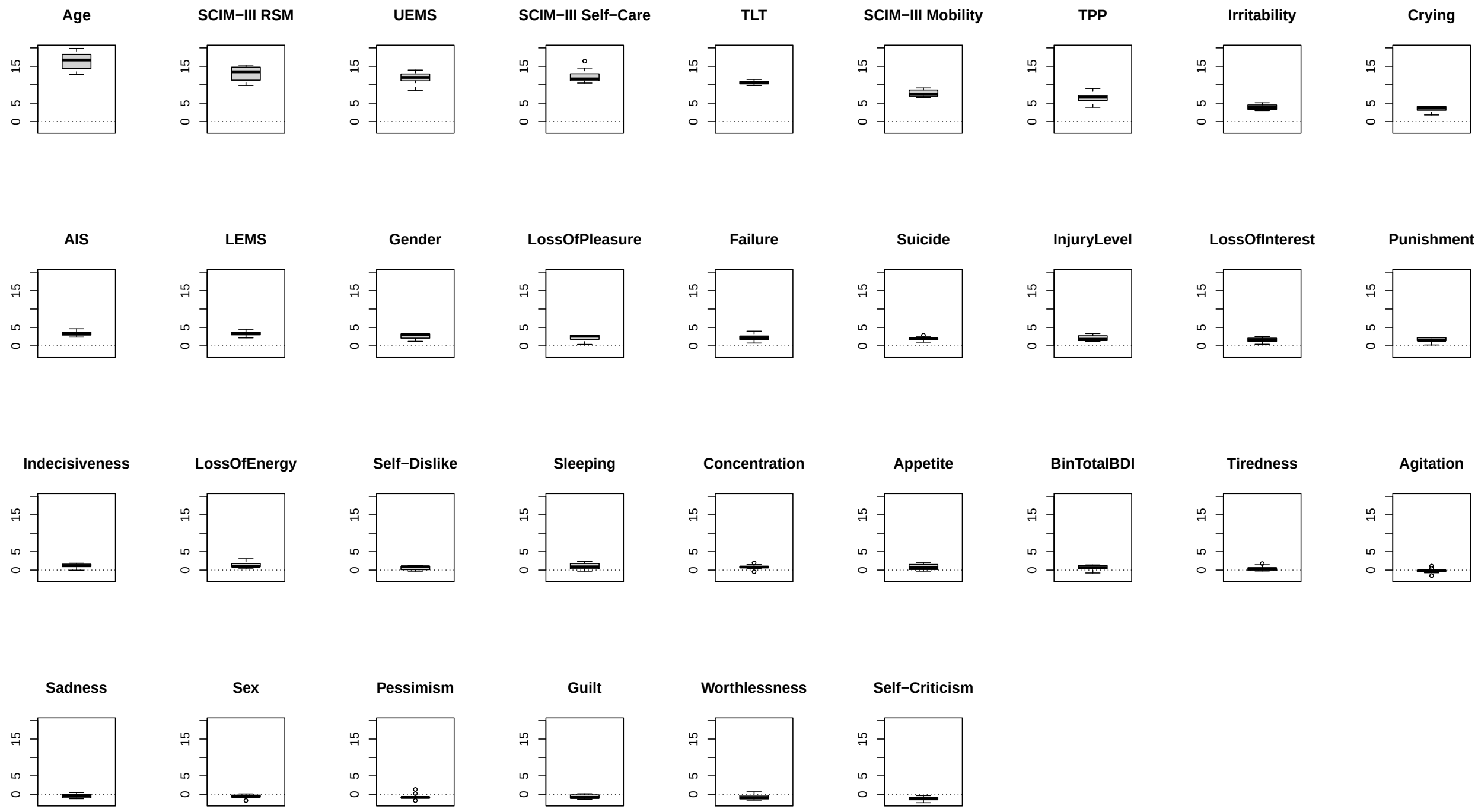

Figure S22: Boxplots illustrating the distributions of variable importance measured in terms of mean decrease accuracy for the RF model for the outcome of **functional recovery with respect to SCIM-III RSM**, using features from acute I stage.

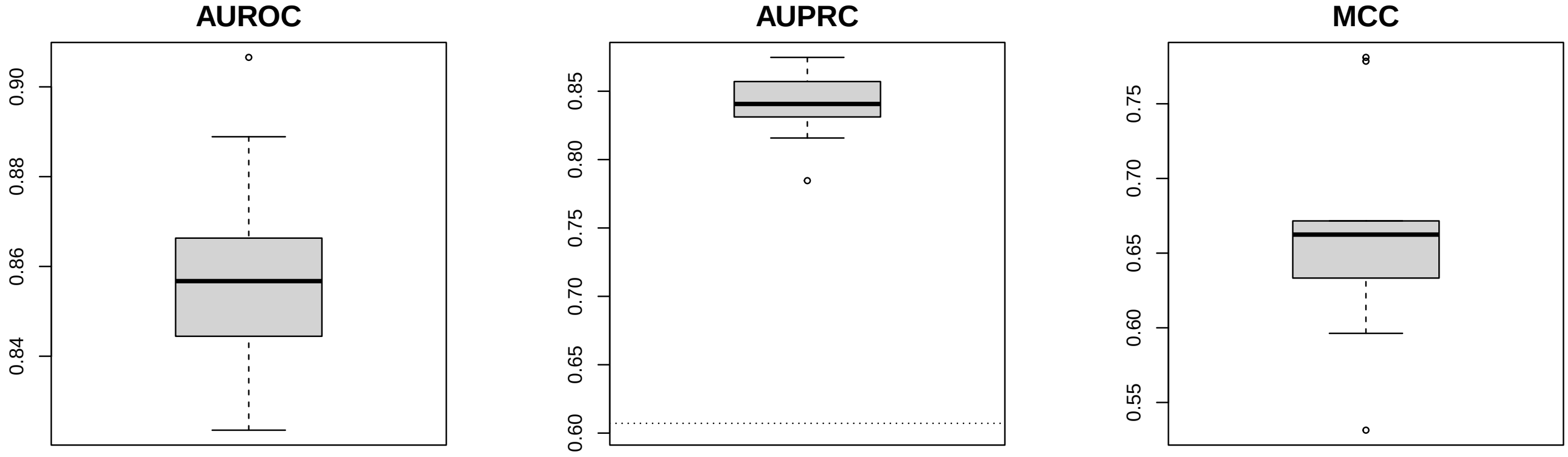

Figure S23: Boxplots illustrating the distributions of AUROC, AUPRC and MCC values for the RF model for the outcome of **functional recovery with respect to SCIM-III Mobility**, using features from acute I stage. The most important features (top 25% by variable importance) were Age, UEMS, LEMS, TPP, AIS, SCIM-III Mobility, TLT, and SCIM-III Self-Care. The dotted horizontal line in the AUPRC plot indicates the median AUPRC corresponding to a baseline classifier determined by the ratio of positives and negatives.

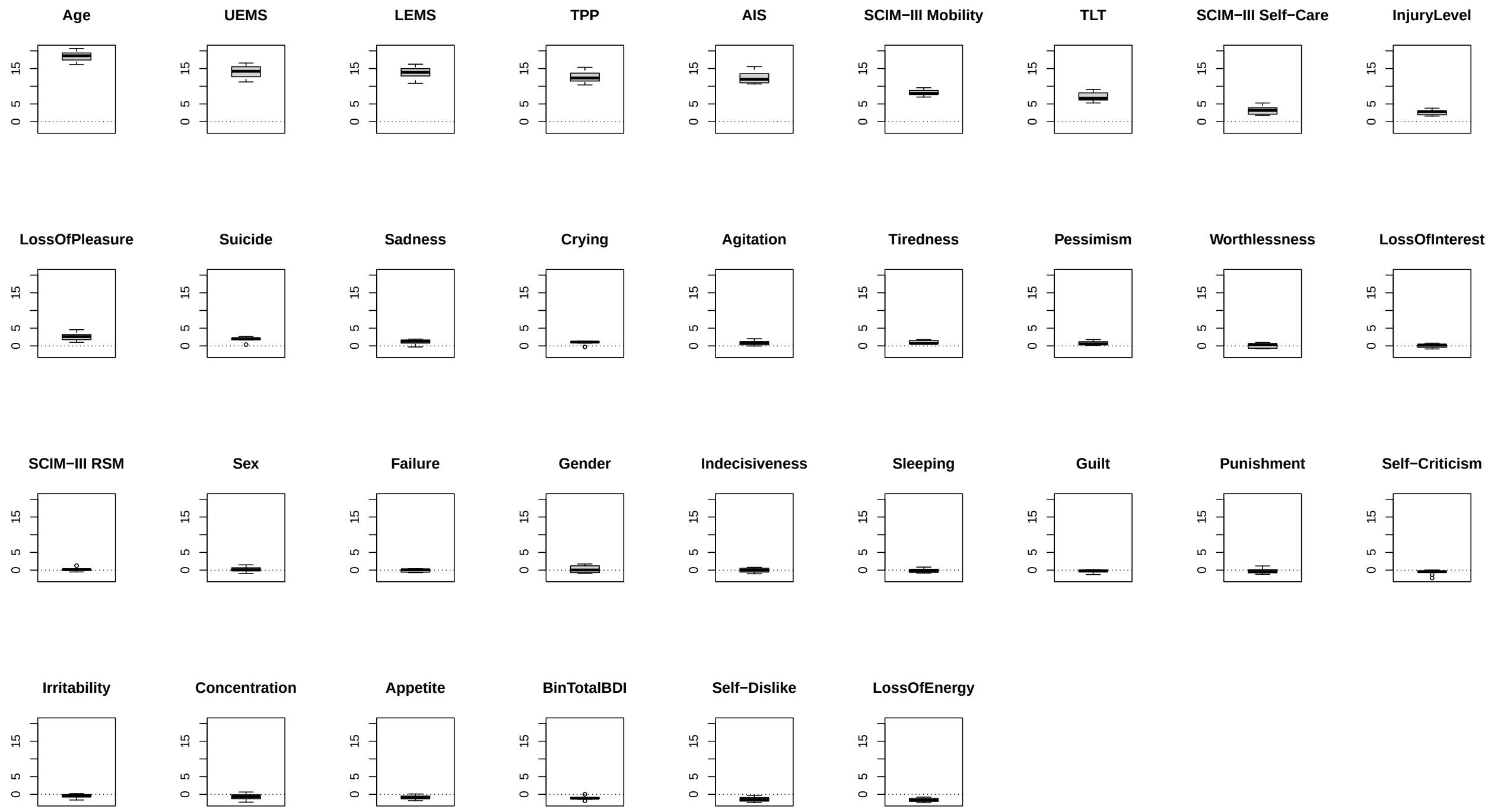

Figure S24: Boxplots illustrating the distributions of variable importance measured in terms of mean decrease accuracy for the RF model for the outcome of **functional recovery with respect to SCIM-III Mobility**, using features from acute I stage.

S6 Supplementary results: SCI cohort definition and selection

The focus of our analyses was on evaluating the development of depressive symptoms in SCI. Consequently, we included only participants with complete BDI-II data at both the acute I and late stages. This reduced the SCI cohort from originally 227 to $N = 165$ individuals. To assess the potential impact of attrition on our findings, we compared baseline (i.e., acute I) psychiatric, demographic, neurological, and functional characteristics between SCI individuals who were excluded from the final analyses ($N_{\text{excl}} = 62$) and those who were included ($N = 165$) in Table S6.

These comparisons did not reveal statistically significant differences in any of the examined variables. Although the absence of detectable baseline differences cannot rule out all forms of selection bias, it suggests that participant exclusion was unlikely to have introduced substantial systematic selection effects or biases, thereby supporting the representativeness of the analyzed SCI cohort.

Table S6: Comparisons of baseline (i.e., acute I) psychiatric, demographic neurological, and functional characteristics between SCI individuals who were excluded from the final analyses ($N_{\text{excl}} = 62$) and those who were included ($N = 165$)

Characteristics of SCI Individuals at Acute I Stage

Variable	N	**Overall** N = 227	Excluded/Included		p-value[1]
			**Excluded** N = 62	**Included** N = 165	
**BDI01 Sadness, Mean (SD),Median (Q1-Q3)**	196	0.56 (0.59),1.00 (0.00-1.00)	0.42 (0.56),0.00 (0.00-1.00)	0.58 (0.60),1.00 (0.00-1.00)	0.15
Missing		31	31	0	
**BDI02 Pessimism, Mean (SD),Median (Q1-Q3)**	196	0.48 (0.59),0.00 (0.00-1.00)	0.45 (0.51),0.00 (0.00-1.00)	0.48 (0.60),0.00 (0.00-1.00)	0.97
Missing		31	31	0	
**BDI03 Failure, Mean (SD),Median (Q1-Q3)**	196	0.19 (0.51),0.00 (0.00-0.00)	0.29 (0.69),0.00 (0.00-0.00)	0.18 (0.47),0.00 (0.00-0.00)	0.41
Missing		31	31	0	
**BDI04 Loss Of Pleasure, Mean (SD),Median (Q1-Q3)**	196	0.71 (0.59),1.00 (0.00-1.00)	0.68 (0.65),1.00 (0.00-1.00)	0.72 (0.58),1.00 (0.00-1.00)	0.62
Missing		31	31	0	
**BDI05 Guilt, Mean (SD),Median (Q1-Q3)**	196	0.26 (0.57),0.00 (0.00-0.00)	0.23 (0.43),0.00 (0.00-0.00)	0.27 (0.60),0.00 (0.00-0.00)	0.90
Missing		31	31	0	
**BDI06 Punishment, Mean (SD),Median (Q1-Q3)**	195	0.21 (0.60),0.00 (0.00-0.00)	0.23 (0.77),0.00 (0.00-0.00)	0.20 (0.57),0.00 (0.00-0.00)	0.58
Missing		32	32	0	
**BDI07 Self-Dislike, Mean (SD),Median (Q1-Q3)**	195	0.17 (0.45),0.00 (0.00-0.00)	0.20 (0.55),0.00 (0.00-0.00)	0.16 (0.43),0.00 (0.00-0.00)	>0.99
Missing		32	32	0	
**BDI08 Self-Criticism, Mean (SD),Median (Q1-Q3)**	195	0.50 (0.62),0.00 (0.00-1.00)	0.43 (0.73),0.00 (0.00-1.00)	0.51 (0.60),0.00 (0.00-1.00)	0.26
Missing		32	32	0	
**BDI09 Suicide, Mean (SD),Median (Q1-Q3)**	195	0.10 (0.30),0.00 (0.00-0.00)	0.10 (0.31),0.00 (0.00-0.00)	0.10 (0.30),0.00 (0.00-0.00)	0.96
Missing		32	32	0	
**BDI10 Crying, Mean (SD),Median (Q1-Q3)**	194	0.48 (0.66),0.00 (0.00-1.00)	0.55 (0.69),0.00 (0.00-1.00)	0.47 (0.66),0.00 (0.00-1.00)	0.50
Missing		33	33	0	
**BDI11 Agitation, Mean (SD),Median (Q1-Q3)**	196	0.45 (0.63),0.00 (0.00-1.00)	0.61 (0.80),0.00 (0.00-1.00)	0.42 (0.59),0.00 (0.00-1.00)	0.26
Missing		31	31	0	
**BDI12 Loss Of Interest, Mean (SD),Median (Q1-Q3)**	196	0.24 (0.50),0.00 (0.00-0.00)	0.29 (0.46),0.00 (0.00-1.00)	0.23 (0.51),0.00 (0.00-0.00)	0.29
Missing		31	31	0	
**BDI13 Indecisiveness, Mean (SD),Median (Q1-Q3)**	195	0.45 (0.61),0.00 (0.00-1.00)	0.43 (0.68),0.00 (0.00-1.00)	0.45 (0.60),0.00 (0.00-1.00)	0.71
Missing		32	32	0	

Table S6: Continued

Variable	N	Overall N = 227	Excluded/Included		p-value[1]
			**Excluded** N = 62	**Included** N = 165	
**BDI14 Worthlessness, Mean (SD),Median (Q1-Q3)**	195	0.46 (0.59),0.00 (0.00-1.00)	0.43 (0.68),0.00 (0.00-1.00)	0.47 (0.57),0.00 (0.00-1.00)	0.52
Missing		32	32	0	
**BDI15 Loss Of Energy, Mean (SD),Median (Q1-Q3)**	195	0.82 (0.64),1.00 (0.00-1.00)	0.73 (0.58),1.00 (0.00-1.00)	0.83 (0.65),1.00 (0.00-1.00)	0.52
Missing		32	32	0	
**BDI16 Sleeping, Mean (SD),Median (Q1-Q3)**	195	0.95 (0.80),1.00 (0.00-1.00)	1.13 (0.97),1.00 (0.00-2.00)	0.92 (0.76),1.00 (0.00-1.00)	0.30
Missing		32	32	0	
**BDI17 Irritability, Mean (SD),Median (Q1-Q3)**	196	0.23 (0.47),0.00 (0.00-0.00)	0.23 (0.43),0.00 (0.00-0.00)	0.24 (0.48),0.00 (0.00-0.00)	0.95
Missing		31	31	0	
**BDI18 Appetite, Mean (SD),Median (Q1-Q3)**	196	0.85 (0.90),1.00 (0.00-1.00)	0.81 (0.98),1.00 (0.00-1.00)	0.85 (0.89),1.00 (0.00-1.00)	0.57
Missing		31	31	0	
**BDI19 Concentration, Mean (SD),Median (Q1-Q3)**	196	0.36 (0.59),0.00 (0.00-1.00)	0.45 (0.68),0.00 (0.00-1.00)	0.35 (0.57),0.00 (0.00-1.00)	0.46
Missing		31	31	0	
**BDI20 Tiredness, Mean (SD),Median (Q1-Q3)**	196	0.90 (0.61),1.00 (1.00-1.00)	0.81 (0.48),1.00 (1.00-1.00)	0.92 (0.63),1.00 (1.00-1.00)	0.45
Missing		31	31	0	
**BDI21 Sex, Mean (SD),Median (Q1-Q3)**	190	0.78 (1.02),0.00 (0.00-1.00)	0.64 (1.08),0.00 (0.00-1.00)	0.80 (1.01),0.00 (0.00-1.00)	0.25
Missing		37	37	0	
**Total BDI-II Score, Mean (SD),Median (Q1-Q3)**	186	10 (7),9 (5-14)	9 (6),8 (5-14)	10 (7),10 (5-13)	0.57
Missing		41	41	0	
**Gender, n (%)**	227				0.86
Female		56 (24.7%)	16 (25.8%)	40 (24.2%)	
Male		171 (75.3%)	46 (74.2%)	125 (75.8%)	
**Age at DOI, yr, Mean (SD),Median (Q1-Q3)**	227	47 (19),48 (30-64)	49 (20),51 (31-66)	47 (19),48 (30-64)	0.62
**Cause of Injury, n (%)**	227				>0.99
Ischemic		15 (6.6%)	4 (6.5%)	11 (6.7%)	
Traumatic		212 (93.4%)	58 (93.5%)	154 (93.3%)	
**Injury Level, n (%)**	227				0.26
Cervical		125 (55.1%)	35 (56.5%)	90 (54.5%)	
Lumbar		25 (11.0%)	4 (6.5%)	21 (12.7%)	
Not Defined		1 (0.4%)	1 (1.6%)	0 (0.0%)	

Table S6: Continued

Variable	N	**Overall** N = 227	Excluded/Included<br>**Excluded** N = 62	 **Included** N = 165	p-value[1]
Thoracic		76 (33.5%)	22 (35.5%)	54 (32.7%)	
**AIS, n (%)**	208				0.16
A		82 (36.1%)	23 (37.1%)	59 (35.8%)	
B		25 (11.0%)	4 (6.5%)	21 (12.7%)	
C		25 (11.0%)	3 (4.8%)	22 (13.3%)	
D		76 (33.5%)	24 (38.7%)	52 (31.5%)	
Missing		19 (8.4%)	8 (12.9%)	11 (6.7%)	
**UEMS, Mean (SD),Median (Q1-Q3)**	208	37 (17),45 (25-50)	38 (17),48 (31-50)	37 (16),44 (24-50)	0.73
Missing		19	8	11	
**LEMS, Mean (SD),Median (Q1-Q3)**	208	17 (20),3 (0-38)	19 (21),6 (0-40)	16 (20),3 (0-37)	0.76
Missing		19	8	11	
**TLT, Mean (SD),Median (Q1-Q3)**	201	72 (29),76 (54-97)	74 (32),75 (53-103)	72 (28),78 (57-93)	0.46
Missing		26	10	16	
**TPP, Mean (SD),Median (Q1-Q3)**	204	61 (31),62 (38-86)	64 (30),67 (38-94)	60 (31),60 (38-86)	0.37
Missing		23	10	13	
**SCIM-III Self-Care, Mean (SD),Median (Q1-Q3)**	226	7 (6),6 (1-10)	7 (6),6 (1-9)	7 (6),6 (0-11)	0.91
Missing		1	0	1	
**SCIM-III RSM, Mean (SD),Median (Q1-Q3)**	226	14 (11),11 (10-18)	14 (11),13 (6-15)	15 (11),11 (10-18)	0.56
Missing		1	0	1	
**SCIM-III Mobility, Mean (SD),Median (Q1-Q3)**	226	7 (10),3 (0-10)	7 (11),3 (0-10)	7 (9),3 (0-10)	0.72
Missing		1	0	1	

[1]Wilcoxon rank sum test; Fisher's exact test; Fisher's Exact Test for Count Data with simulated p-value (based on 2000 replicates)

S7 Supplementary methods and results: Cross-cohort comparisons based on PCA using polychoric correlations

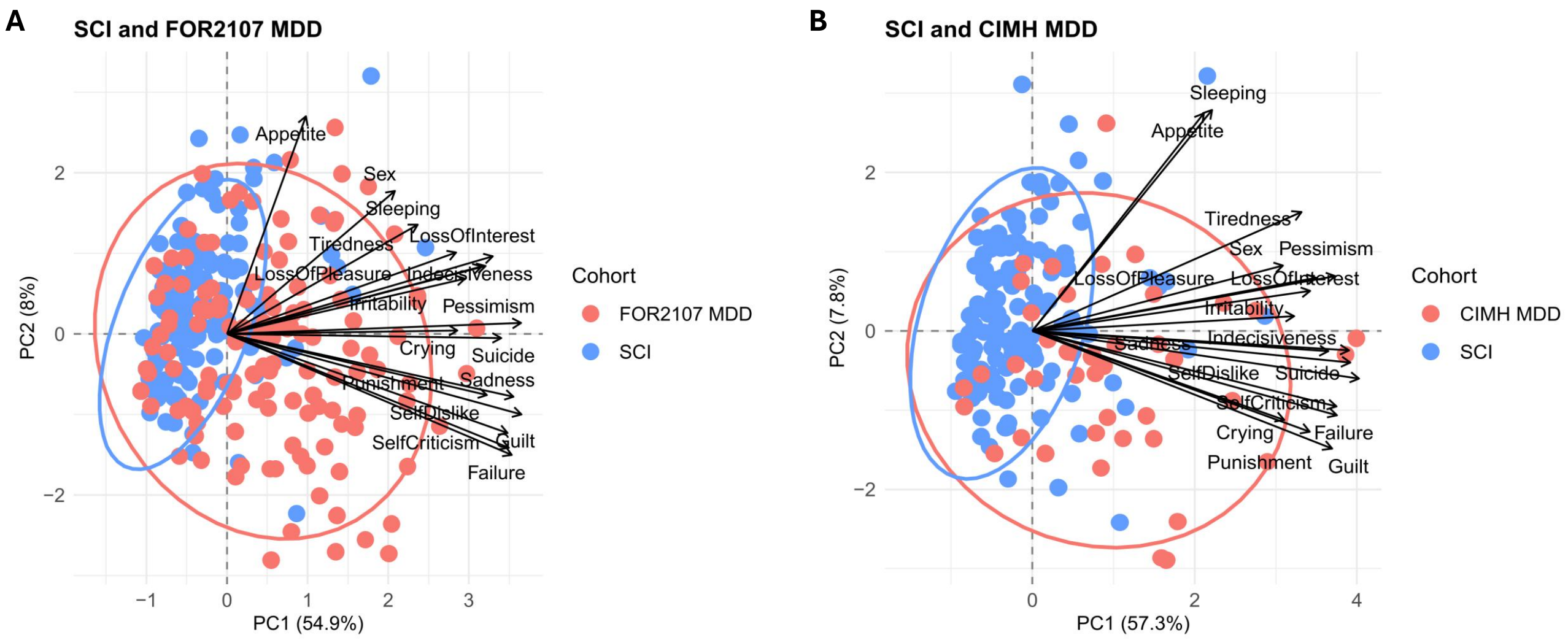

Figure S25: PCA biplots based on polychoric correlations to compare BDI item profiles of the individuals with SCI to those of individuals with MDD diagnosis from the (A) FOR2107 cohort and (B) CIMH cohort, respectively. Here, BDI refers to BDI-I/II Synchronized.

To account more explictly for the ordinal nature of the BDI data, we additionally performed PCA based on polychoric correlations (rather than standard PCA using Pearson correlations, which assumes interval-scaled data). This analysis was implemented using the principal() function in the R package `psych` (Revelle, 2026), and was applied for cross-cohort comparisons without explicit adjustment for age and gender.
Corresponding results comparing the SCI cohort with the FOR2107 MDD and CIMH MDD cohorts, respectively, with respect to the BDI-I/II Synchronized, are shown in Figure S25. Overall, the findings were consistent with those obtained from the standard PCA-based analyses (main text: Figure 5 and Results section; Sections S4.2 and S4.3), indicating that the main results are robust to the use of a polychoric correlation structure.

References

J. C. Arango-Lasprilla, J. M. Ketchum, A. Starkweather, E. Nicholls, and A. R. Wilk. Factors predicting depression among persons with spinal cord injury 1 to 5 years post injury. *NeuroRehabilitation*, 29:9–21, 2011.

L. Breiman. Random forests. *Machine Learning*, 45:5–32, 2001.

D. Chicco and G. Jurman. The advantages of the Matthews correlation coefficient (MCC) over F1 score and accuracy in binary classification evaluation. *BMC Genomics*, 21:6, 2020.

V. Corallo, M. Torre, G. Ferrara, F. Guerra, G. Nicosia, E. Romanelli, A. Lopopolo, M. P. Onesta, P. Fiore, R. Falcone, J. Bonavita, M. Molinari, and G. Scivoletto. What do spinal cord injury patients think of their improvement? A study of the minimal clinically important difference of the Spinal Cord Independence Measure III. *European Journal of Physical and Rehabilitation Medicine*, 53:508–515, 2017.

A. Craig, D. Rodrigues, Y. Tran, R. Guest, R. Bartrop, and J. Middleton. Developing an algorithm capable of discriminating depressed mood in people with spinal cord injury. *Spinal Cord*, 52:413–416, 2014.

S. Gallo, A. El-Gazzar, P. Zhutovsky, R. M. Thomas, N. Javaheripour, M. Li, L. Bartova, D. Bathula, U. Dannlowski, C. Davey, et al. Functional connectivity signatures of major depressive disorder: machine learning analysis of two multicenter neuroimaging studies. *Molecular Psychiatry*, 28:3013–3022, 2023.

J. M. Hoffman, C. H. Bombardier, D. E. Graves, C. Z. Kalpakjian, and J. S. Krause. A longitudinal study of depression from 1 to 5 years after spinal cord injury. *Archives of Physical Medicine and Rehabilitation*, 92:411–418, 2011.

M. Itzkovich, I. Gelernter, F. Biering-Sorensen, C. Weeks, M. T. Laramee, B. C. Craven, M. Tonack, S. L. Hitzig, E. Glaser, G. Zeilig, et al. The Spinal Cord Independence Measure (SCIM) version III: reliability and validity in a multi-center international study. *Disability and Rehabilitation*, 29: 1926–1933, 2007.

T. Kircher, M. Wöhr, I. Nenadic, R. Schwarting, G. Schratt, J. Alferink, C. Culmsee, H. Garn, T. Hahn, B. Müller-Myhsok, A. Dempfle, M. Hahmann, A. Jansen, P. Pfefferle, H. Renz, M. Rietschel, S. H. Witt, M. Nöthen, A. Krug, and U. Dannlowski. Neurobiology of the major psychoses: a translational perspective on brain structure and function - the FOR2107 consortium. *European Archives of Psychiatry and Clinical Neuroscience*, 269:949–962, 2019.

S. C. Kirshblum, S. P. Burns, F. Biering-Sorensen, W. Donovan, D. E. Graves, A. Jha, M. Johansen, L. Jones, A. Krassioukov, M. J. Mulcahey, M. Schmidt-Read, and W. Waring. International standards for neurological classification of spinal cord injury (revised 2011). *The Journal of Spinal Cord Medicine*, 34:535–546, 2011.

A. Liaw and M. Wiener. Classification and regression by randomForest. *R News*, 2:18–22, 2002.

W. Revelle. *psych: Procedures for Psychological, Psychometric, and Personality Research*. Comprehensive R Archive Network (CRAN), 2026. URL `https://CRAN.R-project.org/package=psych`. R package version 2.6.4.

T. T. Roberts, G. R. Leonard, and D. J. Cepela. Classifications In Brief: American Spinal Injury Association (ASIA) Impairment Scale. *Clinical Orthopaedics and Related Research*, 475:1499–1504, 2017.

R. Rupp, F. Biering-Sørensen, S. P. Burns, D. E. Graves, J. Guest, L. Jones, M. Schmidt Read, G. M. Rodriguez, C. Schuld, K. E. Tansey, K. Walden, and S. Kirshblum. International Standards for Neurological Classification of Spinal Cord Injury: Revised 2019. *Topics in Spinal Cord Injury Rehabilitation*, 27:1–22, 2021.

L. L. Saunders, J. S. Krause, and K. L. Focht. A longitudinal study of depression in survivors of spinal cord injury. *Spinal Cord*, 50:72–77, 2012.

K. Schwarz, C. Moessnang, J. I. Schweiger, S. Baumeister, M. M. Plichta, D. Brandeis, T. Banaschewski, C. Wackerhagen, S. Erk, H. Walter, H. Tost, and A. Meyer-Lindenberg. Transdiagnostic prediction of affective, cognitive, and social function through brain reward anticipation in schizophrenia, bipolar disorder, major depression, and autism spectrum diagnoses. *Schizophrenia Bulletin*, 46:592–602, 2020.

G. Scivoletto, F. Tamburella, L. Laurenza, and M. Molinari. Distribution-based estimates of clinically significant changes in the international standards for neurological classification of spinal cord injury motor and sensory scores. *European Journal of Physical and Rehabilitation Medicine*, 49:373–384, 2013.

K. Stahl, K. Adorjan, H. Anderson-Schmidt, M. Budde, A. L. Comes, K. Gade, M. Heilbronner, J. L. Kalman, F. Klöhn-Saghatolislam, M. O. Kohshour, S. Papiol, D. Reich-Erkelenz, S. K. Schaupp, E. C. Schulte, F. Senner, T. Vogl, J. Wiltfang, E. Reininghaus, P. Falkai, T. G. Schulze, H. Bickeböller, and U. Heilbronner. Stability over time of scores on psychiatric rating scales, questionnaires and cognitive tests in healthy controls. *BJPsych Open*, 8:e55, 2022.

N. Vasquez, A. Gall, P. H. Ellaway, and M. D. Craggs. Light touch and pin prick disparity in the International Standard for Neurological Classification of Spinal Cord Injury (ISNCSCI). *Spinal Cord*, 51:375–378, 2013.

N. R. Winter, J. Blanke, R. Leenings, J. Ernsting, L. Fisch, K. Sarink, C. Barkhau, D. Emden, K. Thiel, K. Flinkenflügel, et al. A systematic evaluation of machine learning–based biomarkers for major depressive disorder. *JAMA Psychiatry*, 81:386–395, 2024.
